# Distinct components of mRNA vaccines cooperate to instruct efficient germinal center responses

**DOI:** 10.1016/j.cell.2025.11.023

**Published:** 2025-12-16

**Authors:** Diana Castaño, Emily Bettini, Binod Kumar, Aleksey Chudnovskiy, Anna Siv, Giulia Protti, Sandra Nakadakari-Higa, Simona Ceglia, Nina De Luna, Joy E. Chiu, Katlyn Lederer, Shuk Hang Li, Hassaan Ibrahim, Hiromi Muramatsu, Thandiswa Mdluli, Edit Abraham, Sinem E. Sahingur, Ivan Maillard, Ying K. Tam, Sunny Shin, Scott E. Hensley, Jonathan J. Miner, Zoltan Lipinszki, Andrea Reboldi, Norbert Pardi, Roberto Spreafico, Gabriel D. Victora, Michela Locci

**Affiliations:** 1 Department of Microbiology, Perelman School of Medicine, University of Pennsylvania, Philadelphia, Pennsylvania, USA; 2 Institute for Immunology and Immune Health, Perelman School of Medicine, University of Pennsylvania, Philadelphia, Pennsylvania, USA; 3 Laboratory of Lymphocyte Dynamics, The Rockefeller University, New York, NY, USA; 4 Department of Biotechnology and Biosciences, University of Milano-Bicocca, Milan, Italy; 5 Department of Pathology, University of Massachusetts Chan Medical School, Worcester, MA, USA; 6 Division of Hematology/Oncology, Department of Medicine, Perelman School of Medicine, University of Pennsylvania, Philadelphia, PA, USA and Division of Hematologic Malignancies, Memorial Sloan Kettering Cancer Center, New York, NY, USA; 7 Synthetic and Systems Biology Unit, Institute of Biochemistry, HUN-REN Biological Research Centre, Szeged, Hungary; 8 National Laboratory for Biotechnology, Institute of Genetics, HUN-REN Biological Research Centre, Szeged, Hungary; 9 Department of Periodontics, School of Dental Medicine, University of Pennsylvania, Philadelphia, Pennsylvania, USA; 10 Acuitas Therapeutics, Vancouver, BC, Canada; 11 Division of Rheumatology, Department of Medicine, RVCL Research Center, Perelman School of Medicine, University of Pennsylvania, Philadelphia, Pennsylvania, USA; 12 ATGandCo Biotechnology Ltd, Mórahalom, Hungary; 13 Institute for Quantitative and Computational Biosciences, University of California, Los Angeles, USA; 14 Howard Hughes Medical Institute, New York, NY, USA; 15 These authors contributed equally; 16 Lead Contact

**Keywords:** mRNA vaccines, lipid nanoparticles, Tfh cells, germinal centers, interferons, dendritic cells, interleukin-1

## Abstract

Nucleoside-modified messenger RNA (mRNA) vaccines elicit protective antibodies through their ability to promote T follicular helper (Tfh) cells. The lipid nanoparticles (LNP) of mRNA vaccines possess inherent adjuvant activity. However, to what extent the nucleoside-modified mRNA is sensed and contributes to Tfh cell responses remains undefined. Herein, we deconvoluted the signals induced by LNP and mRNA that instruct dendritic cells (DCs) to promote Tfh cell differentiation. We demonstrated that the mRNA drives the production of type I interferons, which act on DCs to enhance their maturation and Tfh cell differentiation, and favors plasma cells and memory B cell responses. In parallel, LNP, which allows for mRNA uptake by DCs within the draining lymph node, also modulate Tfh cell responses by shaping the localization of CD25^+^ DCs. Our work unravels distinct adjuvant features of mRNA and LNP necessary for the induction of Tfh cells, with implications for rational vaccine design.

## In Brief

This study dissects the way the RNA and lipid nanoparticle components of the formulation individually contribute to the overall immune response to vaccination. Nucleoside modified mRNA promotes the production of type I interferons that act on dendritic cells to amplify T follicular helper cell and B cell activity, while the LNP component instructs discrete Tfh cell-inducing properties in dendritic cells that are required for optimal germinal center responses.

## Graphical Abstract

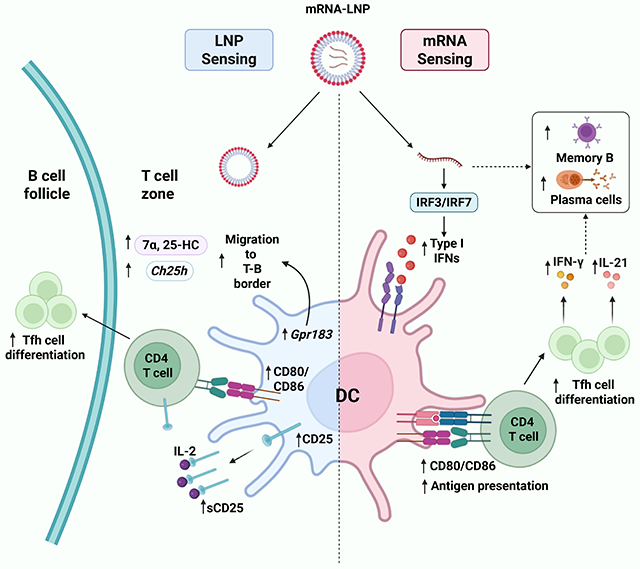

## INTRODUCTION

mRNA vaccines for severe acute respiratory syndrome coronavirus 2 (SARS-CoV-2) have proven to be successful at driving potent neutralizing antibody (nAb) and memory B cell (MBC) responses, as well as preventing severe coronavirus disease 2019 (COVID-19)^1^. Long-lived plasma cells (LLPCs), which secrete Abs, and MBCs are canonically generated through germinal center (GC) reactions^2^. Previous work has demonstrated that mRNA vaccines, comprised of nucleoside-modified and purified mRNA encapsulated in LNP (mRNA-LNP), drive potent GC responses in both mice and humans, which correlate with nAb production and antigen-specific MBC responses^3–6^. However, the mechanism by which mRNA-LNP instructs GC responses remains largely undefined.

GC reactions are regulated by a specialized subset of CD4 T cells referred to as T follicular helper (Tfh) cells^7–9^. Tfh cell differentiation begins when antigen-presenting cells (APCs), often DCs, present antigen to naïve CD4 T cells within the T cell zones of secondary lymphoid organs. During this interaction, DCs deliver to the CD4 T cells a multitude of signals that prompt the initiation of Tfh cell differentiation^7,9,10^, including costimulatory molecules and cytokines. Conversely, antagonistic signals driven by IL-2 and its high-affinity receptor CD25 can suppress the early differentiation of Tfh cells^11,12^. Activated CD4 T cells also upregulate the oxysterol receptor Ebi2, which allows for early positioning of these cells at the interface of the B cell follicles and T cell zones (T-B border), where they commit to the Tfh differentiation pathway by interacting with ICOSL^+^CD25^+^ DCs^13^.

We have previously demonstrated that the LNP component of mRNA vaccines has intrinsic adjuvant activity, which can favor Tfh cell differentiation through the induction of IL-6^4^. However, it is not completely understood whether signals important for the differentiation of Tfh cells also stem from the sensing of nucleoside-modified mRNA. The introduction of modified nucleosides such as pseudouridine that restrains the capacity of Toll-like receptor (TLR) 3 and TLR7/8 to induce pro-inflammatory cytokines such as type I interferons (IFNs) in response to the *in vitro* transcribed mRNA^14–17^ was believed to make the mRNA immunosilent. However, it has not been elucidated whether nucleoside-modified mRNA can trigger a low-level production of type I IFNs that is measurable using sensitive approaches and biologically relevant for Tfh cell responses. Our finding that mice lacking the TLR adaptor MyD88 mounted hindered Tfh cell responses following immunization with mRNA-LNP, but not upon injection of protein subunit antigens mixed with empty LNP^4^, spurred the hypothesis that the mRNA modification and purification do not completely abolish the capacity of pattern recognition receptors (PRRs) to recognize the mRNA component of mRNA-LNP vaccines.

Herein, we sought to uncouple the signals promoted by LNP from those driven by the mRNA that elicit optimal Tfh and GC responses. Our work uncovered the importance of type I IFNs, produced in response to the mRNA vaccine component via an IRF3-IRF7-dependent pathway, in promoting DC maturation and Tfh cell differentiation by acting directly on DCs. Notably, the mRNA influenced the functional profile of Tfh cells and favored a robust elicitation of MBCs and LLPCs. On the other hand, the sensing of LNP instructed a transcriptional program in DCs important to favor Tfh cell differentiation, which included costimulatory molecules, CD25, and the chemoattractant receptor Ebi2. Moreover, we found that most conventional DCs in the draining lymph node (dLN) efficiently internalized LNP and translated mRNA *in situ*, with only a minority of LN DCs that processed mRNA-LNP at the site of vaccine injection, as shown by photoactivation studies. Altogether, our work underscores a dual immunological mechanism of action of mRNA-LNP vaccines and unfolds distinct adjuvant properties of the mRNA and LNP vaccine components.

## RESULTS

### Type I IFNs driven by nucleoside-modified mRNA amplify GC reactions

**Figure 1.**
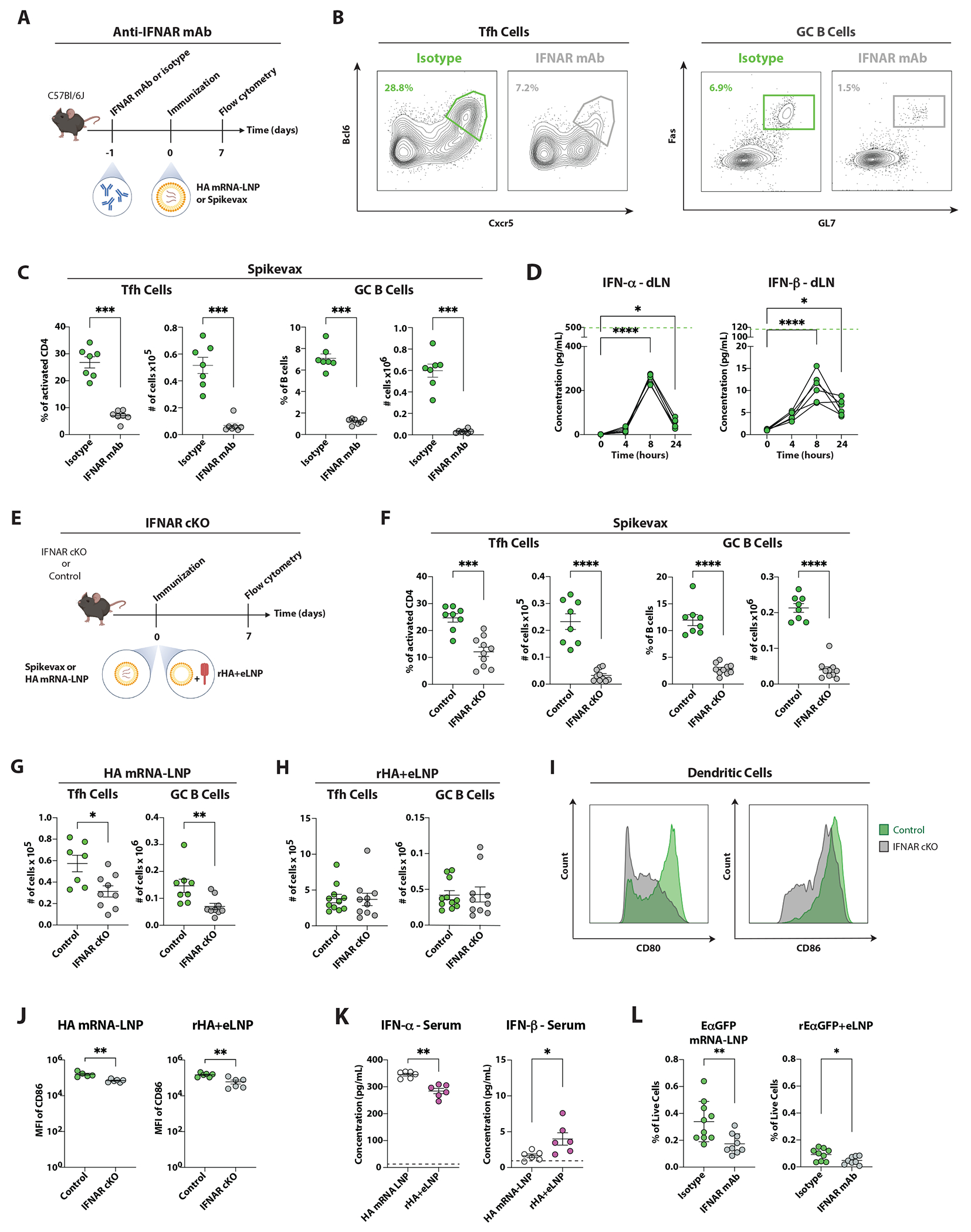
Type I IFN signaling in DCs amplifies GC responses to mRNA-LNP. **(A)** Experimental design of panels **B** and **C.** **(B)** Representative flow cytometry of Tfh cells and GC B cells. **(C)** Tfh and GC B cell frequency and absolute numbers, as detailed in **B**. **(D)** Kinetics of IFN-α and IFN-β production in dLNs. Green dotted lines represent the mean IFN-α and IFN-β induction by Poly (I:C) 8 hours post-injection. **(E)** Experimental design of panels **F-H.** **(F)** Tfh and GC B cell frequency and absolute numbers, analyzed as in **B**. **(G and H)** Tfh and GC B cell absolute numbers post-immunization. **(I)** Representative expression of CD80 and CD86 by DCs 24 hours post-immunization. **(J)** Quantification of CD86 expression, as detailed in **I**. **(K)** IFN-α and IFN-β levels 8 hours post-immunization. Black dotted lines represent the mean values from naive mice. **(L)** Frequency and absolute numbers of antigen-presenting DCs 24 hours after immunization. Mice received 3 μg of Spikevax in (**B-C, F**), 5 μg of Spikevax in (**D**), 30 μg of HA mRNA-LNP or rHA+eLNP in (**G-K**), and 20 μg of EαGFP mRNA-LNP or rEαGFP+eLNP in (**L**). In all graphs, n = 6-11 mice per group combined from 2-3 independent experiments. An unpaired two-tailed Mann-Whitney *U* test was conducted.

Type I IFNs (IFN-α and IFN-β) can enhance humoral immunity and Tfh cell responses by stimulating DC maturation and the production of the pro-Tfh cytokine IL-6^18–21^. To determine if nucleoside-modified mRNA-LNP can drive the expression of type I IFNs that influence the generation of Tfh and GC B cell responses, we administered an interferon receptor 1 (IFNAR) blocking antibody to mice prior to immunization with a custom-made influenza virus hemagglutinin (HA) (HA mRNA-LNP) vaccine^4^ (30 μg/mouse, Figure 1A). In the absence of IFNAR signaling, the generation of GC responses was hampered, as demonstrated by a decrease in the number of Tfh cells and GC B cells (Figure S1A–C). However, the frequency of both cell types remained intact. Similar trends were observed with another custom-made mRNA vaccine encoding for the receptor-binding domain (RBD) of SARS-CoV-2 (RBD mRNA-LNP) (Figure S1D). To determine whether type I IFNs are relevant for GC responses to licensed mRNA-LNP vaccines, we immunized mice with Moderna’s COVID-19 mRNA vaccine (Spikevax, Figure 1A–C). A low dose (3 μg/mouse) of Spikevax drove robust Tfh and GC B cell responses that were severely hindered in frequency and absolute numbers upon IFNAR blockade. In line with these data, a Spikevax-driven IFN-α and IFN-β production was observed using a high-sensitivity assay in dLN, serum, and at the injection site, peaking at 8 hours post-injection (Figures 1D and S1E–F). By contrast, when IFNAR was blocked in mice that received a low dose of HA mRNA-LNP vaccine, the impact of type I IFNs in Tfh cell responses was lost (Figure S1G), suggesting that the ability of mRNA-LNP vaccines to drive a biologically relevant production of type I IFNs may vary, based on mRNA chemistry/composition and/or production process. Together, these data indicate that nucleoside-modified mRNA-LNP vaccines can drive the production of type I IFNs that are key to generating optimal GC responses.

To test if IFNAR signaling in DCs is responsible for the defective GC responses, as observed in certain models^18,19,21^, we immunized mice lacking IFNAR in DCs (*Cd11c-cre Ifnar^flox/flox^*; IFNAR cKO) and controls (*Cd11c-cre Ifnar^wt/wt^*) with Spikevax (Figure 1E). IFNAR cKO mice displayed a pronounced decrease in Tfh and GC B cell responses, highlighting the importance of type I IFN signaling in DCs for the formation of efficient GC responses to Spikevax (Figure 1F). Next, IFNAR cKO mice were immunized with HA mRNA-LNP or with HA protein mixed with empty LNP (rHA+eLNP) to evaluate the contribution of the nucleoside-modified mRNA to the IFN-driven GC amplification (Figure 1E). We found that IFNAR cKO mice immunized with HA mRNA-LNP had normal frequencies along with lower numbers of Tfh and GC B cells (Figure 1G and S1H). By contrast, IFNAR cKO mice immunized with HA+eLNP had intact GC responses (Figures 1H and S1H), suggesting a significant contribution of the mRNA component to the IFNAR-regulated GC responses in mRNA-LNP vaccination.

We next investigated whether the absence of IFNAR signaling impacts the maturation of DCs, measured by the expression of the costimulatory molecules CD80 and CD86^22^. DCs lacking IFNAR had a marked decrease in the expression of CD80 and CD86 after immunization with HA mRNA-LNP (Figures 1I–J and S1I). The maturation of DCs was also impaired in IFNAR cKO mice immunized with rHA+eLNP, in line with the observation that type I IFNs can also be produced in response to the LNP component (Figure 1K). Lastly, we sought to determine whether type I IFN modulates antigen presentation of EαGFP, an immunogen that allows for the simultaneous tracking of antigen uptake and presentation via MHC II molecules^23,24^. Of note, this approach uncovered a significant reduction in antigen presentation when IFNAR was blocked in mice immunized with EαGFP mRNA-LNP, and to a lesser extent, in mice immunized with rEαGFP+eLNP (Figures 1L and S1J). Taken together, these data suggest that the *in vivo* sensing of nucleoside-modified mRNA contributes to the adjuvant effect of mRNA-LNP vaccines by favoring a type I IFN-dependent DC maturation/antigen-presentation that, in turn, promotes optimal Tfh cell and GC responses.

### IRF3/IRF7 regulate type I IFN-dependent GC responses to mRNA-LNP.

**Figure 2.**
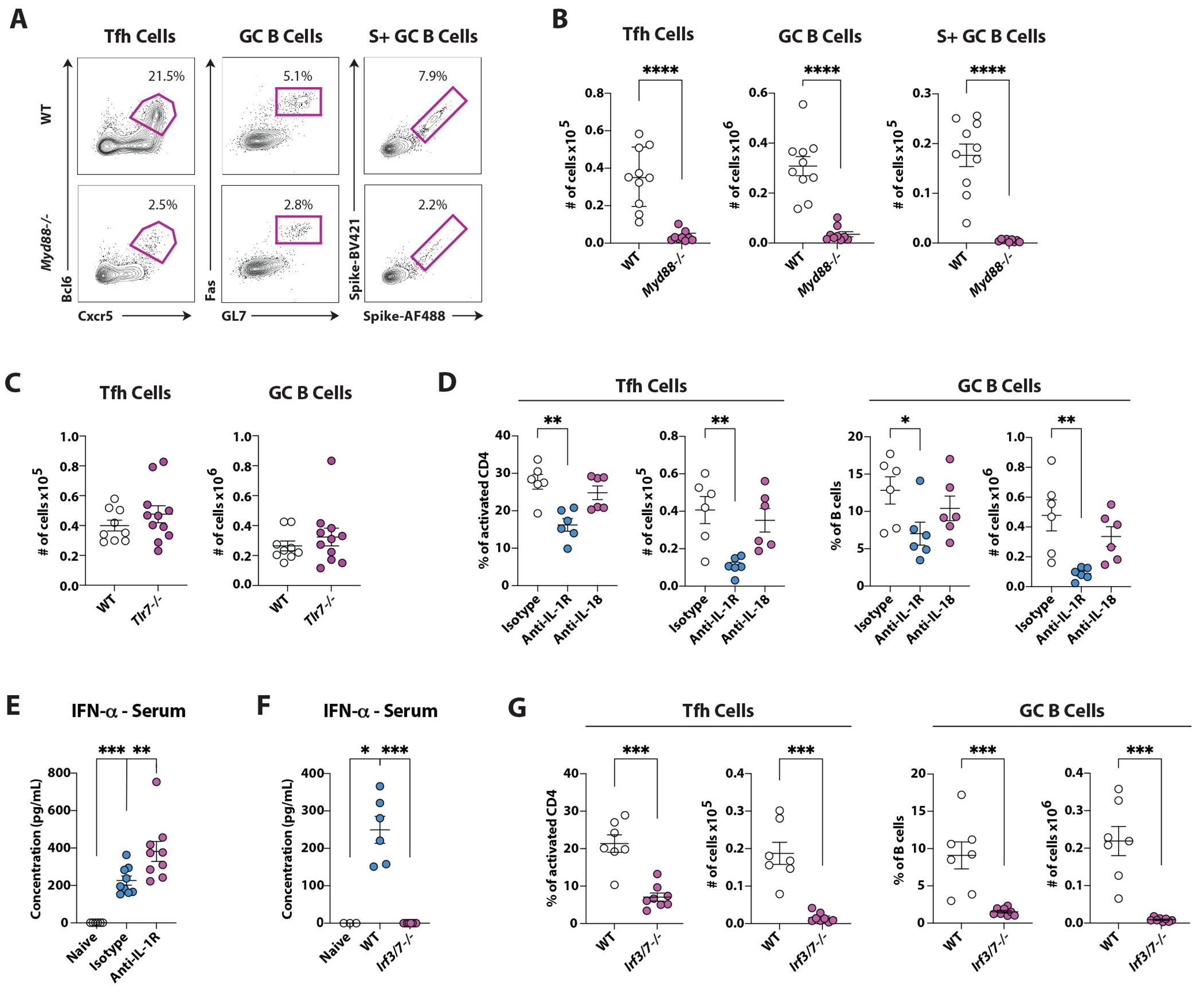
IRF3/7 are required for type I IFN-mediated GC responses to Spikevax. **(A)** Representative flow cytometry of Tfh cells, GC B cells, and Spike-specific GC B cells (S+) from wild type (WT) and *Myd88*^−/−^ mice. **(B)** Tfh cell, GC B cell, and S+ GC B cell absolute numbers, as detailed in **A**. **(C)** Absolute numbers of Tfh cells and GC B cells, as detailed in **A**, in WT and *Tlr7*^−/−^ mice. **(D)** Frequencies and absolute numbers of Tfh cells and GC B cells, as detailed in **A**, in mice treated with isotype, anti-IL-1R, or anti-IL-18 mAb. **(E)** IFN-α levels 8 hours after immunization. Mice were treated with isotype or anti-IL-1R mAb. **(F)** IFN-α levels in WT and *Irf3/7−/−* mice 8 hours after immunization. **(G)** Frequency and absolute numbers of Tfh cells and GC B cells, as detailed in **A**, in WT and *Irf3/7−/−* mice. In (**A-G**), mice received 3 μg of Spikevax; n = 6-10 mice per group from 2-3 independent experiments. An unpaired two-tailed Mann-Whitney *U* test was conducted.

MyD88 is a common adaptor downstream of most TLRs^25^. We found that, similar to what was seen with our custom mRNA-LNP vaccine^4^, MyD88 was critical for eliciting Tfh cells and GC B cells upon Spikevax immunization (Figures 2A–B and S2A). Therefore, we asked if TLR7, a TLR activated by single-stranded RNA (ssRNA) and signaling via MyD88^25^, can sense nucleoside-modified mRNA. No relevant activation of the IRF pathway downstream of TLR7 was detected in a reporter cell line cultured with Spikevax or HA mRNA-LNP (Figure S2B). Moreover, Tfh and GC B cell responses were not decreased in TLR7-deficient mice immunized with Spikevax or HA mRNA-LNP (Figures 2C and S2C–D). Certain LNP compositions can activate TLR4^26^, which could also require MyD88 for its downstream signal^25^. We could not find any detectable signals in response to either Spikevax or HA mRNA-LNP in a TLR4 reporter cell line at the time points tested (Figure S2E), nor any alterations of Tfh and GC B cell responses in TLR4-deficient mice immunized with Spikevax (Figure S2F). Similarly, no defect in GC responses was observed in mice deficient for TLR2, a lipoprotein sensor (Figure S2F). Lastly, we asked whether double-stranded RNA (dsRNA) contaminants or secondary structures in the nucleoside-modified mRNA could activate TLR3, even though this TLR signals via a MyD88-independent pathway^25^. We could not find a relevant activation of TLR3 using a reporter cell line, nor a defective GC response in *Tlr3* knock-out mice in response to Spikevax (Figure S2G–H). Whilst our custom-made HA mRNA vaccine was able to elicit the activation of the IRF pathway in the TLR3 reporter line, TLR3 detection of this modified mRNA was not a requirement for the induction of GC responses *in vivo* (Figure S2I–J). Overall, our data suggest that these TLRs cannot sense the vaccine formulations tested here *in vitro* and are not overtly involved in driving GC responses to these mRNA-LNP vaccines.

Since MyD88 is also a key mediator of signal transduction downstream of interleukin-1 receptor (IL-1R)^27^, we hypothesized that IL-1α and/or IL-1β modulate Tfh cell differentiation in response to Spikevax. Blockade of IL-1R resulted in a significant impairment of GC responses to this mRNA vaccine (Figure 2D), whereas blockade of IL-18, also relying on MyD88 to signal^27^, did not lead to a significant decrease in Tfh and GC B cells (Figure 2D). Interestingly, however, the blockade of IL-1R did not hinder IFN-α production (Figure 2E). Altogether, these data indicate that the IL-1/MyD88 axis is relevant for GC responses induced by Spikevax in a type I IFN-independent fashion.

Viral RNA recognition by the cytosolic RNA sensors RIG-I or MDA-5 can promote the activation of IRF3 and IRF7, leading to type I IFN production^28^. Hence, we asked whether IRF3 and IRF7 are important for the regulation of GC responses by type I IFNs following Spikevax immunization. Of note, *Irf3-Irf7* double knock-out mice presented with a complete suppression of IFN-α that was coupled with abolished GC responses (Figure 2F–G). Next, we investigated whether RIG-I can sense components of the Spikevax vaccine *in vitro* and modulate GC responses *in vivo*. No RIG-I-driven IRF activation was measurable *in vitro* using a reporter cell line (Figure S2K), and normal Tfh and GC B cell responses were found in RIG-I-deficient mice (Figure S2L). Similarly, Spikevax did not significantly activate MDA5-driven IRF *in vitro* in a reporter cell line (Figure S2M). To rule out MDA5 requirement for *in vivo* GC responses to Spikevax, we immunized mice lacking MAVS, a key adaptor protein required for MDA5 (and RIG-I) signaling^28^. Consistent with studies with a different mRNA-LNP formulation^4^, GC responses were largely normal in MAVS-deficient mice (Figure S2N). Altogether, these data show no direct involvement of RIG-I-like receptors (RLRs) in GC responses after Spikevax immunization.

The cGAS/STING pathway is a potent sensor of cytosolic DNA accumulation^29^ and could recognize contaminant DNA in mRNA-LNP formulations and drive type I IFN production via IRF3. We immunized STING Goldenticket (STING GT) mice^30^ with Spikevax and found no relevant decrease in the magnitude of GC responses in these STING-deficient mice (Figure S2O).

Collectively, our data point to the importance of the IRF3/IRF7-type I IFN axis in regulating GC responses against Spikevax.

### The nucleoside-modified mRNA influences Tfh cell function and enhances B cell responses

**Figure 3.**
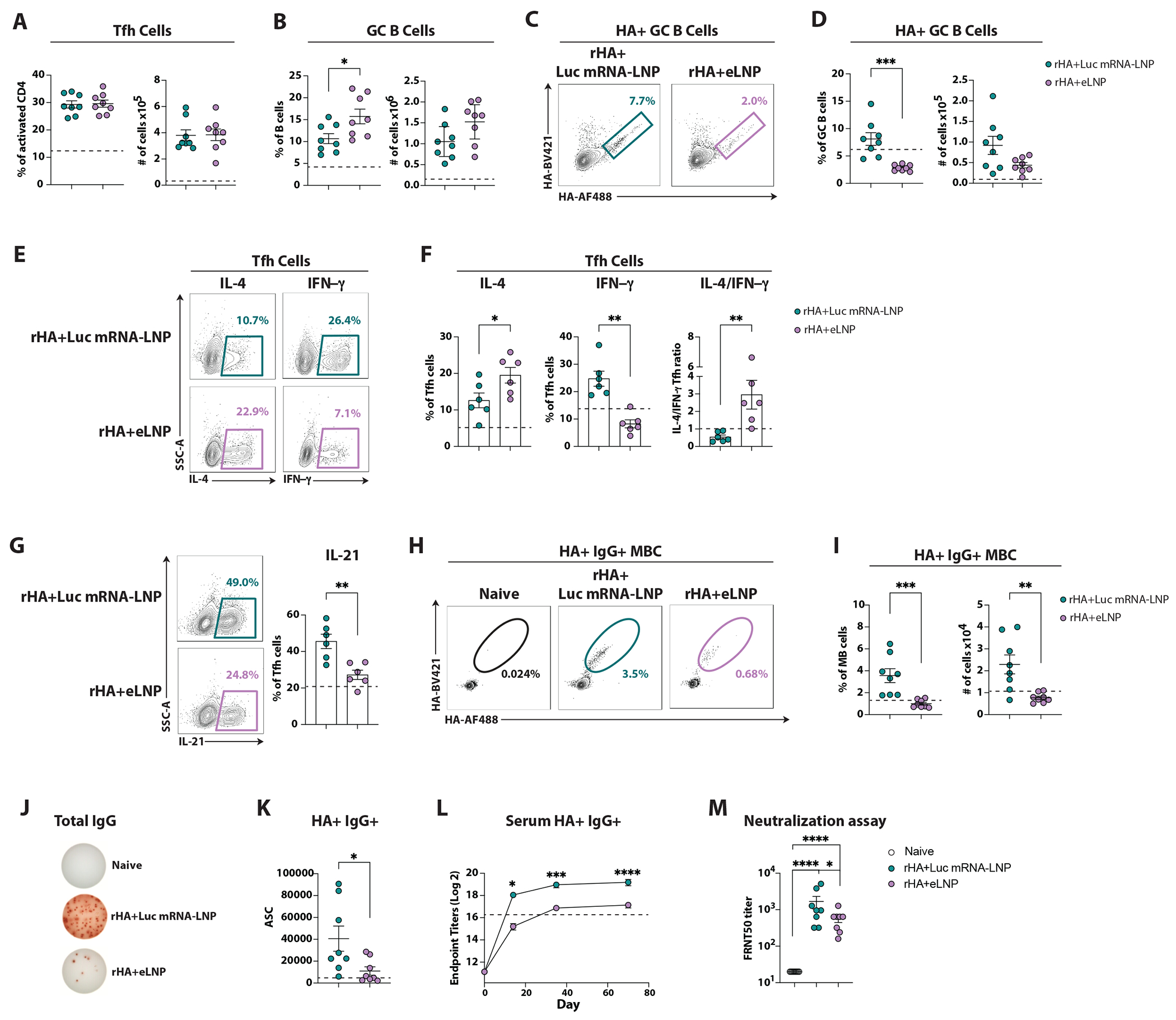
Nucleoside-modified mRNA modulates Tfh cell function and enhances B cell responses. **(A)** Tfh cell frequency and absolute numbers. **(B)** GC B cell frequency and absolute numbers. **(C)** Representative flow cytometry of HA-specific GC B cells (HA+). **(D)** HA+ GC B cell frequency and absolute numbers, as detailed in **C**. **(E)** Representative IL-4 and IFN-γ expression in Tfh cells. **(F)** Quantification of IL-4 and IFN-γ-producing Tfh cells, and of IL-4+/IFN-γ+ Tfh cell ratio from **E**. **(G)** Representative flow cytometry and quantification of IL-21+ Tfh cells. **(H)** Representative flow cytometry of HA+ IgG+ MBCs. **(I)** Quantification of HA+IgG+ MBC frequency and absolute numbers from **H**. **(J)** Representative HA+IgG+ ASCs. **(K)** Quantification of HA+ IgG+ ASCs from **J**, represented as the number of ASCs per 10^^6^ bone marrow cells. **(L)** Endpoint titers of HA-specific IgG. **(M)** Focus reduction neutralization test (FRNT) assay. In (**A-M**), mice were immunized with 30 μg rHA+Luc mRNA-LNP or 30 μg rHA+eLNP. Black dotted lines represent the mean values of mice immunized with 30 μg HA mRNA-LNP. In all experiments, n = 6-8 mice per group from 2-3 independent experiments. In (**A-B, D, F, G, I, K**), an unpaired two-tailed Mann-Whitney *U* test was performed, in (**L**), a two-way ANOVA was performed, and in (**M**), an unpaired one-tailed Mann-Whitney *U* test was performed.

Next, we sought to determine if/how the nucleic acid modulates the magnitude and quality of GC responses to mRNA-LNP vaccines. Hence, we compared the immune responses in mice immunized with rHA+eLNP or rHA mixed with LNP containing a nucleoside-modified mRNA encoding for firefly luciferase (rHA+Luc mRNA-LNP). As a control, animals immunized with HA-mRNA-LNP were also included (dotted line). Total and HA-specific Tfh cell responses were not significantly different between immunizations (Figures 3A and S3A–B). Conversely, total GC B cell responses were modestly restrained by the nucleoside-modified mRNA (Figure 3B). Nonetheless, the presence of Luc mRNA favored the frequency of HA-specific GC B cells, ultimately leading to a similar number of antigen-specific cells (Figure 3C–D).

Different vaccine modalities can drive the generation of functionally distinct Tfh cells^5^. Thus, we asked whether Tfh cell responses elicited by rHA+eLNP or rHA+Luc mRNA-LNP were qualitatively different. CXCR5^+^ Tfh cells (including both GC Tfh and Tfh cells^31^) from dLNs were sorted before restimulation (Figures 3E–F and S3C). The presence of nucleoside-modified mRNA modestly restrained the frequency of IL-4^+^Tfh cells. By contrast, Luc mRNA favored a higher frequency of IFN-γ^+^ Tfh cells and a higher production of IFN-γ on a per cell basis (Figures 3E–F and S3D). Sensing of type I IFNs by DCs can either favor or antagonize the generation of IFN-γ^+^ CD4 T cells^32^. To determine whether type I IFN signaling in DCs is responsible for an enhanced IFN-γ production by CD4 T cells, we immunized IFNAR cKO and controls with the RBD mRNA-LNP vaccine. IFNAR cKO mice had a significant decrease in IFN-γ-producing CD4 T cells and Tfh cells in comparison to control mice, whereas the IL-4 production was intact (Figure S3E), pointing to a model where the mRNA-driven type I IFNs act on DCs to enhance IFN-γ production by CD4 T cells. Next, we evaluated the production of IL-21, a key cytokine that supports the generation of affinity-matured B cell responses^8,9^. The presence of nucleoside-modified mRNA led to a higher frequency of IL-21-producing Tfh cells (Figure 3G).

The finding that nucleoside-modified mRNA influenced the function of Tfh cells prompted us to compare the magnitude of antigen-specific MBCs (Figure S3F) and antibody-secreting cells (ASC) in rHA+eLNP and rHA+Luc mRNA-LNP vaccination. The immunostimulatory properties of the mRNA enhanced the IgG^+^ HA-specific MBC responses (Figure 3H–I). An increase in IgG2c^+^ cells accounted for the higher number of HA-specific MBC, whereas the number of IgG1^+^ HA-specific MBC was not influenced by the presence of the mRNA (Figure S3G–H). Similarly, the mRNA prompted overall stronger ASC responses (Figure 3J–K) characterized by enhanced HA-specific ASC of the IgG2c isotype (Figure S3I–J). In line with these data, Luc mRNA-LNP-immunized mice generated higher HA-specific IgG serum titers (Figure 3L), comparable IgG1 and significantly higher IgG2c (Figure S3K) when compared to the rHA+eLNP immunized mice. Of note, immunization with rHA+Luc mRNA-LNP yielded superior neutralization titers (Figure 3M).

Altogether, these data indicate that the nucleoside-modified mRNA can drive a pronounced enhancement of IFN-γ and IL-21-producing Tfh cells that are associated with superior MBC and ASC induction.

### LNP instruct distinct transcriptional states in DCs

**Figure 4.**
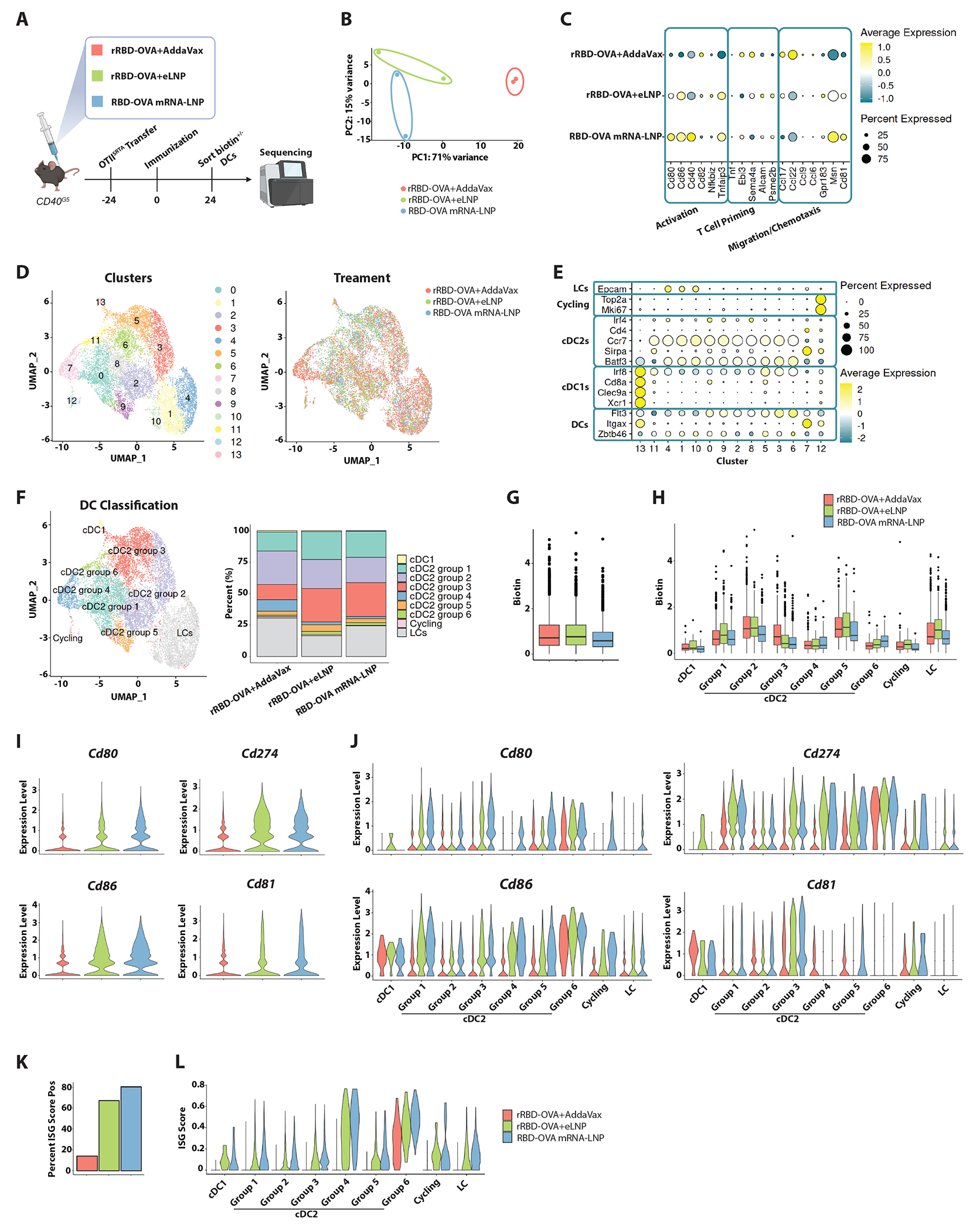
LNP-adjuvanted vaccines drive distinct gene expression profiles. **(A)** LIPSTIC experimental design. **(B)** PCA of single-cell RNA sequencing from **A**. Each point represents an individual mouse. **(C)** Dot plot indicating expression level of activation, T cell priming, and migration/chemotactic profiles driven by immunization from **A**. **(D)** CCA-integrated UMAP clustering displayed as unsupervised clusters or by immunization from **A**. **(E)** Dot plot displaying the gene list used to define clusters in (**D)**. **(F)** DC classification of UMAP clusters based on gene expression profiles in **E**, also displayed as the frequency of each treatment group. (**G and H**) Frequency of biotin labeling based on groups (**G**) and on the different DC clusters (**H**) from **A**. (**I and J**) Expression of the indicated genes in each group (**I**) and on the different DC clusters (**J**) from **A**. **(K)** Interferon-stimulated gene (ISG) signature score in each group from **A**. **(L)** ISG signature in DC group from **F**. In (**A-L**), mice were immunized with 30 μg of RBD-OVA mRNA-LNP, 30 μg of rRBD-OVA+eLNP, or 30 μg of rRBD-OVA+AddaVax; n = 2 mice per group.

To evaluate the impact of the nucleoside-modified mRNA and LNP vaccine components on the transcriptional program of DCs, we deployed the LIPSTIC system and probed DC transcriptional profile in relation to their antigen-presenting status^33,34^. The LIPSTIC technology relies on the transfer of a biotinylated substrate from TCR transgenic OT-II cells, expressing *Staphylococcus aureus* transpeptidase sortase A (SrtA) bound to CD40L (CD40L^SrtA^), to acceptor DCs expressing a five N-terminal glycine residue tag on CD40 (CD40^G5^) upon antigen presentation. We designed mRNA and recombinant protein where the SARS-CoV-2 RBD was fused to the ovalbumin peptide recognized by OT-II cells (referred to as RBD-OVA) (Figure 4A and S4A and B). OT-II-CD40L^SrtA^ cells were transferred into CD40^G5^ mice prior to immunization with either RBD-OVA mRNA-LNP, recombinant RBD-OVA with empty LNP (rRBD-OVA+eLNP), or recombinant RBD-OVA with AddaVax (rRBD-OVA+AddaVax; a comparator used previously^4,5^). The transcriptional profiles of DCs were determined via single-cell RNA sequencing post-immunization. Of note, the principal component analysis (PCA) of total (biotin^+^ and biotin^−^) DC revealed that the largest contributor to the variance was the presence of LNP in the vaccine formulation (PC1, over 70%), whereas the presence of mRNA accounted for only 15% of the variance in PC2 (Figure 4B). A UMAP visualization confirmed that DCs from mice immunized with rRBD-OVA+AddaVax displayed discrete and largely non-overlapping clusters in comparison to LNP-adjuvanted vaccines (Figure S4C). A dot plot of activation markers along with molecules involved in T cell priming and migration/chemotaxis showed differential expression in the LNP-adjuvanted groups compared to the AddaVax group (Figure 4C). This analysis, along with a direct comparison of DC activation in mice immunized with rHA+eLNP or rHA+Luc mRNA-LNP (Figure S4D) and our earlier data (Figure 1), confirms that both the LNP and the nucleoside-modified mRNA components contribute to DC maturation. When evaluating the distribution of biotin^+^ DCs (indicative of antigen presentation to OT-II cells), we observed that the biotin signal was spread out across most clusters in LNP-adjuvanted groups (Figure S4E). Different from what was observed with a weaker adjuvant (Alum) or tumor-presenting DCs^33,34^, this broad distribution suggests that strong adjuvants, like LNP, activate most dLN DCs, dwarfing the effect driven by cognate T cell help on DCs (CD40L-driven engagement of CD40 on DCs, which favors DC maturation^35,36^).

Several subtypes of DCs endowed with distinct T cell priming properties exist^37^. While type 1 conventional DCs (cDC1) are effective at cross-presentation to CD8 T cells, type 2 conventional DCs (cDC2) are specialized in antigen presentation to CD4 T cells and induction of Tfh cells^22,38^. Canonical Correlation Analysis (CCA) in Seurat^39^ was performed to integrate transcriptomic data from the three immunization conditions and generated clusters that were, for the most part, evenly represented in each immunization condition (Figure 4D). A curated gene list (Figure 4E) was used to determine the types of DCs that resided in each cluster, leading to the definition of cDC1, cDC2, Langerhans cells (LCs), and cycling cells. Upon renaming the UMAP clusters accordingly (Figure 4F), most cells were cDC2s of differing transcriptional states. We therefore assigned the cDC2s to 6 different groups based on their top differentially expressed genes (Figure S4F). The biotin signal was similarly distributed across the protein-adjuvanted vaccine groups, with a trend for a modest decrease in biotin enrichment in the mRNA-LNP group (Figure 4G). As anticipated, the cells with the highest biotin expression showed a trend for enrichment in the LC cluster and selected cDC2 groups (Groups 1, 2, and 5; Figure 4H). Additionally, a GSEA approach confirmed the enrichment of a previously published LIPSTIC gene signature^34^ in biotin^+^ DCs (Figure S4G–H).

We next asked whether the gene signatures of activation and migration observed in LNP-vaccinated animals (Figure 4C) were restricted to specific DC populations. We found a marked increase in CD80 and CD86 expression in cDC2s from the LNP-containing groups, when compared to the AddaVax group (Figure 4I and J). Among the cDC2 groups with the highest expression of *Cd80* and *Cd86* (groups 1, 4, and 6), only group 1 was enriched in biotin expression (Figure 4H and J), suggesting that DCs that did not yet present antigen gained enhanced maturation as a result of the mRNA-LNP immunostimulatory activity. Regulators of migration were also robustly driven by LNP-containing vaccines (Figures 4I–J and S4I–J), as indicated by a induction of *Cd274* (the gene encoding for PD-L1, which is also important for the migratory capacity of DCs^40^), *Cd81* (a regulator of DC motility^41^), and *Cxcr5* (a chemokine receptor important for the migration of DCs toward B cell follicles^42^), in many cDC2 groups.

Finally, given the connection between type I IFNs and mRNA sensing (Figure 1), we asked whether an enrichment in an interferon-stimulated gene (ISG) signature was present in the animals immunized with mRNA-LNP. To this aim, we utilized an ISG signature consisting of the top 20 differentially expressed genes in DCs cultured with IFN-α *in vitro*^43^. We found a robust ISG signature in the RBD-OVA mRNA-LNP immunization condition, along with a trending lower ISG signature in the rRBD-OVA+eLNP immunization group (Figure 4K and L).

Taken together, these data suggest that the LNP component of mRNA vaccines is a powerful immunostimulator that drives activation and migration programs in cDC2s.

### LNP induce a pro-Tfh cell program in cDC2s and guide Tfh cell differentiation via an Ebi2-oxysterol axis

**Figure 5.**
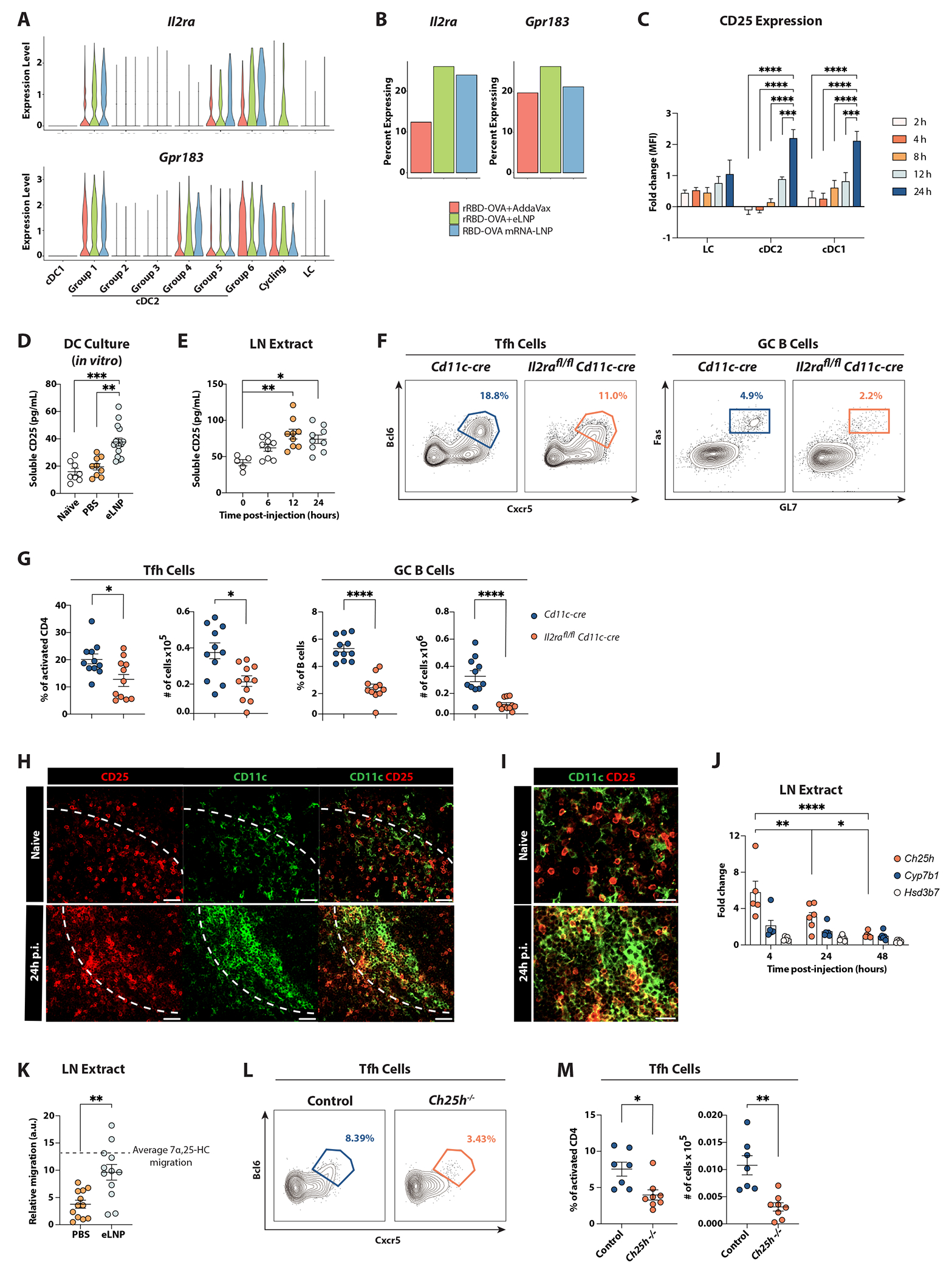
LNP-adjuvanted vaccines drive CD25 and Ebi2 expression in DCs. **(A)** Violin plots of *Il2ra* and *Gpr183* in DC clusters from Figure 4F. **(B)** Proportion of *Il2ra*- and *Gpr183*-expressing cells from Figure 4 RNA-sequencing data. **(C)** Surface expression of CD25 on DC subsets, represented as fold-change over baseline expression (0 hours, h). **(D)** Soluble CD25 produced *in vitro*. DCs isolated post-injection of eLNP or PBS. **(E)** Soluble CD25 produced *in vivo* from dLN extracts post eLNP injection. **(F)** Representative flow cytometry of Tfh and GC B cells. **(G)** Tfh and GC B cell frequency and absolute numbers, as detailed in **F**. **(H)** Representative microscopy images of dLN. The dashed line represents a T-B border. Scale bar, 25 μm. **(I)** Magnified T-B border from **H**. Scale bar, 15 μm. **(J)** qPCR analysis in dLN extracts after eLNP injection. Data is displayed as fold change versus naïve mice. **(K)** Cell migration in response to lipid extracts from dLNs of mice injected with PBS or eLNP. Dotted line denotes average migration to 7α,25-HC. **(L)** Representative flow cytometry of Tfh cells. **(M)** Quantification of Tfh cell frequency and number from **L**. Mice received 20 μg of eGFP mRNA-LNP-DiI (**C**), eLNP or PBS (**D-E and K**), 3 μg of Spikevax (**F-G**), 30 μg of HA mRNA-LNP (**H-I**), or 30 μg of RBD mRNA-LNP (**J** and **L-M**). (**C** and **J**) Two-way ANOVA was performed with Tukey’s correction for multiple comparisons. Only sample groups were compared versus all time points. (**D** and **E**) Kruskal-Wallis test was performed with Dunn’s correction for multiple comparisons. (**G, K,** and **M**) An unpaired two-tailed Mann-Whitney *U* test was conducted. In all experiments, n = 2-12 mice per group from 2-3 independent experiments.

We next sought to determine if LNP were capable of efficiently inducing a “pro-Tfh” program in cDC2s. Since groups 4 and 6 cDC2s expressed high levels of activation, migration, and ISG signatures and were biotin^lo^ (hence the transcriptional changes did not result from T cell help following antigen presentation), we asked what additional pro-Tfh genes were enriched in these groups in response to LNP-adjuvanted vaccines. DCs positioned at T-B borders can promote the differentiation of Tfh cells by interacting with Ebi2-expressing activated CD4 T cells and producing a soluble form of CD25 that quenches IL-2, a potent inhibitor of Tfh cell differentiation^11–13^. Interestingly, we found that the expression of *Il2ra* (CD25 encoding gene) and *Gpr183* (Ebi2 encoding gene) displayed higher enrichment in cluster 6, whereas cluster 4 showed heightened expression of *Grp183* but not of *Il2ra*, suggesting discrete pro-Tfh properties of these two clusters (Figure 5A). Moreover, both genes were expressed in DCs from mice immunized with LNP-containing vaccines (Figure 5B), further indicating that LNP might inherently drive a “pro-Tfh” program in DCs.

To gain further insights into how LNP shape the pro-Tfh program in cDC2s, we evaluated the induction of CD25 in DCs upon *in vivo* administration of mRNA-LNP. We observed an increase in the surface level of CD25 on all DC types, which peaked 24 hours after immunization and was more overt in cDC1 and cDC2 than in LCs (Figure 5C and S5A). Furthermore, we demonstrated that the LNP vaccine component was sufficient to promote the production of the soluble form of CD25 in the supernatant from DCs isolated from dLNs (Figure 5D) or from total LN extract (Figure 5E). To verify the importance of CD25 expression by DCs in driving Tfh cell differentiation to mRNA vaccines, we obtained mice that lack CD25 expression in CD11c^+^ cells. Tfh cell differentiation and GC B cell responses were hindered in *Cd11c-cre il2ra^flox/flox^* mice after Spikevax administration (Figure 5F–G). These data demonstrate that, as previously shown in other immunization settings^13^, LNP favor Tfh cell differentiation by promoting IL-2 quenching through CD25 production by DCs.

Next, we interrogated the localization of the CD25-expressing DCs in mRNA vaccination. Microscopy studies revealed an enrichment in CD25^+^ DCs in areas of the T cell zone proximal to B cell follicles after the injection of HA mRNA-LNP (Figure 5H–I and S5B). Ebi2 expression is critical for the positioning of DCs and CD4 T cells at the T-B borders of secondary lymphoid organs^9,13,44,45^. We found that the *in vivo* administration of eLNP led to upregulated expression of *Ch25h* and *Cyp7b*, which are enzymes required for the conversion of cholesterol into the Ebi2 ligand 7α, 25-dihydroxycholesterol (7α, 25-HC)^46,47^, but not *Hsd3b7*, which further metabolizes 7α, 25-HC into a derivative that can no longer bind Ebi2^46,47^ (Figure 5J). Next, to determine if the increased *Ch25h* and *Cyp7b1* expression was associated with an increase in Ebi2 ligands, we adopted a previously described *in vitro* migration bioassay^48^. The injection of eLNP increased the production of Ebi2 ligands *in vivo* (Figure 5K), as measured by the more robust migration in response to the lipid extract from the eLNP-injected mice in comparison to the PBS-treated group. To ascertain if the heightened production of Ebi2 ligands driven by LNP is relevant to Tfh cell differentiation *in vivo*, we immunized mice lacking CH25H (*Ch25h^−/−^*) with mRNA-LNP. Our experiment revealed a decrease in the relative and absolute numbers of Tfh cell responses in *Ch25h^−/−^* mice compared with control mice (Figure 5L–M). Overall, our data indicate an essential role of LNP-promoted 7α, 25-HC production in driving effective GC responses following mRNA-LNP immunization.

### Conventional DCs efficiently internalize mRNA-LNP

**Figure 6.**
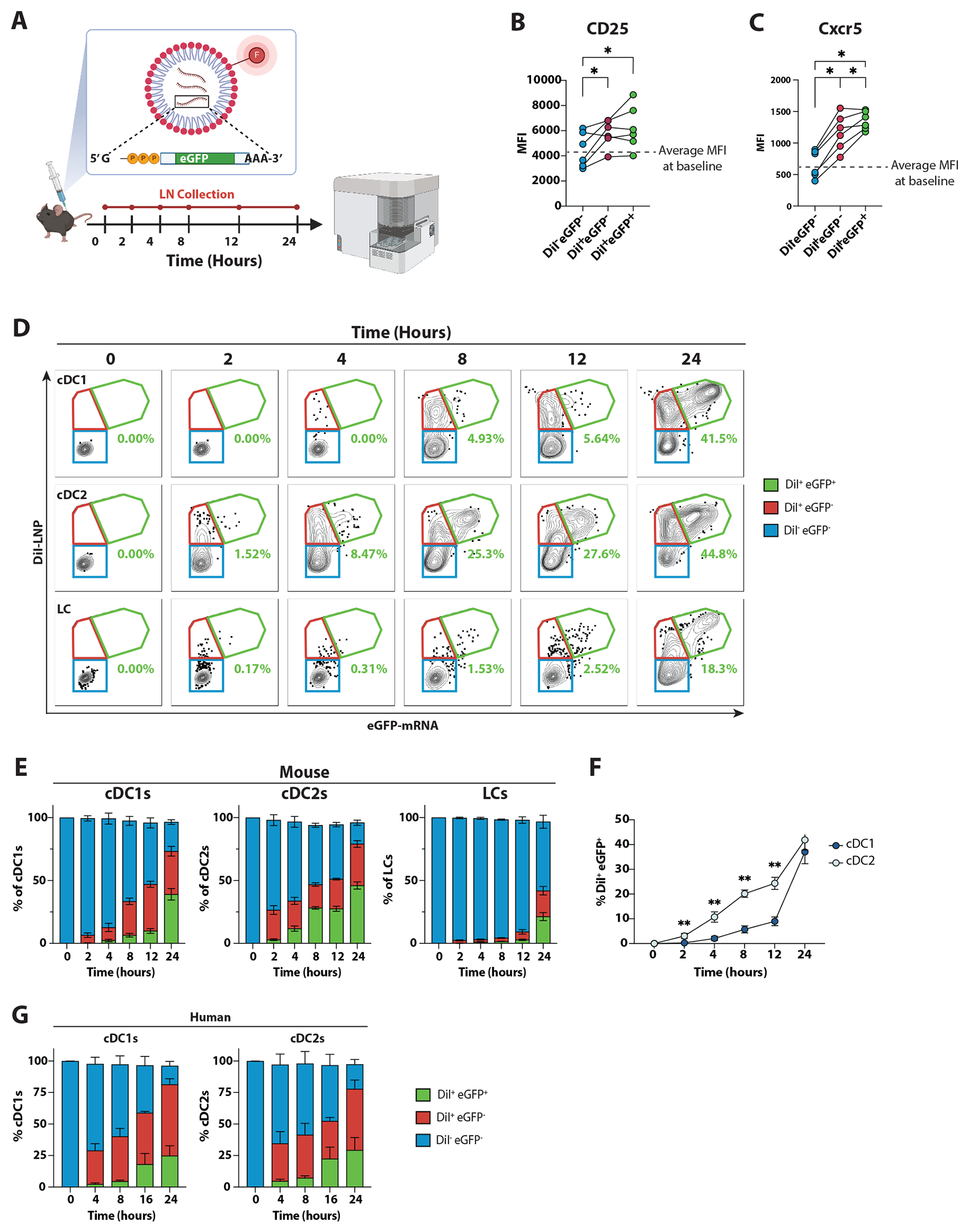
Conventional DCs are efficient at internalizing LNP and translating mRNA. **(A)** Experimental design of panels **B-F**. **(B and C)** CD25 **(B)** and Cxcr5 **(C)** expression in DiI^−^eGFP^−^, DiI^+^eGFP^−^, and DiI^+^eGFP^+^ cDC2s, 24 hours post-immunization. **(D)** Representative LNP binding/uptake (DiI^+^) and mRNA-encoding protein expression (eGFP^+^) in cDC1s, cDC2s, or LCs from dLN. **(E)** Quantification of **D**. Color represents the portion of cells that are DiI^+^eGFP^+^ (green), DiI^+^eGFP^−^ (red), and DiI^−^eGFP^−^ (blue). **(F)** Frequency of DiI^+^eGFP^+^ cDC1s and cDC2s. **(G)** Quantification of binding/uptake and expression of eGFP mRNA-LNP-DiI in human cells *in vitro*. In (**B-F**), mice were injected with 20 μg of eGFP mRNA-LNP-DiI. For (**B-G**), n = 6 mice/donors per time point from two independent experiments. In (**B**) and (**C**), a one-tailed paired Wilcoxon test was performed. In (**F**), an unpaired Mann-Whitney *U* test was performed with a two-stage Benjamini, Krieger, and Yekutieli FDR of 1% to correct for multiple comparisons.

To assess whether a direct uptake of LNP was associated with the capacity to instruct a pro-Tfh program in these cells, we developed an enhanced green fluorescent protein (eGFP) encoding mRNA encapsulated in LNP labeled with a fluorescent lipophilic dye DiI (Figure 6A). DiI signal allows for tracking LNP binding and/or uptake, whereas eGFP positivity provides a measure of mRNA-encoded protein expression. After the injection of mRNA-LNP, we observed an increased CD25 expression in the cDC2s that had bound/internalized LNP (DiI^+^), regardless of the mRNA translational status (eGFP^−^ and eGFP^+^) (Figure 6B and Figure S6A). Similarly, the LNP-driven induction of Cxcr5 was more prominent in DiI^+^ cDC2 populations compared to DiI^−^ cDC2s (Figure 6C), suggesting that the effect of the LNP is predominantly restricted to the cells that have bound/internalized LNP.

Next, we profiled cDC1, cDC2, LCs, plasmacytoid DCs (pDCs), macrophages, and inflammatory monocytes (Figure S6A) that are present in dLNs and capable of LNP uptake/expression of mRNA-encoded protein. We found that innate cell populations were capable of processing mRNA-LNP to varying degrees (Figure S6B), with cDCs being the most efficient at binding/internalizing LNP (DiI^+^) and translating the mRNA (eGFP^+^), and macrophages and pDCs having more modest processing capability. Interestingly, we reported a wave of DiI^+^eGFP^+^ monocytes infiltrating into dLNs 4 hours after immunization that was largely resolved by 24 hours (Figure S6B and C). We further refined the kinetics of LNP binding/internalization and mRNA-encoded protein expression by DCs 2-, 4-, 8-, 12-, and 24-hours after mRNA-LNP administration (Figure 6A and S6D). All DC subtypes were capable of binding/internalizing LNP and translating the mRNA to some extent (Figure 6D). LCs did not process mRNA-LNP as efficiently as cDCs. Of note, cDC2s were the fastest DC type that began binding/internalizing LNP by 2 hours and expressed mRNA-encoded protein by 4 hours post-immunization (Figure 6D–F). Despite the difference at early time points, cDC1s and cDC2s displayed equivalent percentages of DiI^+^eGFP^+^ cells 24 hours after mRNA-LNP injection. Even though cDC1 can efficiently internalize LNP/express eGFP and also upregulate CD25 (Figure 5C), they do not play a relevant role in Tfh cell differentiation in the setting of mRNA vaccination, as shown by the normal (if not more elevated) Tfh cell responses to Spikevax in cDC1-deficient *Batf3^−/−^* mice^49^ (Figure S6E).

To determine if human innate cells have a comparable ability to internalize mRNA-LNP, we incubated peripheral blood mononuclear cells (PBMCs) with fluorescent mRNA-LNP and analyzed LNP uptake by cDC1s and cDC2s after 4-, 8-, 16-, and 24-hours of *in vitro* culture. In line with our results in mice, both human cDC1s and cDC2s were able to bind/internalize mRNA-LNP within 24 hours (Figure 6G and S6F). It is worth mentioning that the ability of DCs from most healthy donors to express the mRNA-encoded protein *in vitro* was much lower than what was seen in mice *in vivo* (Figure S6G). Possible explanations for this discrepancy could be the variability in mRNA-LNP processing by different subjects, the increased toxicity of mRNA-LNP in an *in vitro* culture system, or the inability of fully differentiated human primary DCs to properly function *in vitro*.

In sum, our data underscore that cDC2 are the fastest and among the most efficient cells at processing mRNA-LNP *in vivo*.

### mRNA-LNP uptake by cDCs takes place predominantly in dLNs

**Figure 7.**
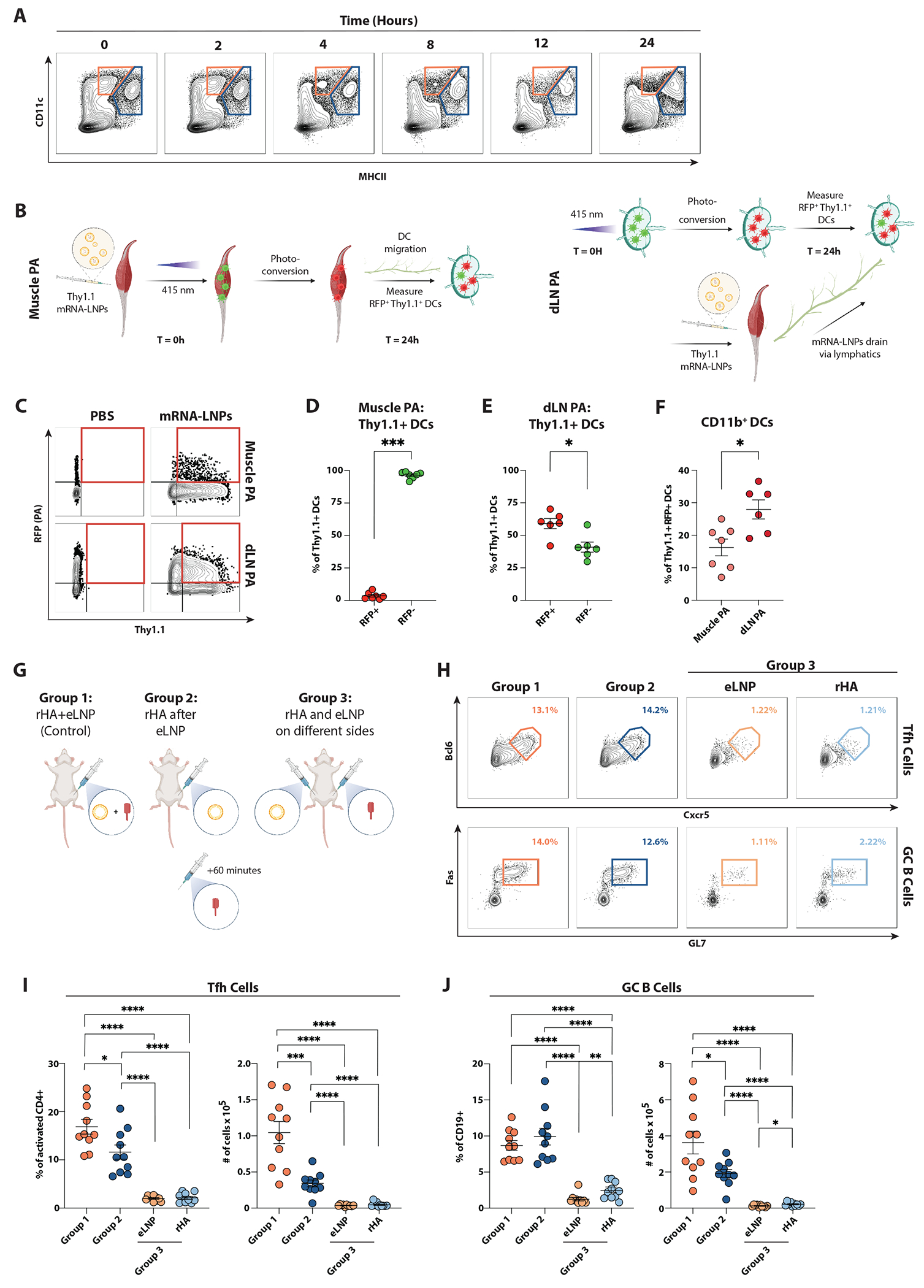
The uptake of mRNA-LNP by DCs occurs in draining lymph nodes. **(A)** Representative expression of MHCII and CD11c post-injection of eGFP mRNA-LNP-DiI. Cells are pre-gated as Live, TCRβ^−^CD19^−^. **(B)** Schematic of muscle (**Left**) and dLN (**Right**) photoactivation (PA) experiments. **(C)** Representative expression of RFP and Thy1.1 by DCs from dLNs of Muscle PA and dLN PA, 24 hours after injection. **(D and E)** Quantification of RFP^+^ and RFP^−^ Thy1.1^+^ DCs from **C**. PA cells are represented as red circles and non-PA cells as green circles. **(F)** Frequency of CD11b^+^ (cDC2) from **D** and **E**. **(G)** Experimental design of panels **H-J**. **(H)** Representative frequencies of Tfh and GC B cells from **G**. **(I-J)** Quantification of frequency and number of Tfh cells (**I**) and GC B cells (**J**) from **H**. Mice were injected with 20 μg of eGFP mRNA-LNP-DiI (**A**), 30 μg Thy1.1 mRNA-LNP (**B-F**), and 30 μg rHA (**H-J**). In Group 1, rHA and eLNP were mixed and injected at the same time; in Group 2, eLNP were injected one hour before rHA at the same site; and in Group 3, eLNP and rHA were injected at the same time but in opposite limbs. In all experiments, n = 6-10 mice per group from 2 independent experiments. In panels **D-F** and **I-J** unpaired two-tailed Mann-Whitney *U* test was performed.

Studies in rhesus macaques and mice have shown the rapid infiltration of various APC types into the injection site upon nanoparticle-based IM immunization^50–52^, suggesting a model where antigen uptake by migratory DCs in the muscle is followed by relocation of the DCs to the dLNs for subsequent antigen presentation^53,54^. To test this model, we asked if the mRNA-LNP-processing DCs in dLNs had a migratory phenotype. Migratory DCs can be characterized based on MHC-II expression level, with CD11c^+^MHC-II^hi^ cells being considered migratory and CD11c^+^MHC-II^int^ resident cells^37,55^. While this approach defines two distinct populations in steady-state LNs (Figure 7A), upon injection of mRNA-LNP, MHC-II and CD11c expression increased on dLN DCs, preventing the discrimination between resident and migratory cells.

To circumvent this issue, we utilized the Kikume Green-Red (KikGR) photoactivation (PA) system^56^ to label muscle DCs and track their migration to the dLN in conjunction with their mRNA-LNP processing capacity (Figure 7B). In KikGR mice, cells express GFP that is photoconverted into a red fluorescent protein (RFP) upon exposure to light. We determined the efficiency of PA upon light exposure of the muscle for 2 or 3 minutes. On average, 40% of DCs were RFP^+^ regardless of the duration of exposure to light (Figure S7A and B). Thus, the 2-minute interval of light exposure was used thereafter. Moreover, we found that the injection of mRNA-LNP promoted a rapid infiltration of CD45^+^ cells that did not express DC markers, resulting in decreased DC frequencies within 4 hours of injection (Figure S7C). Based on this finding, the muscle was photoactivated immediately following mRNA-LNP injection, when DC frequency was highest. KikGR mice were injected with a Thymus cell antigen 1.1 (Thy1.1) mRNA-LNP (Figure 7B and C), and the muscle was subsequently photoactivated near the injection site. After 24 hours, only ~3.6% of the Thy1.1^+^ DCs in the dLN were RFP^+^, whereas 96.4% of Thy1.1^+^ DCs were RFP^−^, indicating that a vast majority of mRNA-LNP-processing DCs were not in the muscle at the time of injection (Figure 7D). Additionally, the frequency of the Thy1.1^+^RFP^+^ DCs in dLNs did not significantly increase in comparison to PBS-injected mice (Figure S7D), emphasizing that these cells comprise a minority of DCs within the dLNs.

To further understand whether mRNA-LNP reaches dLNs through the afferent lymphatics and is internalized by DCs that were already in the dLN before the immunization, we performed additional experiments where we photoactivated the dLN before IM injection of mRNA-LNP (Figure 7B–C). Interestingly, we found that most Thy1.1^+^ DCs were RFP^+^ (Figures 7E and S7E), indicating that these DCs were present in dLNs at the time of immunization. Additionally, we found that roughly 15% of the Thy1.1^+^ RFP^+^ DCs were cDC2s when the muscle was photoactivated; however, this frequency doubled when photoactivating the dLNs (Figure 7F). The Thy1.1^+^ RFP^−^ DCs were likely DCs that were either not efficiently photoactivated or trafficked to the dLN.

In sum, the PA experiments suggested that most dLN cDC2 internalize mRNA-LNP *in situ* rather than migrating from the muscle upon mRNA-LNP uptake.

### LNP-adjuvanted responses occur in the dLNs

A high dose of custom-made mRNA-LNP was used throughout this study, raising the possibility that an excess of mRNA-LNP could result in systemic immune responses. To test this possibility, we conducted an experiment where one group of mice was immunized with rHA mixed with eLNP (Group 1); a second group of mice received eLNP first, then, an hour later, rHA on the ipsilateral side (Group 2); and a third group received rHA in one leg and eLNP in the contralateral leg at the same time (Group 3; Figure 7G). We reasoned that if the LNP act as a systemic adjuvant, group 3 should present GC responses in the LNs draining rHA. Importantly, we did not find detectable Tfh and GC B cell responses in the dLNs draining either injection site in Group 3, demonstrating that LNP do not act as systemic adjuvants when injected IM, even at high doses (Figure 7H–J). Interestingly, Group 2 data revealed that eLNP do not need to be co-formulated or physically coupled with rHA to be effective adjuvants, as the mice that received the rHA one hour after the eLNP presented robust (albeit slightly decreased) GC response.

Overall, our findings imply that, even at high doses, LNP exert their adjuvant activity locally in vaccine-dLNs.

## DISCUSSION

Nucleoside-modified mRNA-LNP vaccines have been instrumental in the fight against the SARS-CoV-2 pandemic, due in large part to their ability to drive potent GC responses, which ultimately result in the production of affinity-matured neutralizing antibodies^57^. A mechanistic understanding of how mRNA vaccines elicit robust GC responses, which is crucial for the rational design of mRNA vaccines with improved features, is still missing. In this study, we investigated the mechanisms of Tfh cell induction by mRNA vaccines and demonstrated that the nucleoside-modified mRNA and the LNP components possess adjuvant activity and cooperate to instruct an efficient GC program.

Immune cells express a multitude of PRRs for sensing exogenous RNA, making foreign RNA highly immunogenic^58^. Among these, the endosomal receptors TLR7/8 sense ssRNA, whereas TLR3 recognizes dsRNA along with the cytosolic sensors RIG-I and MDA5^59^, triggering in turn type I IFN production and upregulation of ISGs. Excess production of pro-inflammatory cytokines has negative consequences on the translational capacity of mRNA, resulting in hindered antigen production^17,60^. However, mRNA translation can be largely restored by combining nucleoside modification with cellulose purification of the *in vitro*-produced mRNA^17,60,61^. It was originally believed that nucleoside-modified and purified mRNA is completely immunosilent, as suggested by the lack of elevated production of pro-inflammatory cytokines *in vitro* and in circulation described in seminal studies from Karikó and colleagues^14,17^. However, it has not been determined whether the nucleoside-modified mRNA might still be sensed and result in a low, but biologically relevant production of type I IFNs that contribute to GC responses. In line with the importance of type I IFNs in mRNA vaccination, previous studies have reported the upregulation of ISGs in human peripheral mononuclear cells following the administration of the licensed nucleoside-modified mRNA-LNP vaccine BNT162b2^62^. Furthermore, a role for type I IFNs in the regulation of CD8 T cell responses to the BNT162b2 was reported in mice^63^. Herein, we demonstrate that type I IFNs are also key regulators of the induction of Tfh cells and GC responses to Spikevax, another licensed COVID-19 mRNA-LNP vaccine, as well as to our custom-made HA mRNA-LNP vaccine^4^. A major limitation of evaluating only the immune responses to mRNA-LNP vaccines is that it prevents us from distinguishing whether the type I IFN production results from the sensing of the mRNA or the LNP vaccine components. To overcome this major hurdle, we compared the immune responses in mice immunized with an mRNA-LNP or a recombinant protein-LNP vaccine. Our experimental approach revealed that, even if type I IFN can be driven by the LNP composition used in our study, IFN sensing by DCs is important for downstream GC reactions only in response to mRNA-containing vaccines, likely due to a more substantial role in endogenous antigen presentation in this setting. These data demonstrate that the mRNA component of the vaccine possesses inherent adjuvant activity, which is mediated, at least in part, by the production of type I IFNs. Of note, the adjuvant property of nucleoside-modified RNA is biologically relevant because it enables a superior generation of Tfh cells with IFN-γ and IL-21 production potential as well as a more efficient generation of antigen-specific memory B cells, bone marrow plasma cells, and neutralizing antibodies. It is reasonable to infer that the more abundant production of IL-21 driven by the mRNA is at least in part responsible for the superior B cell responses observed, as IL-21 has a pivotal role in the regulation of GC B cell and plasma cell responses^64^. Nonetheless, the higher IFN-γ production by Tfh cells could also impact B cell responses. Indeed, in the setting of influenza virus infection, IFN-γ produced by Tfh cells regulates the differentiation of lung-resident memory B cells, while being dispensable for GC B cell responses^65^. Additional and currently unappreciated qualitative differences in B cell helper signals provided by Tfh cells driven by the mRNA and LNP components might also be conducive to the superior B cell responses reported here and should be addressed in future studies.

The relevance of type I IFNs in response to mRNA sensing, along with our finding that the TLR adaptor protein MyD88 was required for GC responses to our custom mRNA vaccines^4^, led us to hypothesize that the nucleoside-modified mRNA could be sensed by TLR7/8. However, neither Spikevax nor our custom-made vaccine showed signs of TLR7 activation *in vitro* nor were required for Tfh cell differentiation *in vivo*. Similar findings were obtained for the other TLRs tested, prompting the question of whether MyD88 requirement for the GC responses to mRNA vaccines was related to IL-1 cytokines, which also signal via MyD88^27^. mRNA vaccines can drive the production of IL-1β, a cytokine typically produced downstream of inflammasome activation^66^. While LNP components, including specific ionizable lipids, have inherent IL-1-induction capacity, the presence of the nucleoside-modified mRNA enhances IL-1β production *in vitro*^66^. Of note, we found a significant reduction in Tfh cells when we blocked IL-1 signaling *in vivo*, highlighting the relevant role of IL-1 in GC responses to Spikevax and at least partially explaining the requirement for MyD88 in this setting. IL-1 has been extensively studied for its role in inflammation and the regulation of innate immunity, as well as its innate control of T cell responses^67^. Even though fewer studies have focused on the ability of IL-1 to regulate Tfh cells/antibody responses to vaccination, our data agree with evidence showing that certain vaccinations require IL-1 to elicit optimal Tfh cell and humoral responses^68–70^. Interestingly, IL-1 regulation of Tfh cell differentiation in mRNA vaccination did not rely on type I IFNs, in line with the notion that these are distinct types of inflammatory signals that can counteract each other^71^. Conversely, type I IFNs (and GCs) in response to mRNA vaccination depended on IRF3/IRF7, with the upstream sensor/s that remain to be identified. Our data suggest that a less characterized PRR or an underscribed mechanism of action is behind the induction of the IRF3/IRF7-type I IFN axis that is necessary for regulating GC responses to mRNA vaccines.

When comparing the transcriptional profile of DCs, our data revealed that the sensing of LNP was the major driver for the transcriptional changes observed across immunization conditions. An in-depth analysis of the LNP-driven transcriptional changes combined with the LIPSTIC system to track the antigen-presenting DCs highlighted selected clusters of biotin^lo^ cDC2s, whose transcriptomic profile was dictated by the sensing of the LNP rather than by their ability to engage T cells through CD40. eLNP were also associated with genes encoding for molecules important for DC migration, including *Cd81*^41^, *Cd274*^40^, *Cxcr5*^42^, and *Gpr183*^44,72–74^. The chemokine receptor Cxcr5 mediates the localization toward and within B cell follicles^73^ and is instrumental in DC localization and interaction with Cxcr5^+^ CD4 T cells at T-B borders^42^. We showed that LNP^+^ cDC2s have increased Cxcr5 expression, suggesting that direct sensing of eLNP is involved in Cxcr5 upregulation. Furthermore, we demonstrated that the LNP component promotes an Ebi2/7α, 25-HC axis by eliciting the conversion of cholesterol into 7α, 25-HC. At the steady state, Ebi2 dictates the positioning of CD4 T cells at the periphery of the LN paracortex^45^. It is believed that this spatial distribution facilitates antigen presentation by cDC2s, at least for antigens entering through the lymphatics, whose abundance exponentially decays with distance from the subcapsular sinus^23^. During immune responses, Ebi2 is further upregulated by activated CD4 T cells, favoring their localization farther in the periphery of T cell zones, at T-B borders^13,45^. Splenic cDC2s also upregulate Ebi2 upon activation to relocate at the T-B borders^44^, suggesting that in LNP-based vaccination, cDC2s that have sensed LNP can better localize at T-B borders, where they can provide pro-Tfh signals, including soluble CD25, to antigen-specific CD4 T cells and guide their fate commitment to Tfh cells. In line with this model, loss of Ebi2 in CD4 T cells can lead to a profound disruption in Tfh cell responses^13^, and in LNP-based vaccination, we found increased localization of CD25^+^ DCs at T-B borders, suboptimal GC responses in mice lacking CD25 expression in DCs, as well as a profound decrease in Tfh cell generation in C*h25h*^−/−^ mice that cannot produce the Ebi2 ligand 7α, 25-HC.

Overall, we have uncovered a dual mechanism of adjuvanticity driven by mRNA vaccines and generated mechanistic knowledge that will pave the way to a targeted manipulation of the mRNA and LNP vaccine components for more effective mRNA-LNP vaccines.

### Limitations of the study

Although our *in vitro* studies with reporter cell lines showed no direct activation of the PRR tested, it is worth noting that an *in vitro* reductionist approach may fail to accurately replicate events occurring *in vivo*. Hence, we cannot rule out that the signals that were investigated *in vitro* play a role in GC responses to vaccination *in vivo*, where multiple cellular types are present that could sense mRNA vaccines, their derivative products, or damage-associated molecular patterns lacking *in vitro*. While we tested all the PRRs listed in our work *in vivo* using individual knock-out mice, it is possible that compensatory effects might mask the importance of the sensors studied. This limitation cannot be easily addressed, as knock-out mice lacking all the PRRs studied here would take years to cross and would most likely be severely immunodeficient.

The ionizable lipid of our custom mRNA-LNP vaccine^4^ differs from those used in the licensed SARS-CoV-2 mRNA vaccines. Moderna utilizes the ionizable lipid SM-102 in the Spikevax^®^ vaccine, and Pfizer-BioNTech ALC-0315 in the Comirnaty^®^ vaccine. It has been demonstrated that the difference in these ionizable lipids leads to downstream changes in tissue delivery and antibody responses; however, it remains unknown whether the mechanisms of Tfh cell induction are affected by the specific ionizable lipid used in vaccine preparations^75^. Future studies will be needed to determine if, and how, the ionizable lipids and other components of LNP impact the magnitude and quality of Tfh cell generation. Moreover, while the mRNA in our custom vaccines contains the N1-methylpseudouridine modification, it can differ from the mRNA contained in the licensed COVID-19 vaccines for design and production process (e.g., different UTRs, length of poly(A) tail, codon optimization, purification approach, etc). These differences can potentially result in diverse immunostimulatory properties.

## RESOURCE AVAILABILITY

### Lead Contact

Further information and requests for resources and reagents should be directed to the lead contact, Michela Locci (michela.locci@pennmedicine.upenn.edu).

### Materials Availability

Reagents generated in this study are available upon request.

### Data and Code Availability

- The sequencing data generated in this paper have been deposited in GEO and are publicly available. The accession number is listed in the key resource table.
- New codes were not generated in this study.
- Additional information required to reanalyze the data reported here is available from the lead author upon request.

## STAR METHODS

### EXPERIMENTAL MODEL AND STUDY PARTICIPANT DETAILS

#### Mice.

C57BL6/J, *Cd11c-cre*-GFP (C57BL/6J-Tg(*Itgax-cre*,-EGFP)4097Ach/J), *Ifnar^flox/flox^* (B6(Cg)-*Ifnar1^tm1.1Ees^*/J), *Tlr2^−/−^* (B6.129-*Tlr2^tm1Kir^*/J), *Tlr3^−/−^* (B6;129S1-*Tlr3^tm1Flv^*/J), B6129SF2/J, *Tlr4^−/−^* (B6(Cg)-*Tlr4^tm1.2Karp^*/J), *Tlr7^−/−^* (B6.129S1-*Tlr7^tm1Flv^*/J), *Myd88^−/−^* (B6.129P2(SJL)-*Myd88^tm1.1Defr^*/J), *Mavs^−/−^* (B6;129-*Mavs^tm1Zjc^*/J), *Rigi^−/−^* (C57BL/6NJ-*Rigi^em1(IMPC)J^*/Mmjax), and STING GT (C57BL/6J-*Sting1^gt^*/J), *Il2ra^flox/flox^* (B6(129S4)-*Il2ra^tm1c(EUCOMM)Wtsi^*/TrmaJ), *Cd11c-cre* (B6.Cg.Tg(*Itgax*-*cre*)1-1Reiz/J) were purchased from The Jackson Laboratory. These last two strains were crossed to obtain *Cd11c-cre il2ra^flox/flox^* mice, and *Cd11c*-*cre* mice were used as controls. *Cd11c-cre Ifnar^flox/flox^* (IFNAR cKO) mice were generated by crossing *Cd11c-cre*-GFP and *Ifnar^flox/flox^*, and *Cd11c-cre*-GFP (*Cd11c-cre Ifnar^wt/wt^*) were used as controls. *Cd40^G5^* mice, in which *Cd40* contains a tag that consists of five N-terminal glycine residues (G5), and *Cd40lg^SrtA^* mice, which carry *Cd40lg* fused to the *Staphylococcus aureus* transpeptidase sortase A gene (*SrtA*), were previously described^33^. *Ch25h^−/−^* (B6.129S6-*Ch25h^tm1Rus^*/J) were purchased from The Jackson Laboratory and maintained as *Ch25h^−/+^* x *Ch25h^−/+^* breeding. *Ch25h^−/−^* and *Ch25h^+/+^* littermates were used as controls. *Irf3^−/−^Irf7^−/−^* (*Irf3/7−/−*) mice^76^ were provided by M. Diamond (Washington University) and T. Taniguchi (University of Tokyo) to J.J. Miner (University of Pennsylvania). CAG-KikGR-transgenic mice^56^, expressing a Kikume Green-Red photoconvertible fluorescent protein under the control of a CMV enhancer/chicken beta-actin promoter, were a gift from A. Hadjantonakis (Memorial Sloan Kettering Cancer Center) to G.D.Victora. CAG-KikGR transgenic mice were back-crossed to the C57BL6 background for at least 10 generations at Rockefeller University.

In all experiments, female and male mice were immunized between 6 – 10 weeks of age. Mice were housed in an Association for Accreditation of Laboratory Animal Care (AAALAC)-accredited facility, and studies were conducted under protocols approved by the University of Pennsylvania’s Institutional Animal Care and Use Committee (IACUC).

#### Human primary cells.

Cryopreserved peripheral blood mononuclear cells (PBMC) from deidentified healthy individuals were obtained from the Human Immunology Core at the University of Pennsylvania. Samples were collected from three male and three female individuals (aged 23-32 years).

#### Cell Lines.

HEK293-Dual cell lines with stable expression of human (h) hTLR3, hTLR4, hTLR7, hRIG-I, and hMDA-5 were purchased from InvivoGen (San Diego, CA, USA). These cells express secreted Lucia luciferase and embryonic alkaline phosphatase (SEAP) that are induced after IRF or NF-kB activation, respectively. Cells were handled and grown following the company’s protocols.

The day before the assay, the cells were seeded in 96-well culture-treated flat-bottom plates at a density of 6 × 10^^4^ cells/well. The culture media were replaced with prewarmed test medium [Dulbecco’s Modified Eagle Medium (DMEM, Corning), 4.5 g/l glucose, 2 mM L-glutamine, 10% fetal bovine serum (FBS, Gibco), and 1% Penicillin-Streptomycin (Pen-Strep, Gibco)] 1 hour before the treatments. Different amounts of Spikevax or HA mRNA-LNP were added per well, and 24 hours later, the readout was performed. IRF responses were quantified using 1X QUANTI-Luc 4 reagent (InvivoGen) and a luminometer (MiniLumat LB 9506, Berthold Technologies, Black Forest, CO, USA), following the manufacturer’s instructions. The data are presented as fold change in RLU (relative light unit) compared to culture conditions without treatment. Different positive controls were used according to the sensor tested: high molecular weight Poly(I:C) for hTLR3; low molecular weight Poly(I:C) for hRIG-I and hMDA-5 cell lines; LPS for hTLR4 cell line; Imiquimod and CL264 for hTLR7 cell line, all from InvivoGen.

### METHOD DETAILS

#### Production of nucleoside-modified mRNA.

Nucleoside-modified mRNA was produced as described previously^4^. Briefly, the coding sequences of the hemagglutinin (HA) of A/Puerto Rico/8/1934 (PR8), the receptor binding domain (RBD, amino acids 1-14 fused with amino acids 319-541) of SARS-CoV-2 spike (Wuhan-Hu-1, GenBank: MN908947.3), RBD fused via a SGGGG linker to the OTII binding region of ovalbumin (RBD-OVA, amino acids 318-340), extracellular domain of CD90.1 (Thy1.1, GenBank: AAR17087.1), EαGFP (Eα peptide: MEEFAKFASFEAQGALANIAVDKANLDVME; Spacer: DP; eGFP: MVSKGEELFTGVVPILVELDGDVNGHKFSVSGEGEGDATYGKLTLKFICTTGKLPVPWPTLVTTLTYGVQCFSRYPDHMKQHDFFKSAMPEGYVQERTIFFKDDGNYKTRAEVKFEGDTLVNRIELKGIDFKEDGNILGHKLEYNYNSHNVYIMADKQKNGIKVNFKIRHNIEDGSVQLADHYQQNTPIGDGPVLLPDNHYLSTQSALSKDPNEKRDHMVLLEFVTAAGITLGMDELYK), and enhanced green fluorescent protein (eGFP) were codon-optimized, synthesized and cloned into the mRNA production plasmid using AfeI-SpeI sites as previously described^77^. A T7-driven *in vitro* transcription reaction (Megascript, Ambion) was performed to generate mRNA with 101 nucleotide-long poly(A) tails. Capping of mRNA was performed in concert with transcription through the addition of a trinucleotide cap1 analog, CleanCap (TriLink), and m1Ψ-5’-triphosphate (TriLink) was incorporated into the reaction instead of UTP. Purification of mRNA was performed as described^78^, using a cellulose-based purification method. mRNAs were then assessed on an agarose gel before being stored at −20 °C.

The injectable suspension of COVID-19 vaccine, mRNA Spikevax^®^, 2024-2025 formula for individuals 12 years and older, manufactured by ModernaTX, Inc. (Princeton, NJ), was purchased from the Pharmacy at the Hospital of the University of Pennsylvania.

#### Lipid nanoparticle formulation of mRNA.

Purified mRNAs were encapsulated in LNP using a previously described self-assembly process^4^. Acuitas Therapeutics (Vancouver, Canada) generated the LNP and conducted the encapsulation of mRNA. Briefly, an ethanolic lipid mixture of an ionizable cationic lipid, 1,2-distearoyl-*sn*-glycero-3-phosphocholine, cholesterol, and a polyethylene glycol-lipid was rapidly mixed with an aqueous solution containing mRNA at acidic pH. The ionizable cationic lipid (pKa in the range of 6.0-6.5), proprietary to Acuitas Therapeutics (Vancouver, Canada), and LNP composition are described in the patent application WO 2017/004143. The average hydrodynamic diameter was ~80 nm with a polydispersity index of 0.02-0.06 as measured by dynamic light scattering using a Zetasizer Nano ZS (Malvern Instruments Ltd., Malvern, UK). The encapsulation efficiency was ~95% as determined using a Quant-iT Ribogreen assay (Life Technologies). DiI-labeled LNP were prepared by incorporating 1% of 1,1′-dioctadecyl-3,3,3′,3′- tetramethylindocarbocyanine perchlorate (DiIC18). The empty LNP (eLNP) used as an adjuvant with recombinant protein shared the identical lipid composition and formulation process as those used for the mRNA-LNP described above.

#### Production of recombinant proteins

##### RBD-OVA.

DNA sequences encoding the signal peptide (aa 1-14) and the RBD (aa 319-541) of SARS-CoV-2-Spike surface glycoprotein (NCBI Reference Sequence: YP_009724390) in fusion with an SGGGG linker with the OTII binding region of the chicken ovalbumin (OVA) precursor protein (aa 318-340; NCBI Reference Sequence: NP_990483.2) followed by a C-terminal hexahistidine-tag and a stop codon (hereafter referred to as RBD-OVA) was optimized to mammalian codon preference, produced by gene synthesis, and sub-cloned into the pCDNA3.1(−) mammalian expression plasmid by GenScript. Recombinant RBD-OVA was produced in Expi293F mammalian cells (Thermo Fisher Scientific, # A14527), and affinity-purified from cell culture supernatants by immobilized metal affinity chromatography. Protein production and purification were also conducted by GenScript. Sixteen milligrams of purified RBD-OVA were buffer-exchanged to phosphate-buffered saline (PBS) and concentrated to 1 mg/ml, filter sterilized, flash-frozen in liquid nitrogen in 300 μl aliquots, and stored at −80 °C. The integrity and purity of the protein were tested by reducing and non-reducing SDS-PAGE and western blotting techniques.

##### rHA.

Soluble and homotrimeric recombinant hemagglutinin (hereafter referred to as rHA) was synthesized in insect cells using a modified protocol previously detailed by^79^. Briefly, the DNA sequence encompassing the signal peptide (amino acids 1-17) and the ectodomain (amino acids 18-529) of the hemagglutinin of Influenza A virus (A/Puerto Rico/8/1934 (PR8), GenBank No: ADX99484.1) in fusion with the thrombin cleavage site (RS*LVPRGSP*), followed by the foldon trimerization domain of the T4 bacteriophage fibritin (*GSGYIPEAPRDGQAYVRKDGEWVLLSTFL*), according to Stevens *et al.*^80^ and a C-terminal hexahistidine-tag and stop codon was produced by gene synthesis (GenScript), and sub-cloned into the pFastBac-HTA vector. Bacmids were created in DH10Bac *E. coli* cells following the guidelines of the Bac-to-Bac Baculovirus Expression System manual. Recombinant baculovirus was then generated and produced in *Spodoptera frugiperda* Sf9 insect cells (Thermo Fisher Scientific, #12659017) using ExpiFectamine Sf transfection reagent (Thermo Fisher Scientific, #A38915) as per the manufacturer’s instructions. The cells were cultured at 27 °C in Sf-900 III serum-free medium in 6-well plates for 96 hours. The resulting cell culture supernatant, containing the P1 baculovirus rHA stock, was collected by centrifugation (2,000 *xg*, 10 minutes, 4 °C). Subsequently, 0.5 ml P1 stock was employed to infect Sf9 cells in 6-well plates with approximately 60-70 % confluency. The cell culture supernatant (P2 baculovirus rHA stock, approx. 4 ml) was collected 72 hours post-infection via centrifugation (2,000 *xg*, 10 minutes, 4 °C). For the next passage, 0.5 ml of P2 rHA baculovirus was used to infect Sf9 cells in five T175 flasks with approximately 60-70 % confluency. After 72 hours post-infection, the cell culture supernatant (P3 working baculovirus rHA stock, approx. 170 ml) was collected, centrifuged (2,000 *xg*, 10 minutes, 4°C), filtered, and stored at 4 °C until use. The rHA protein was expressed in *Trichoplusia ni* HighFive insect cells (Thermo Fisher Scientific, #B85502) cultured at 28 °C in HyClone SFX-Insect medium (Cytiva, # SH30350.03) as follows: HighFive cells from sixty confluent T175 flasks were harvested by tipping and collected by centrifugation (1200 *xg*, 28 °C, 10 minutes), then gently mixed with 150 ml of P3 baculovirus rHA working stock. The mixture was incubated at 28 °C for 15 minutes in the dark and slowly added to ten 1 l PETG tissue culture Erlenmeyer flasks (Nalgene, #4115-1000) containing pre-warmed 210 ml HyClone SFX-Insect medium each. The cells were grown at 28 °C in the dark on a shaker at 75 r.p.m. for 72 hours. The cell culture supernatant (2.2 l), containing the secreted homotrimeric rHA protein, was collected by centrifugation (2,000 *xg*, 4 °C, 10 minutes), supplemented with 75 ml Buffer E (50 mM sodium phosphate, pH 8.0, 250 mM NaCl, and 300 mM imidazole), and mixed. The mixture was filtered and subjected to affinity purification on four 5 ml HisTrap HP columns (Cytiva, #17524801) pre-equilibrated with buffer A (50 mM sodium phosphate, pH 8.0, 500 mM NaCl, and 20 mM imidazole) using an ÄKTA FPLC system. Each column was washed with 100 ml Buffer A, and proteins were step-eluted with 12 ml Buffer E per column. 70 mg of purified rHA were buffer-exchanged to phosphate-buffered saline (pH 7.4), concentrated to 1 mg/ml using Amicon Ultra centrifugal unit (Merck-Millipore, #UFC9030), filter-sterilized, flash-frozen in liquid nitrogen in 300 μl aliquots, and stored at −80 °C. The integrity and purity of the homotrimeric rHA were assessed using reducing and non-reducing SDS-PAGE and western blotting techniques.

##### rEαGFP.

Signal peptide: MGWSCIILFLVATATGVHS; Spacer: GS; Histidine tag: HHHHHH; Eα peptide: EEFAKFASFEAQGALANIAVDKANLDVME; Spacer: DP; eGFP: MVSKGEELFTGVVPILVELDGDVNGHKFSVSGEGEGDATYGKLTLKFICTTGKLPVPWPTLVTTLTYGVQCFSRYPDHMKQHDFFKSAMPEGYVQERTIFFKDDGNYKTRAEVKFEGDTLVNRIELKGIDFKEDGNILGHKLEYNYNSHNVYIMADKQKNGIKVNFKIRHNIEDGSVQLADHYQQNTPIGDGPVLLPDNHYLSTQSALSKDPNEKRDHMVLLEFVTAAGITLGMDELYK. GenScript conducted the codon optimization, gene synthesis, sub-cloning of the sequence into the vector pcDNA3.4, EcoRI/HindlIl, and transfection-grade plasmid expression in Turbo CHO cells. Purification was performed in one step using the His tag. The quality control of the total protein (302 aa) showed ≥ 90% purity tested by SDS-PAGE under non-reducing conditions and 92% with size exclusion-high performance liquid chromatography. The protein was stored in PBS, pH 7.2, at −80 °C.

#### Immunizations and Injections

##### Immunizations.

In all experiments, mice received a single immunization administered intramuscularly in the gastrocnemius muscle using a 0.5 ml 28 G x 1/2” insulin syringe (BD Biosciences, 329461). Unless otherwise stated, custom mRNA-LNP and recombinant proteins were administered at doses of 30 μg of antigen (mRNA or protein), and eLNP were administered as the volume equivalent to 30 μg of mRNA-LNP (typically equivalent to 30 μl at 30 μg/μl). Prior to immunizations, mRNA-LNP, LNP, and/or recombinant proteins were diluted in PBS to a final volume of 50 μl. Spikevax vaccine (0.1 μg/ml) was administered at doses of 3 – 5 μg in a final volume of 30 – 50 μl.

For the antigen presentation experiments, EαGFP-encoding mRNA-LNP (EαGFP mRNA-LNP) and recombinant EαGFP protein mixed with empty LNP (rEαGFP+eLNP) were administered at doses of 20 μg of antigen, along with the same dose of eLNP.

##### Antibody Treatments.

For blocking experiments, 500 μg of anti-IFNAR, 1 mg of anti-IL-1R, or 200 μg of anti-IL-18 blocking antibodies were delivered intraperitoneally (i.p.) 24 hours prior to immunization.

#### Sample Collection and Processing

##### LN Processing.

For DC-related analysis, draining LNs (inguinal and popliteal) were harvested at different post-immunization time points in DMEM + 10% FBS + 1X Glutamax (Gibco) + 1X Pen-Strep, disrupted with scissors, and then digested in RPMI1640 (Gibco) + 2% FBS + 20 mM HEPES (Gibco) + 400 U/ml type-IV collagenase (Gibco) for 30 minutes at 37 °C. LNs were then passed through a 21 G x 1 syringe (BD 309624) 5 times before being filtered through a 40 μm mesh filter. For lymphocyte analysis, LNs were harvested 7 days post-immunization and homogenized with a syringe plunger and filtered through a 40 μm cell strainer on ice to create a single-cell suspension.

##### Muscle Processing for flow cytometry.

The gastrocnemius muscle was harvested in DMEM + 10% FBS + 1X Glutamax + 1X Pen-Strep, disrupted with scissors, and then digested in RPMI + 7000 U Collagenase II (Worthington Biochemical) and 10% FBS for 1.5 hours at 37 °C. Samples were then pelleted at 400 *xg* for 5 minutes, and the supernatant was discarded. Pellets were resuspended in RPMI1640 + 500 U Collagenase II and 50 U Dispase (Sigma Aldrich) for 30 minutes at 37 °C. Samples were then passed through a 21 G x 1 syringe (BD 309624) 5 times before being filtered through a 40 μm mesh filter to create a single-cell suspension.

##### Spleen Processing.

Spleens were collected 70-77 days post-immunization, homogenized with a syringe plunger, and filtered through a 70 μm filter to generate a single-cell suspension. Red blood cells were lysed with ACK lysis buffer (Quality Biological) for 5-8 minutes on ice, and the reaction was stopped with cold PBS (Corning) or DMEM. Cells were resuspended in PBS or DMEM supplemented with 5% FBS + 1 mM EDTA (Invitrogen).

##### BM Processing.

BM was collected 70-77 days post-immunization and flushed from both femurs and tibias of each mouse using a 1 ml 25 G x 5/8” syringe (BD 309626). RBCs were lysed as described above.

##### Serum Processing.

Following collection at different post-immunization time points, blood was centrifuged at 14,000 *xg* (maximum speed) for 15-30 minutes, 4 °C. Serum was recovered and stored at −80 °C for ELISAs.

##### Tissue processing for cytokine analysis.

Draining LN and gastrocnemius muscle were collected in lysis buffer (750 μl M-PER protease lysis buffer containing protease inhibitor tablet cOmplete, from Thermo Scientific and Roche, respectively) on ice. One sterile 5 mm steel bead (Qiagen) was added per tube, and the samples were homogenized using the TissueLyser II (Qiagen), program #P2 at a frequency of 30 Hz. Four cycles, each of 1 minute, were performed for the muscle, or six cycles for the dLNs. The samples were rested for 30 seconds in between cycles. Then, tissues were incubated for 30 minutes on ice and centrifuged at 15,000 *xg* for 15 minutes. The supernatant was saved and immediately stored at −80 °C.

#### T cell intracellular cytokine assay.

This assay was performed as previously described^5^. Briefly, upon sample processing, CD4 T cells were isolated from dLN 7 days post-immunization using the Mojosort^™^ Mouse CD4 T cell isolation kit (Biolegend) according to the manufacturer’s instructions. Tfh cells were sorted from enriched CD4 T cells as Live, CD4^+^CD44^+^Cxcr5^+^ cells using a FACSAria Fusion (BD Biosciences) with a 70-μm nozzle. Sorted cells were incubated in IMDM (+ 10% FBS, + 1% Pen-Strep + 2 mM L-glutamine + 1 mM sodium pyruvate (Gibco) + 55 μM 2-mercaptoethanol (Gibco)) containing PMA (50 ng/ml) and ionomycin (1 μg/ml) (both from Sigma-Aldrich). After 2 hours, GolgiPlug (BD Biosciences) was added at 1:1,000, and the cells were incubated for 3 additional hours. Unstimulated cells were incubated with GolgiPlug without PMA and Ionomycin. After stimulation, cells were stained as described in the Flow Cytometry section.

#### Flow Cytometry.

All staining steps were performed at 4 °C in PBS + 2% FBS + 5 mM EDTA. Prior to staining, single-cell suspensions were incubated with anti-mouse CD16/CD32 blocking antibody at 1:1,000 (~7.6 μg/ml; BioXcell) for 20 minutes.

##### Tfh Cells.

Cells were incubated with biotinylated anti-Cxcr5 for 1 hour. Cells were washed and then incubated with a cocktail of fluorescently labeled anti-mouse mAbs, streptavidin, and Fixable Viability Dye e780 (Table S1) for 30 minutes. Cells were washed and then fixed and permeabilized in FoxP3/Transcription Factor staining buffer set (eBioscience), according to the manufacturer’s instructions, before intranuclear staining with anti-Bcl6. In all experiments, Tfh cells were analyzed 7 days post-immunization as Live, B220^−^CD4^+^CD44^+^CD62L^−^Cxcr5^+^Bcl6^+^ cells.

##### GC B Cells.

Cells were incubated with biotinylated anti-CD138 for 1 hour. Cells were washed and then incubated with a cocktail of fluorescently labeled anti-mouse mAbs, streptavidin, and Fixable Viability Dye e780 (Table S1) for 30 minutes. In all experiments, GC B cells were analyzed 7 days post-immunization as Live, CD19^+^CD3^−^Fas^+^GL7^+^ cells.

##### DCs, Memory B cells (MBC), and Human PBMCs.

Cells were washed and then incubated for 20-30 minutes with a cocktail of viability dye and the corresponding fluorescently labeled antibody cocktail in FACS buffer (PBS containing 2% FBS and 1 mM EDTA), (Table S1). In mice, total DCs were analyzed at different post-immunization time points as Live, CD3^−^CD19^−^ CD11c^+^MHCII^+^ cells; cDC1s as Live, CD3^−^CD19^−^CD11c^+^MHCII^+^CD24^+^EpCAM^−^ CD103^+^CD11b^−^ cells; cDC2s as Live, CD3^−^CD19^−^CD11c^+^MHCII^+^CD24^+^EpCAM^−^CD103^−^ CD11b^+^ cells; and LCs as Live, CD3^−^CD19^−^CD11c^+^MHCII^+^CD24^+^EpCAM^+^ cells. In humans, cDC1s were defined as Live, CD3^−^CD19^−^CD20^−^CD11c^+^HLA-DR^+^BDCA-3^+^BDCA1^−^ cells and cDC2s as Live, CD3^−^CD19^−^CD20^−^CD11c^+^HLA-DR^+^BDCA-3^−^BDCA1^+^cells. Memory B cells were analyzed 70-77 days post-immunization as Live, CD19^+^Fas^−^gM^−^IgD^−^CD38^+^CD27^−^, IgG1^+^ or IgG2c^+^ cells.

##### Antigen Presentation.

Cells were incubated with biotinylated Y-Ae antibody for 1 hour, washed, and then incubated with a cocktail of fluorescently labeled anti-mouse mAbs and streptavidin (Table S1) for 30 minutes. (Ag)-presenting DCs were defined as Live, TCRβ^−^CD19^−^MCH-II^+^CD11c^+^Y-Ae^+^ cells.

##### Intracellular staining for cytokines on unsorted lymphocytes.

Cells were stained with Fixable Viability Dye e780, and then fixed and permeabilized with Cytofix/Cytoperm (BD Biosciences) according to the manufacturer’s instructions. Cells were then resuspended with IL-21-Fc chimera protein (1:20) for 30 minutes, washed, and resuspended in anti-human IgG PE mAb for 20 minutes. Next, cells were washed and incubated overnight at 4 °C in a mixture of the remaining antibodies in permeabilization buffer (BD Biosciences; Table S1). The following day, the cells were washed prior to acquisition.

##### APCs.

Cells were incubated with biotinylated anti-Cxcr5 for 1 hour, washed, and then incubated with a cocktail of fluorescently labeled anti-mouse mAbs, streptavidin, and Fixable Viability Dye e780 (Table S1) for 30 minutes.

##### LIPSTC.

Lymphocytes from draining LNs were incubated for 30 minutes at 4 °C with a cocktail of surface marker antibodies (Table S1), and live, B220^−^CD3^−^NK1.1^−^MHCII^hi^CD11c^+^ DCs were sorted and used for single-cell transcriptomic studies.

All the unsorted samples were fixed with 1-4% paraformaldehyde (PFA) for 20-30 minutes and washed before their acquisition. Flow cytometry samples were acquired on a Cytek Aurora using SpectroFlow v2.2 (Cytek) or an LSR Fortessa (BD). Data were analyzed using FlowJo v.10.7.2 (BD).

#### Confocal Microcopy.

Inguinal LNs were harvested and fixed in 1% PFA for an hour at room temperature (RT). LNs were equilibrated in 30% sucrose overnight at RT. Samples were frozen in cryomolds using Optimal Cutting Temperature compound (OCT, Fisher Healthcare) on dry ice. 12 μm tissue sections were incubated for 10 minutes at RT with 0.1 M Glycine prior to staining. Sections were blocked with staining buffer (PBS, 5% Donkey Serum, and 0.05% Tween-20) containing an FcR blocking reagent. Sections were first stained with polyclonal anti-CD25 for 2 hours at RT, then stained with an anti-goat IgG secondary for 2 hours at RT, followed by staining with all directly conjugated antibodies outlined in Table S1 overnight at 4°C. Samples were washed with PBS between each staining step and after the conclusion of the final staining. Slides were mounted with ProLong^™^Diamond antifade (Invitrogen) and imaged using a Zeiss LSM980 confocal microscope at 20X magnification. Images were processed for publication using Fiji^81^.

#### LIPSTIC Labeling.

Upon sample processing, splenocytes from *CD40lg^SrtA/Y^*CD4-*cre* OT-II mice were incubated using a cocktail of biotinylated antibodies targeting Ter119, CD11c, CD25, B220, NK1.1, and CD8, followed by negative selection using anti-biotin beads (Miltenyi), as per the manufacturer’s instructions. 3 × 10^5^ *CD40lg^SrtA/Y^*CD4-*cre* OT-II CD4^+^ T cells were transferred into *Cd40^G5/G5^* recipient mice 18 hours prior to immunization. 22 hours post-immunization, Biotin-LPETG was injected i.p. (100 μl of 20 mM solution in PBS) six times, 20 minutes apart, and then inguinal and popliteal LN were harvested 20 minutes after the last injection. Single-cell suspensions were incubated with 1 μg/ml anti-CD16/32 (2.4G2, BioXCell) in PBS supplemented with 0.5% BSA and 2 mM EDTA (PBE) for 5 minutes at RT and then stained for cell surface markers at 4 °C for 20 minutes in PBS. For single-cell transcriptomic analysis, stained cells were further incubated with DNA-barcoded anti-biotin and sample hashtag (anti-MHC-I) antibodies (BioLegend) for 20 minutes in PBE, washed three times with PBE, and bulk-sorted.

#### Library preparation for single-cell RNA sequencing (scRNA-seq).

Sorted DCs were collected into a microfuge tube with 300 μl PBS supplemented with 0.4% BSA. After the sort, tubes were topped with PBS 0.4% BSA, centrifuged, and the buffer was carefully reduced by removing the volume with a pipette to a final volume of 40 μl. Cells were counted for viability and immediately submitted to library preparation. The scRNA-seq library was prepared using the 10X Single Cell Chromium system, according to the manufacturer’s instructions, at the Genomics Core of Rockefeller University, and was sequenced on an Illumina NovaSeq SP flowcell to a minimum sequencing depth of 30,000 reads per cell using read lengths of 26 bp read 1, 8bp i7 index, 98 bp read 2.

#### Analysis of scRNA-seq data.

scRNA-seq reads were processed with CellRanger v6.1.2. A custom reference genome was created by adding the RBD mRNA sequence to the GRCm38 mouse genome. Individual count tables were read into R and downstream analysis was performed using Seurat v4^82^. Sample demultiplexing was performed using the HTODemux function, and barcodes identified as negative or doublets were removed. Cells with at least 500 genes and <5% mitochondrial counts were considered for analysis, resulting in a total of 17,015 genes and 12,788 cells across the three treatments. The PCA plot was generated using DESeq2 from pseudo-bulk counts aggregated by individual animals. The biotin signal was normalized using the NormalizeData function (normalization.method = “CLR”, margin=1). Cells were classified as biotin-positive or -negative by using an elbow plot for ranking, where cells above the elbow (threshold set at 2.8) were considered positive and below were considered negative. GSEA was performed by contrasting pseudo-bulk counts of biotin-positive and -negative cells with DESeq2 and testing genes ranked by −log10 p-value (signed by log fold change) for enrichment of a previously published LIPSTIC signature^34^ with the R package fgsea. Single-cell transcriptomic counts were normalized using sctransform, while regressing out UMI counts and the percentage of mitochondrial counts.

Canonical correlation analysis (CCA) as implemented by Seurat v4 was used for treatment integration. Integrated data underwent principal component analysis (PCA), and graph-based clustering was then performed using the FindNeighbors and FindClusters functions, resulting in 13 clusters. Three clusters were identified as contaminants based on the expression of canonical marker genes: macrophages (*Mafb*), T cells (*Cd3d*), and B cells (*Cd79b*). These clusters were removed, and the remaining cells (n = 12,031) were re-clustered following the same workflow just described, yielding 14 clusters. Cluster markers were identified using the FindAllMarkers function. DC annotation was performed based on a curated list of marker genes, as shown in Figure 4E.

ISG (genes: *Ifit3b, Ifi213, Ifit2, Tnfsf10, Cmpk2, Cxcl10, Rsad2, Cd69, Gbp6*, and *Ifit1*) scores were computed using the AddModuleScore function of Seurat. Tfh (genes: *Cd80, Cd86, Cd274, Il2ra*, and *Gpr183*) and LIPSTIC (gene set from^34^) scores were computed using UCell^83^, with kNN smoothing.

#### Human *in vitro* experiments.

Cryopreserved PBMC samples were thawed and washed in complete RPMI (RPMI, 10% FBS, 1% Pen-Strep, 1% GlutaMAX). 2 × 10^^6^ cells/well were plated in a 48-well plate. eGFP-mRNA DiI-LNP were added at a final concentration of 10 μg/ml and incubated at 37 °C, 5% CO_2_ for the indicated time periods before flow cytometry staining and data acquisition.

#### KikGR Photoconversion.

Mice were anesthetized with isoflurane, shaved, and washed with ethanol and 0.005 M iodine solution. Immediately prior to immunization, photoactivation was performed by exposing either the gastrocnemius muscle or inguinal LN to 415 nm light for two minutes, unless otherwise indicated. The incision was then closed using FST autoclips. Flow cytometry analysis was conducted as described above, using the panel detailed in Table S1.

#### Transwell Assay.

This assay was performed as previously described^48^. Briefly, lipids from dLNs were extracted using the Folch method for lipid extraction^84^: dLNs were weighed and homogenized in serum-free medium containing 0.5% BSA at a concentration of 100 mg/ml. Total lipids were extracted using a chloroform: methanol (2:1, v/v) mixture. The samples were then filtered, mixed thoroughly, and kept on ice until phase separation occurred. The lower chloroform phase was collected, transferred to a new tube, and dried under a stream of nitrogen. Lyophilized lipid was dissolved at 100 mg/ml in ethanol. Lipid extracts were then diluted in 10 volumes of sterile chemotaxis medium (RPMI + 0.5% fatty acid-free BSA) and tested for GPR183-dependent bioactivity by seeding on transwell, 50:50 mixed, M12 B cell line transduced with an GPR183–IRES–GFP retroviral construct and mock M12 cells. The migration assay was performed at 37 °C for 3 hours, and cells were analyzed by flow cytometry. The migration of GPR183–GFP+ M12 cells over M12 cells (which indicates the relative concentration of the GPR183 ligand, 7α,25-HC) was normalized to the migration toward lipid-free migration medium and indicated in the text as ‘relative migration (a.u.)’. The purified 7α,25-HC was used as a positive control at a concentration of 100 nM.

#### qPCR.

Whole dLNs were extracted into RTL buffer and lysed using the Qiagen TissueLyzer^®^ II system. Tissues were disrupted using Qiagen 5 mm steel beads under the following conditions: 2X 30 Hz, 30 s per cycle. Total RNA was extracted using a Qiagen RNeasy kit following the manufacturer’s instructions. cDNA was then transcribed using Superscript II reverse transcriptase according to the manufacturer’s instructions. Quantitative real-time PCRs (qPCRs) for *Gapdh*, *Ch25h*, *Cyp7b1*, and *Hsd3b7* were performed using the following primer pairs: *Gapdh*: forward 5’GCACAGTCAAGGCCGAGAAT-3’ and reverse 5’-GCCTTCTCCATGGTGGTGAA-3’^85^; *Ch25h*: forward 5’-GCGACGCTACAAGATCCA-3’ and reverse 5’-CACGAACACCAGGTG CTG-3’; *Cyp7b*: forward 5’-TTCCTCCACTCATACACAATG-3’ and reverse 5’-CGTGCTTTTCTTCTTACCATC-3’; *Hsd3b7*: forward 5’-ACCATCCACAAAGTCAACG-3’ and reverse 5’-TCTTCATTGCCCCTGTAGA- 3’^46^. qPCRs were set up using SYBR Green PowerUp Master Mix (Applied Biosystems) and run on QuantStudio 6 PCR System. Relative mRNA levels were calculated by 2^−ΔCt^ according to the *Gapdh* gene abundance.

#### Enzyme-linked immunospot (ELISPOT).

MultiScreen HTS IP filter plates of 0.45 mm (Millipore, Sigma-Aldrich) were coated with 2.5 μg/ml rHA in bicarbonate buffer (35 mM NaHCO_3_ and 15 mM Na_2_CO_3_) overnight (ON) at 4 °C. Plates were washed 3X with PBS and blocked with complete DMEM (10% FBS + 1X Glutamax) for 2 hours at RT. Single-cell suspensions of BM cells were serially diluted in complete DMEM with halving dilutions starting at one million cells. Following ON incubation at 37 °C, 5% CO_2_ plates were washed 3X with wash buffer (0.05% Tween-20 in PBS). HRP-conjugated IgG was diluted 1:1,000, IgG1 and IgG2c were diluted 1:3,000 in complete DMEM and incubated for 2 hours at RT. Plates were washed 3X with wash buffer, and spots were developed using ELISPOT AEC Substrate Set (BD Bioscience). Membranes were dried ON and counted using a CTLImmunospot analyzer.

#### Enzyme-linked immunosorbent assay (ELISA)

Nunc Maxisorp flat-bottom 96-well plates (Thermo Scientific) were coated with 1 μg/ml rHA in bicarbonate buffer overnight (ON) at 4° C. Plates were washed 3X with wash buffer and blocked with blocking buffer (2% bovine serum albumin in PBS) for 1 hour at RT. Serum samples were serially diluted in blocking buffer, incubated for 2 hours at RT, and then washed 3X with wash buffer. HRP-conjugated IgG was diluted 1:1000, IgG1 and IgG2c were diluted 1:5,000 in blocking buffer and incubated for 1 hour, then washed 3X with wash buffer. Plates were developed with Pierce TMB Substrate (Thermo Scientific), and the reaction was stopped with 2 N sulfuric acid. Absorbance was measured at 450 nm using a Tecan Infinite 200Pro reader. Endpoint titers of serum samples were calculated using a consistent dilution of a control sample for each plate and reported as the inverse serum dilution calculated by nonlinear regression with an equivalent optical density to the control sample.

Type I IFN-α (all subtypes) and IFN-β levels were also determined with VeriKine-HS (High Sensitivity) Mouse ELISA kits (PBL Assay Science), following the manufacturer’s instructions.

#### Focus reduction neutralization test (FRNT).

In 96-well flat-bottom tissue culture plates, 2.5 × 10^^4^ MDCK-SIAT1 cells per well were seeded in Minimum Essential Media (MEM; Gibco) supplemented with 10% Fetal Bovine Serum (FBS; Millipore Sigma) the day before. Serum samples were first treated with receptor-destroying enzyme (RDE; Denka Seiken) with one volume of serum and three volumes of RDE. The mixture was incubated at 37 °C for two hours, and the enzyme was inactivated at 56 °C for 30 minutes. RDE-treated serum samples were serially diluted in serum-free MEM, and the virus was diluted to ~300 focus-forming units (FFU) per well, added to the serum samples, and incubated at RT for one hour. Cells were washed with serum-free MEM, and virus/antibody mixtures were transferred to the 96-well flat-bottom plate and incubated at 37 °C for one hour. Cells were washed once with serum-free MEM, and an overlay of 1.25% Avicel (IFF Pharma Solutions) in MEM supplemented with 0.2% gentamicin (Gibco) and 1% 1M HEPES (Gibco) was added to the cells. After 18 hours of incubation at 37 °C, the overlay was removed, and cells were fixed with 4% PFA for one hour at 4 °C in the dark. After removing PFA, 0.5% Triton X-100 in Dulbecco’s PBS (DPBS) was added to the cells for seven minutes. Triton was removed from wells, and 5% milk in DPBS was added for blocking for one hour at RT. Plates were washed with distilled water, and mouse anti-nucleoprotein antibody (clone IC5–1B7) diluted in 5% milk in DPBS was added to each well. After incubation for one hour at RT, plates were washed, and goat anti-mouse peroxidase-conjugated secondary antibody (Southern Biotech) diluted in 5% milk in DPBS was added to each well. Plates were incubated for one hour at RT, washed, and then TrueBlue peroxidase substrate (SeraCare) was added to the plates for one hour in the dark. Substrate was removed, and plates were scanned on the ImmunoSpot S6 plate reader. FRNT_50_ titers are reported as the highest reciprocal serum dilution that inhibited at least 50% of the virus in the virus-only control wells on each plate. FRNT assays were performed 70-77 days post-immunization.

### QUANTIFICATION AND STATISTICAL ANALYSIS

GraphPad Prism v10.1.1 was used to conduct all statistical analyses. Shapiro-Wilk and Kolmogorov-Smirnov tests were performed to establish the normal distribution of the data. The statistical analyses used are described in each figure legend and include the following calculations of statistical differences according to the distribution of the data: Two-way ANOVA with Tukey’s correction for multiple comparisons; Kruskal-Wallis test with Dunn’s correction for multiple comparisons; Unpaired two-tailed or one-tailed Mann-Whitney *U* test (where specified, to test a specific hypothesis), with or without a two-stage Benjamini, Krieger and Yekutieli FDR of 1% to correct for multiple comparisons; One-tailed paired Wilcoxon test. The precise number of samples analyzed in each graph is reported in the figure legends. Statistical significance was set at the critical values of p < 0.05 (*), p < 0.01 (**), p < 0.001 (***), and p < 0.0001 (****). Data were expressed as an average +/− standard error of the mean (SEM).

## Supplementary Material

1 Supplementary Figure 1. IFNAR expression on DCs regulates the magnitude of the GC responses to mRNA-LNP, related to Figure 1.
**(A)** Tfh cell (Live, B220^−^CD4^+^CD44^+^CD62L^−^Cxcr5^+^Bcl6^+^) gating strategy.
**(B)** GC B cell (Live, CD19^+^CD3^−^FAS^+^GL7^+^) gating strategy.
**(C)** Tfh cell (**Left**) and GC B cell (**Right**) frequency and absolute numbers were analyzed, as detailed in **A** and **B**, 7 days post-immunization with 30 μg HA mRNA-LNP.
**(D)** Tfh cell frequency (**Left**) and absolute numbers (**Right**) were analyzed, as detailed in **A**, 7 days post-immunization with 30 μg RBD mRNA-LNP.
**(E)** Kinetics of IFN-α (**Left**) and IFN-β (**Right**) levels in serum after immunization with 5 μg Spikevax.
**(F)** Kinetics of IFN-α (**Left**) and IFN-β (**Right**) levels in muscle (injection site) after immunization with 5 μg Spikevax.
**(G)** Tfh cell frequency (**Left**) and absolute (**Right**) numbers were analyzed, as detailed in **A**, 7 days post-immunization with 3 μg HA mRNA-LNP.
**(H)** Tfh cell and GC B cell frequency were analyzed in control (*Cd11c*-*cre*) or IFNAR cKO (*Cd11c*-*cre Ifnar^flox/flox^*) mice as detailed in **A** and **B**, 7 days post-immunization with HA mRNA-LNP (**Left**) or rHA+eLNP (**Right**).
**(I)** Quantification of CD80 expression in DCs from control or IFNAR cKO mice. **Left**, mice immunized with HA mRNA-LNP. **Right**, mice immunized with rHA+eLNP.
**(J)** Gating strategy of antigen-presenting DCs (Live, TCRβ^−^CD19^−^MHC-II^+^CD11c^+^eGFP^+^Y-Ae^+^).
In (**C and D**), mice received a single IM immunization with 30 μg of influenza virus hemagglutinin (HA) mRNA-LNP (**C**), or 30 μg of SARS-CoV-2 Spike receptor binding domain (RBD) mRNA-LNP (**D**); n = 8-12. In (**E and F**), mice received one IM injections with 5 μg of Spikevax; n = 6. In (**G**) mice received a single IM immunization with 3 μg of HA mRNA-LNP. In (**H and I**) mice received a single IM immunization with 30 μg of HA mRNA-LNP (**H**), or 30 μg rHA plus eLNP (rHA+eLNP) (**I**); n = 7-9. Data is compiled from 2-3 independent experiments. Statistical analysis: an unpaired two-tailed Mann-Whitney *U* test was performed. Error bars represent mean + SEM. *p ≤ 0.05, **p ≤ 0.01, ***p≤ 0.001, ****p ≤ 0.0001.

2 Supplementary Figure 2. GC responses to Spikevax do not require many common pattern recognition receptors, related to Figure 2.
**(A)** Frequencies of Tfh cells (**Left**), GC B cells (**Middle**), and antigen-specific (Spike, S+) GC B cells (**Right**) were analyzed in wild type (WT) and *Myd88−/−* mice, 7 days post-immunization with Spikevax.
**(B)** Human TLR7 (hTLR7) reporter cell line treated with different doses of Spikevax (**Left**), HA mRNA-LNP (**Right**), and the positive controls Imiquimod or CL264.
**(C)** Tfh cell (**Left**) and GC B cell (**Right**) frequency was analyzed in WT and *Tlr7^−/−^* mice, 7 days post-immunization with Spikevax.
**(D)** Tfh cell (**Left**) and GC B cell (**Right**) absolute numbers were analyzed in WT and *Tlr7^−/−^* mice, 7 days post-immunization with HA mRNA-LNP.
**(E)** Human TLR4 (hTLR4) reporter cell line treated with different doses of Spikevax (**Left**), HA mRNA-LNP (**Right**), and the positive control LPS.
**(F)** Tfh cell (**Left**) and GC B cell (**Right**) absolute numbers were analyzed in WT, *Tlr2^−/−^* and *Tlr4^−/−^* mice, 7 days post-immunization with Spikevax.
**(G)** Human TLR3 (hTLR3) reporter cell line treated with different doses of Spikevax and the positive control Poly (I:C).
**(H)** Tfh cell (**Left**) and GC B cell (**Right**) absolute numbers were analyzed in WT and *Tlr3^−/−^* mice,7 days post-immunization with Spikevax.
**(I)** Human TLR3 (hTLR3) reporter cell line treated with different doses of HA mRNA-LNP and the positive control Poly (I:C).
**(J)** Tfh cell (**Left**) and GC B cell (**Right**) absolute numbers were analyzed in WT and *Tlr3^−/−^* mice, 7 days post-immunization with HA mRNA-LNP.
**(K)** Human RIG-I (hRIG-I) reporter cell line treated with different doses of Spikevax and the positive control Poly (I:C).
**(L)** Tfh cell (**Left**) and GC B cell (**Right**) absolute numbers were analyzed in WT and *Rigi^−/−^* mice, 7 days post-immunization with Spikevax.
**(M)** Human MDA-5 (hMDA-5) reporter cell line treated with different doses of Spikevax and the positive control Poly (I:C).
**(N)** Tfh cell (**Left**) and GC B cell (**Right**) absolute numbers were analyzed in WT and *Mavs^−/−^* mice, 7 days post-immunization with Spikevax.
**(O)** Tfh cell (**Left**) and GC B cell (**Right**) absolute numbers were analyzed in WT and STING GT mice, 7 days post-immunization with Spikevax.
In (**A, C, F, H, L, N, and O**), mice received a single IM immunization with 3 μg of Spikevax. In (**D and J**), mice received a single IM immunization with 30 μg of HA mRNA-LNP. n = 6-11 mice per group. Data were combined from 2-3 independent experiments. Statistical analysis: an unpaired two-tailed Mann-Whitney *U* test was conducted. Error bars represent mean + SEM. *p ≤ 0.05, **p ≤ 0.01, ***p≤ 0.001, ****p ≤ 0.0001. In (**B, E, G, I, K, and M**) reporter cell lines were treated with different doses of Spikevax (**B, E, G, K, and M**) or HA mRNA-LNP (**B, E, and I**); HA mRNA-LNP; n = 4-17 replicates per condition. Data were combined from 2-3 independent experiments. Statistical analysis: Kruskal-Wallis was performed. Error bars represent mean + SEM. *p ≤ 0.05, **p ≤ 0.01, ***p≤ 0.001, ****p ≤ 0.0001.

3 Supplementary Figure 3. The nucleoside-modified mRNA regulates Tfh cell function and B cell responses, related to Figure 3.
**(A)** Representative flow cytometry data of HA-specific Tfh cells (I-A^b^ HA tetramer+) 7 days post-immunization.
**(B)** HA-specific Tfh cell frequency (**Left**) and absolute numbers (**Right**) from **A**.
**(C)** Gating strategy for sorting Tfh cells (Live, CD4^+^CD44^+^Cxcr5^+^) from purified CD4 T cells.
**(D)** Quantification of IL-4, IFN-γ, and IL-21 mean fluorescence intensity (MFI) on sorted Tfh cells. Tfh cells sorted from dLNs 7 days post-immunization were restimulated with PMA/Ionomycin.
**(E)** Quantification of IL-4+ and IFN-γ+ total CD4 T cells (**Left**) and unsorted Tfh cells (**Right**), 7 days post-immunization with RBD mRNA-LNP of control (*Cd11c-cre*^+^) or IFNAR cKO (*Cd11c-cre Ifnra^flox/flox^*) mice.
**(F)** Gating strategy of HA-specific memory B cell (MBC) populations.
**(G)** Representative flow cytometry data of HA-specific (HA+) IgG1+ (**Left**) and IgG2c (**Right**) MBCs, 70-77 days post-immunization with rHA+Luc mRNA-LNP or rHA+eLNP.
**(H)** Quantification of HA+ IgG1+ (**Left**) and HA+ IgG2c+ (**Right**) MBC frequency and absolute counts from **G**.
**(I)** Representative HA-specific (HA+) IgG1+ (**Left**) and IgG2c+ (**Right**) antibody-secreting cells (ASCs) measured by ELISPOT, 70-77 days post-immunization.
**(J)** Quantification of HA+ IgG1+ (**Left**) and HA+ IgG2c+ (**Right**) ASCs from **I**.
**(K)** Antigen-specific (HA+) total IgG1+ (**Left**) and IgG2c+ (**Right**) antibody levels post-immunization, calculated as endpoint titers.
In (**A-D, F-K**), mice received a single IM immunization with 30 μg of recombinant HA protein (rHA) mixed with 30 μg Luciferase (Luc) mRNA-LNP (rHA+Luc mRNA-LNP) or empty LNP (rHA+eLNP). n = 6-8 mice per group. In (**E)** mice received a single IM immunization with 30 μg of RBD mRNA-LNP. Statistical analysis: (**B, D, E, H, and J**), an unpaired two-tailed Mann-Whitney *U* test was conducted. (**K**) Two-way ANOVA was performed. In all cases, data were combined from 2-3 independent experiments. Error bars represent mean + SEM. **p ≤ 0.01, ***p ≤ 0.001, ****p ≤ 0.0001.

4 Supplementary Figure 4. LIPSTIC gene signature is enriched in biotin^+^ DCs, related to Figure 4.
**(A)** Amino acid sequence of the RBD-OVA construct, consisting of a signal peptide (black) to facilitate secretion (absent in the secreted protein), the RBD portion of SARS-CoV-2 Spike (red), a GS linker (blue), the peptide sequence of OVA recognized by OT-II (amino acids 318-340, green) and the hexahistidine-tag (purple) for affinity purification.
**(B)** Schematic of the RBD-OVA mRNA-LNP construct.
**(C)** UMAP clustering of sequencing data from Figure 4 before integration.
**(D)** Quantification of CD80 (**Left**) and CD86 (**Right**) mean fluorescence intensity (MFI) in DCs from mice immunized with rHA+eLNP or rHA+Luc mRNA-LNP.
**(E)** Feature plot of biotin expression before integration, displayed as a continuous variable.
**(F)** Heatmap displaying the top 10 differentially regulated genes in each DC group/cluster.
**(G)** Elbow plot of biotin-labeling, where the elbow represents the threshold (2.8) for biotin^+^ cells.
**(H)** GSEA analysis showing the enrichment of LIPSTIC-signature genes in biotin^+^ DCs.
**(I and J)** Proportion of *Cxcr5*-expressing cells in each group (**I**) and on the different DC clusters (**J**).
In **A-C, E-J**, Mice were immunized IM with either 30 μg of RBD-OVA mRNA-LNP, 30 μg recombinant RBD-OVA combined with empty LNP (rRBD-OVA+eLNP), or 30 μg recombinant RBD-OVA combined with AddaVax (rRBD-OVA+AddaVax); n = 2 mice per group. In **D**, mice received a single IM immunization with 30 μg recombinant HA (rHA)+Luc-mRNA-LNP or 30 μg rHA combined with eLNP (rHA+eLNP); n = 6-11 mice per group. Statistical analysis: (**D**) An unpaired two-tailed Mann-Whitney *U* test was conducted. Error bars represent mean + SEM. (**I**) The percentage of positive cells was calculated within each group based on single-cell expression data. *p ≤ 0.05, **p ≤ 0.01.

5 Supplementary Figure 5. LNP drive a pro-Tfh cell signature in cDC2s, related to Figure 5.
**(A)** Representative CD25 expression in cDC1s and cDC2s (as indicated) at baseline (0 hours, dark gray) versus 24 hours post-immunization (blue), determined by flow cytometry. Fluorescent intensity is displayed as count (normalized to mode).
**(B)** Confocal microscopy of dLN 24 hours post-immunization. Images from a representative sample are shown.
In **A**, mice received a single IM immunization containing 20 μg of fluorescent mRNA-LNP. In **B**, mice were IM immunized with 30 μg of HA mRNA-LNP.

6 Supplementary Figure 6. Conventional DCs are capable of mRNA-LNP uptake and expression, related to Figure 6.
**(A)** Gating strategy for analyzing APC in mice. Live, TCRβ^−^CD19^−^ were stratified into LCs (CD11c^+^MHCII^+^EpCAM^+^), cDC1s (CD11c^+^MHCII^+^EpCAM^−^CD103^+^CD11b^−^), cDC2s (CD11c^+^MHCII^+^EpCAM^−^CD103^−^CD11b^+^), pDCs (CD11b^−^PDCA1^+^), inflammatory monocytes (CD11b^+^PDCA1^−^Ly6G^−^SiglecF^−^Ly6C^+^), and macrophages (CD11b^+^PDCA1^−^Ly6G^−^SiglecF^−^Ly6C^−^).
**(B)** Quantification of LNP binding/uptake (DiI^+^) and mRNA-encoding protein expression (eGFP^+^) in the indicated cell populations, as defined in **A**. Each vertical bar represents 100% of the indicated cell type, and the color represents the portion of cells that are DiI^+^ eGFP^+^ (green), DiI^+^ eGFP^−^ (red), and DiI^−^ eGFP^−^ (blue). Error bars represent SEM.
**(C)** Quantification of cell types from **A**, represented as a percentage of live and lineage negative (Lin^−^) cells at the indicated time points.
**(D)** Complete gating strategy for mouse DCs, used to define DC populations in Figure 6B–F.
**(E)** Tfh cell (**Top**) and GC B cell (**Bottom**) frequency and absolute numbers were analyzed in wild type (WT) and *Batf3^−/−^* mice, 7 days post-immunization with 3 μg Spikevax.
**(F)** Gating strategy for human DCs, used to define DC populations in Figure 6G.
**(G)** Quantification of uptake and expression of eGFP mRNA-LNP-DiI in human DCs from Figure 6G, displayed as individual donors.
In (**A-D**), mice were injected with 20 μg of eGFP mRNA-LNP-DiI. In (**E**), mice were immunized with 3 μg Spikevax. For (**B and C**), n = 6 mice per time point. For (**E**), n = 6 mice per group. Statistical analysis: an unpaired two-tailed Mann-Whitney *U* test was conducted. Data is combined from two independent experiments. Error bars represent mean + SEM. *p ≤ 0.05, ***p ≤ 0.001.

7 Supplementary Figure 7. Photoactivation of muscle and lymph nodes reveals vaccine uptake within draining LNs, related to Figure 7.
**(A)** Gating strategy for defining DCs after photoactivation (PA).
**(B)** Efficacy of PA in the gastrocnemius muscle. Percentage of RFP^+^ DCs displayed as a percentage of total DCs (**Left**) or percentage of total CD45^+^ (**Right**) cells in the gastrocnemius immediately following PA.
**(C)** Quantification of CD45^+^ cell (**Left**) and DC (**Right**) frequencies in the gastrocnemius muscle at the indicated time points post-vaccination.
**(D-E)** Quantification of data from Figure 7D and E, displaying RFP^+^ and RFP^−^ Thy1.1^+^ DCs as frequency of total DCs from the dLNs of PBS (control) and Thy1.1 mRNA-LNP immunized mice upon muscle (**D**) or LN (**E**) PA.
In (**A and D-E**), mice were injected with 30 μg Thy1.1 mRNA-LNP. In (**C**), mice were injected with 20 μg of eGFP mRNA-LNP-DiI. In (**B**), n = 3-6 mice per group combined from 2 experiments. In (**C-E**), n = 5-6 mice per group combined from 2 experiments. Statistical analysis: In (**B**), an unpaired two-tailed Mann-Whitney *U* test was conducted. In panels (**C-E**), a Two-way ANOVA with Tukey’s correction for multiple comparisons was performed. Error bars represent mean + SEM. *p ≤ 0.05, **p ≤ 0.01, ***p ≤ 0.001, ****p ≤ 0.0001.

8 Supplementary Table 1. Flow cytometry and microscopy panels, related to Figures 1–7

## Figures and Tables

**Table:** Key resources table

REAGENT or RESOURCE Antibodies	SOURCE	IDENTIFIER
B220, clone RA3-6B2, BV650	BioLegend	Cat#103241
B220, clone RA3-6B2, BV785	BioLegend	Cat#103246
B220, clone RA3-6B2, AF700	BioLegend	Cat#103232
Bcl6, clone K112-91, AF647	Becton Dickinson (BD) Biosciences	Cat#561525
Biotin, Bio3-18E7, PE	Miltenyi Biotec	Cat#130-090-756
CD1c (BDCA1), clone L161, PE-Cy7	BioLegend	Cat#331515
CD3, clone 17A2, BV785	BioLegend	Cat#100232
CD3, clone 17A2, AF647	BioLegend	Cat#100209
CD3, clone SP34-2, AF700	BD Biosciences	Cat#561805
CD3e, clone 145-2c11, BUV395	BD Biosciences	Cat#563565
CD3e, clone 145-2c11, BV785	BioLegend	Cat#100355
CD4, clone RM4-5, PerCP-Cy5.5	BioLegend	Cat#100540
CD4, clone SK3, BUV 563	BD Biosciences	Cat#612912
CD8a, clone53-6.7, PerCP-Cy5.5	BioLegend	Cat#100734
CD8a, clone RPA-T8, PE-Cy5	BioLegend	Cat#301009
CD11b, clone M1/70, BV421	BioLegend	Cat#101236
CD11b, clone M1/70, FITC	BioLegend	Cat#101205
CD11b, clone M1/70, BV711	BioLegend	Cat#101242
CD11b, clone ICRF44, BV650	Biolegend	Cat#301336
CD11c, clone N418, PE-Cy7	BioLegend	Cat#117318
CD11c, clone N418, APC	BioLegend	Cat#117310
CD11c, clone N418, BUV496	BD Biosciences	Cat#750450
CD11c, clone B-LY6, BUV661	BD Biosciences	Cat#612967
CD14, clone 61D3, APCe780	Invitrogen	Cat#47-0149-42
CD16, clone 3G8, PerCP-Cy5.5	BioLegend	Cat#302027
CD16/CD32, clone 2.4G2, unlabeled	BioXCell	Cat#BE0307
CD19, clone 6D5, BV605	BioLegend	Cat#115540
CD19, clone 6D5, PE-Cy5	BioLegend	Cat#115510
CD19, clone HIB19, APC	BD Biosciences	Cat#555415
CD20, clone L27, BV605	BD Biosciences	Cat#740333
CD24, clone M1/69, BV605	BioLegend	Cat#101827
CD25, clone PC61, BUV395	BD Biosciences	Cat#564022
CD25, clone PC61, APC	BD Biosciences	Cat# 557192
CD25, polyclone, unlabeled	Invitrogen	Cat#PA5-46922
CD27, clone LG.3A10, PE-Dazzle	BioLegend	Cat#124228
CD38, clone 90, PE-Cy7	BioLegend	Cat#102717
CD40, clone 3/23, PE	BioLegend	Cat#124609
CD44, clone IM7, BV605	BioLegend	Cat#103047
CD44, clone IM7, FITC	BioLegend	Cat#103006
CD45, clone 30-F11, BUV805	BD Biosciences	Cat#748370
CD62L, clone MEL-14, BUV395	BD Biosciences	Cat#740218
CD80, clone 16-10A1, BV650	BioLegend	Cat#104732
CD80, clone 2D10.4, BUV395	BD Biosciences	Cat#751728
CD86, clone GL-1, BV421	BioLegend	Cat#105032
CD90.1 (Thy1.1), clone OX-7, BV421	Biolegend	Cat#202529
CD103, clone 2E7, BV785	BioLegend	Cat#121439
CD138, clone 281-2, Biotin	BD Biosciences	Cat#553713
CD141 (BDCA3), clone 1A4, BUV737	BD Biosciences	Cat#741867
CD200, clone OX-90, AF647	BioLegend	Cat#123816
CD303 (BDCA2/CLEC4C), clone 201A, BV 785	BioLegend	Cat#354221
CD326, clone G8.8, AF700	BioLegend	Cat#118240
CXCR5, clone SPRCL5, Biotin	eBioscience	Cat#13-7185-82
CXCR5, clone L138D7, BV421	BioLegend	Cat#145512
FAS, clone Jo2, BV510	BD Biosciences	Cat#563646
F4/80, clone T45-2342, BV605	BD Biosciences	Cat#743281
GL7, clone GL7, PerCP-Cy5.5	BioLegend	Cat#144610
HLA-DR, clone G46-6 (aka L243), BV711	BD Biosciences	Cat#563696
I-A/I-E, clone 2G9, BUV396	BD Biosciences	Cat#743876
IFNg, clone XMG1.2, BV650	BioLegend	Cat#505832
IFNAR-1, clone MAR1-5A3, unlabeled	BioXCell	Cat#BE0241
IgD, clone 11-26c.2a, PE-Cy7	BioLegend	Cat#405720
IgD, clone 11-26c.2a, BV421	Biolegend	Cat#405725
IgD, clone 11-26c.2a, BV650	BioLegend	Cat#405721
IgE, clone R35-72, BUV805	BD Biosciences	Cat#749151
IgG, HRP	Cell Signaling	Cat#7076
IgG Fc, polyclonal, PE	Invitrogen	Cat#12-4998-82
IgG H+L, polyclonal, AF488	Abcam	Cat#ab150141
IgG, polyclonal, unlabeled	BioXcell	Cat#BE0091
IgG1, clone SB77e, HRP	SouthernBiotech	Cat#1144-05
IgG1, clone M1 14D12, PE	Thermo-Scientific	Cat#12-4015-82
IgG1, clone MOPC-21, unlabeled	BioXcell	Cat#BE0083
IgG2b, clone R12-3, BUV563	BD Biosciences	Cat#749138
IgG2c, polyclonal, HRP	SouthernBiotech	Cat#1078-05
IgG2c, polyclonal, AF647	SouthernBiotech	Cat#1079-31
IgM, clone II/41, BB700	BD Biosciences	Cat#746099
IL-1R, clone JAMA-147	BioXCell	Cat#BE0256
IL-4, clone 11B11, AF647	BioLegend	Cat#504110
IL-17A, clone TC11-18H10.1, AF700	BioLegend	Cat#506914
IL-18, clone YIGIF74-1G7	BioXCell	Cat#BE0237
Influenza A nucleoprotein, clone IC5-1B7, unlabeled	This paper	N/A
Kappa, clone 187.1, HRP	SouthernBiotech	Cat#1170-05
Ly6C, clone HK1.4, PerCP-Cy5.5	Invitrogen	Cat#45-5932-82
Ly6G, clone 1A8, BV650	BioLegend	Cat#127641
MHCII, clone M5/114.15.2, BUV805	BD Biosciences	Cat#748844
MHCII, clone M5/114.15.2, BV421	BioLegend	Cat#107632
NK1.1, clone PK136, BV785	BioLegend	Cat#108749
PDCA1, clone 927, BV605	BioLegend	Cat#127025
PD-1, clone RMP1-30, PE	BioLegend	Cat#109104
PD-1, clone EH12.2H7, PE-Cy7	BioLegend	Cat#329918
PD-L1, clone 10F.9G2, BV711	BioLegend	Cat#124319
SiglecF, clone S17007L, APC	BioLegend	Cat#155508
SiglecF, clone S17007L, PE	BioLegend	Cay#155506
TCRb, clone H57-597, BV510	BioLegend	Cat#109234
TCRb, clone H57-597, APC-Fire 750	BioLegend	Cat#109246
Ter, clone-119, clone TER-119, APC-Fire 750	BioLegend	Cat#116250
Yae, clone eBioY-Ae, Biotin	Thermo-Scientific	Cat#13-5741-82
**Chemicals, peptides, and recombinant proteins**		
ACK Lysing buffer	Quality Biological	Cat#118-156-101
Avicel	IFF Pharma Solutions	N/A
CL264	InvivoGen	Cat#tlrl-c264e-5
Collagenase II	Worthington Biochemical	Cat#LS004177
cOmplete protease inhibitor cocktail tables	Roche	Cat#11697498001
Cytofix/Cytoperm	BD Biosciences	Cat#554655
Dispase	Sigma Aldrich	Cat#D4693-1G
EαGFP mRNA-LNP	This paper	N/A
ELISPOT AEC Substrate Kit	BD Bioscience	Cat#551951
Empty LNPs (eLNP)	Alameh et al.^4^	N/A
Enhanced green fluorescent protein (eGFP) mRNA-LNP-DiI	This paper	N/A
EZ-Link Micro Sulfo-NHS-Biotinylation Kit	Thermo Fisher	Cat#21925
Firefly luciferase (Luc) mRNA-LNP	Alameh et al.^4^	N/A
Fixable Viability Dye eFluor 780	BioLegend	Cat#65-0865-14
FoxP3/Transcription Factor Staining Buffer Set	eBioscience	Cat#00-5523-00
GolgiPlug protein transport	BD Biosciences	Cat#555029
Hemagglutinin (rHA) of Influenza A virus	This paper	N/A
High molecular weight Poly(I:C)	InvivoGen	Cat#tlrl-pic-5
I-Ab Influenza HA RSWSYIVETPNSENGIC Tetramer	NIH	N/A
IL-21R human IgG Fc chimera	R&D Systems	Cat#596-MR
Imiquimod	InvivoGen	Cat#tlrl-imqs-1
Ionomycin	Sigma-Aldrich	Cat#I0634-1MG
Lipopolysaccharide (LPS)	InvivoGen	Cat#L2630-10MG
Low molecular weight Poly(I:C)	InvivoGen	Cat#tlrl-picw
M-PER protease lysis buffer	Thermo Scientific	Cat#78501
Optimal Cutting Temperature compound (OCT)	Fisher Scientific	Cat#23-730-571
Phorbol 12-myristate 13-acetate (PMA)	Sigma-Aldrich	Cat#P1585-25MG
Pierce BCA protein assay kit	Thermo Scientific	Cat#23227
Pierce TMB Substrate	Thermo Scientific	Cat#34028
ProLong^™^Diamond antifade	Invitrogen	Cat#P36961
PR8 hemagglutinin (HA) mRNA-LNP	Alameh et al.^4^	N/A
rEαGFP	This paper, modified from Itano et al.^24^	N/A
RBD-OVA protein	This paper	N/A
SARS-CoV-2 receptor binding domain – chicken ovalbumin (RBD-OVA) mRNA-LNP	This paper	N/A
SARS-CoV-2 receptor binding domain (RBD) mRNA-LNP	Lederer et al.^5^	N/A
Spike-protein	GenScript	Cat#Z03481
Spike-protein	R&D Systems	Cat#10549-CV
SpikeVax COVID-19 mRNA vaccine (2024-2025 formulation)	Moderna	N/A
Streptavidin BUV661	BD Biosciences	Cat#612979
Streptavidin BV421	BioLegend	Cat#405225
Streptavidin BV650	BioLegend	Cat#405232
Streptavidin BV785	eBioscience	Cat#405249
Streptavidin AF647	BioLegend	Cat#405237
Streptavidin AF488	BioLegend	Cat#405235
Superscript II reverse transcriptase	Invitrogen	Cat#18064014
Thymus cell antigen 1.1 (Thy1.1) mRNA-LNP	This paper	N/A
TrueBlue peroxidase substrate	SeraCare	Cat#5510-0052
Zombie Aqua Fixable Viability Kit	Biolegend	Cat#423101
Zombie NIR Fixable Viability Kit	Biolegend	Cat#423105
**Critical commercial assays**		
IFN-α VeriKine-HS Mouse ELISA kits	PBL Assay Science	Cat#42115
IFN-β VeriKine-HS Mouse ELISA kits	PBL Assay Science	Cat#42410
**Deposited data**		
LIPSTIC RNA sequencing data	This paper	GSE26693
**Experimental models: Cell lines**		
Human MDA5 Dual Reporter HEK 293 Cells	InvivoGen	Cat#hkd-rna-mda5
Human TLR3 Dual Reporter HEK 293 Cells	InvivoGen	Cat#hkd-htlr3
Human TLR4 Dual Reporter HEK 293 Cells	InvivoGen	Cat#hkd-htlr4
Human TLR7 Dual Reporter HEK 293 Cells	InvivoGen	Cat#hkd-htlr7
Human RIG-I Dual Reporter HEK 293 Cells	InvivoGen	Cat#hkd-rna-rigi
**Biological samples**		
Human PBMC samples	University of Pennsylvania Human Immunology Core	https://www.med.upenn.edu/humanimmunologycore/
**Experimental models: Organisms/strains**		
C57BL6/J mice	The Jackson Laboratory	Cat#000664
CAG-KikGR mice	A. Hadjantonakis (Memorial Sloan Kettering Cancer Center)	N/A
*Cd11c-cre*-GFP mice: C57BL/6J-Tg(*Itgax-cre*,-EGFP)4097Ach/J	The Jackson Laboratory	Cat#007567
*Cd11c-cre* mice: B6.Cg.Tg(*Itgax-cre*)1-1Reiz/J	The Jackson Laboratory	Cat#008068
*Cd40^G5^* mice	G. Victora (The Rockefeller University)	N/A
*Cd40lg^SrtA^* mice	G. Victora (The Rockefeller University)	N/A
*Ch25h^−/−^* mice: B6.129S6-*Ch25h^tm1Rus^*/J	The Jackson Laboratory	Cat# 016263
*Ifnar^flox/flox^* mice: B6(Cg)-*Ifnar1^tm1.1Ees^*/J	The Jackson Laboratory	Cat#028256
*Il2ra^flox/flox^ mice*: B6(129S4)-*Il2ra^tm1c(EUCOMM)Wtsi^*/TrmaJ	The Jackson Laboratory	Cat#033093
*Irf3^−/−^Irf7^−/−^* mice	J.J. Miner (University of Pennsylvania)	N/A
*Myd88^−/−^* mice: B6.129P2(SJL)-*Myd88^tm1.1Defr^*/J	The Jackson Laboratory	Cat#009088
*Mavs^−/−^* mice: B6;129-*Mavs^tm1Zjc^*/J	The Jackson Laboratory	Cat#008634
*Rigi^−/−^* mice: C57BL/6NJ-*Rigi^em1(IMPC)J^*/Mmjax	The Jackson Laboratory	Cat#046070-JAX
STING GT mice: C57BL/6J-*Sting1^gt^*/J	The Jackson Laboratory	Cat#017537
*Tlr7^−/−^* mice: B6.129S1-*Tlr7^tm1Flv^*/J	The Jackson Laboratory	Cat#008380
**Oligonucleotides**		
*Ch25h*: forward GCGACGCTACAAGATCCA	Yi et al.^46^	N/A
*Ch25h*: reverse CACGAACACCAGGTG CTG	Yi et al.^46^	N/A
*Cyp7b*: forward TTCCTCCACTCATACACAATG	Yi et al.^46^	N/A
*Cyp7b*: reverse CGTGCTTTTCTTCTTACCATC	Yi et al.^46^	N/A
*Gapdh*: forward GCACAGTCAAGGCCGAGAAT	Panina et al.^85^	N/A
*Gapdh:* reverse GCCTTCTCCATGGTGGTGAA	Panina et al.^85^	N/A
*Hsd3b7*: forward ACCATCCACAAAGTCAACG	Yi et al.^46^	N/A
*Hsd3b7*: reverse TCTTCATTGCCCCTGTAGA	Yi et al.^46^	N/A
**Software and algorithms**		
BD FACSDiva Software v9	BD Bioscience	https://www.bdbiosciences.com/en-us/products/software/instrument-software/bd-facsdiva-software
FlowJo v10.7.2	BD Bioscience	https://www.bdbiosciences.com/en-us/products/software/flowjo-software?tab=flowJo-v11-software
GraphPad Prism v10.4.2	GraphPad Software	https://www.graphpad.com/features
10x Genomics Cell Ranger (v6.1.2)	10X Genomics	https://www.10xgenomics.com/support/software/cell-ranger/latest
R package DEseq2 (v1.40)	Love et al.^86^	https://genomebiology.biomedcentral.com/articles/10.1186/s13059-014-0550-8
R package Seurat (v4)	Hao et al.^82^	https://satijalab.org/seurat/articles/get_started.html
R package UCell (v2.4)	Andreatta et al.^83^	https://www.bioconductor.org/packages/release/bioc/html/UCell.html
**Other**		
Mojosort^TM^ Mouse CD4 T cell isolation kit	Biolegend	Cat#480033
Nunc Maxisorp flat-bottom 96-well plates	Thermo Scientific	Cat#442404
Qiagen RNeasy Micro kit	Qiagen	Cat#74004
SYBR Green PowerUp Master Mix	Applied Biosystems	Cat#A25742

## Highlights

- Induction of Tfh cells in mRNA vaccination depends on type I interferons acting on DCs.
- The mRNA vaccine component is immunostimulatory and shapes Tfh and B cell responses.
- Lipid nanoparticles (LNP) drive a Tfh cell-inducing transcriptional program in DC.
- mRNA-LNP uptake by DCs mostly occurs in draining lymph nodes.

## References

[1] Hogan MJ, and Pardi N (2021). mRNA Vaccines in the COVID-19 Pandemic and Beyond. Annu. Rev. Med 73, 17–39. 10.1146/annurev-med-042420-112725. 34669432

[2] Victora GD, and Nussenzweig MC (2022). Germinal Centers. Annu Rev Immunol 40, 413–442. 10.1146/annurev-immunol-120419-022408. 35113731

[3] Lederer K, Bettini E, Parvathaneni K, Painter MM, Agarwal D, Lundgreen KA, Weirick M, Muralidharan K, Castaño D, Goel RR, (2022). Germinal center responses to SARS-CoV-2 mRNA vaccines in healthy and immunocompromised individuals. Cell. 10.1016/j.cell.2022.01.027. PMC8808747 35202565

[4] Alameh M-G, Tombácz I, Bettini E, Lederer K, Sittplangkoon C, Wilmore JR, Gaudette BT, Soliman OY, Pine M, Hicks P, (2021). Lipid nanoparticles enhance the efficacy of mRNA and protein subunit vaccines by inducing robust T follicular helper cell and humoral responses. Immunity 54, 2877–2892.e7. 10.1016/j.immuni.2021.11.001. 34852217 PMC8566475

[5] Lederer K, Castaño D, Atria DG, Oguin TH, Wang S, Manzoni TB, Muramatsu H, Hogan MJ, Amanat F, Cherubin P, (2020). SARS-CoV-2 mRNA Vaccines Foster Potent Antigen-Specific Germinal Center Responses Associated with Neutralizing Antibody Generation. Immunity 53, 1281–1295.e5. 10.1016/j.immuni.2020.11.009. 33296685 PMC7680029

[6] Turner JS, O’Halloran JA, Kalaidina E, Kim W, Schmitz AJ, Zhou JQ, Lei T, Thapa M, Chen RE, Case JB, (2021). SARS-CoV-2 mRNA vaccines induce persistent human germinal centre responses. Nature 596, 109–113. 10.1038/s41586-021-03738-2. 34182569 PMC8935394

[7] Ma CS, Deenick EK, Batten M, and Tangye SG (2012). The origins, function, and regulation of T follicular helper cells. The Journal of experimental medicine 209, 1241–1253. 10.1084/jem.20120994. 22753927 PMC3405510

[8] Crotty S (2019). T Follicular Helper Cell Biology: A Decade of Discovery and Diseases. Immunity 50, 1132–1148. 10.1016/j.immuni.2019.04.011. 31117010 PMC6532429

[9] Vinuesa CG, Linterman MA, Yu D, and MacLennan ICM (2016). Follicular Helper T Cells. Annual review of immunology 34, 335–368. 10.1146/annurev-immunol-041015-055605. 26907215

[10] Crotty S (2014). T Follicular Helper Cell Differentiation, Function, and Roles in Di… - PubMed - NCBI. Immunity. 10.1016/j.immuni.2014.10.004. PMC4223692 25367570

[11] Ballesteros-Tato A, León B, Graf BA, Moquin A, Adams PS, Lund FE, and Randall TD (2012). Interleukin-2 inhibits germinal center formation by limiting T follicular helper cell differentiation. Immunity 36, 847–856. 10.1016/j.immuni.2012.02.012. 22464171 PMC3361521

[12] Johnston RJ, Choi YS, Diamond JA, Yang JA, and Crotty S (2012). STAT5 is a potent negative regulator of TFH cell differentiation. The Journal of experimental medicine 209, 243–250. 10.1084/jem.20111174. 22271576 PMC3281266

[13] Li J, Lu E, Yi T, and Cyster JG (2016). EBI2 augments Tfh cell fate by promoting interaction with IL-2-quenching dendritic cells. Nature 533, 110–114. 10.1038/nature17947. 27147029 PMC4883664

[14] Karikó K, Buckstein M, Ni H, and Weissman D (2005). Suppression of RNA Recognition by Toll-like Receptors: The Impact of Nucleoside Modification and the Evolutionary Origin of RNA. Immunity 23, 165–175. 10.1016/j.immuni.2005.06.008. 16111635

[15] Brown BD, Fauci AS, Belkaid Y, and Merad M (2023). RNA vaccines: A transformational advance. Immunity 56, 2665–2669. 10.1016/j.immuni.2023.11.009. 38091944

[16] Verbeke R, Hogan MJ, Loré K, and Pardi N (2022). Innate immune mechanisms of mRNA vaccines. Immunity 55, 1993–2005. 10.1016/j.immuni.2022.10.014. 36351374 PMC9641982

[17] Karikó K, Muramatsu H, Welsh FA, Ludwig J, Kato H, Akira S, and Weissman D (2008). Incorporation of Pseudouridine Into mRNA Yields Superior Nonimmunogenic Vector With Increased Translational Capacity and Biological Stability. Mol. Ther 16, 1833–1840. 10.1038/mt.2008.200. 18797453 PMC2775451

[18] Giovanni MD, Cutillo V, Giladi A, Sala E, Maganuco CG, Medaglia C, Lucia PD, Bono E, Cristofani C, Consolo E, (2020). Spatiotemporal regulation of type I interferon expression determines the antiviral polarization of CD4+ T cells. Nat Immunol 21, 321–330. 10.1038/s41590-020-0596-6. 32066949 PMC7043938

[19] Cucak H, Yrlid U, Reizis B, Kalinke U, and Johansson-Lindbom B (2009). Type I interferon signaling in dendritic cells stimulates the development of lymph-node-resident T follicular helper cells. Immunity 31, 491–501. 10.1016/j.immuni.2009.07.005. 19733096

[20] Bon AL, Schiavoni G, D’Agostino G, Gresser I, Belardelli F, and Tough DF (2001). Type i interferons potently enhance humoral immunity and can promote isotype switching by stimulating dendritic cells in vivo. Immunity 14, 461–470. 11336691 10.1016/s1074-7613(01)00126-1

[21] Dahlgren MW, Plumb AW, Niss K, Lahl K, Brunak S, and Johansson-Lindbom B (2022). Type I Interferons Promote Germinal Centers Through B Cell Intrinsic Signaling and Dendritic Cell Dependent Th1 and Tfh Cell Lineages. Front. Immunol 13, 932388. 10.3389/fimmu.2022.932388. 35911733 PMC9326081

[22] Cabeza-Cabrerizo M, Cardoso A, Minutti CM, Costa M.P. da, and Sousa C.R. e (2021). Dendritic Cells Revisited. Annu. Rev. Immunol 39, 131–166. 10.1146/annurev-immunol-061020-053707. 33481643

[23] Gerner MY, Casey KA, Kastenmuller W, and Germain RN (2017). Dendritic cell and antigen dispersal landscapes regulate T cell immunity. J. Exp. Med 214, 3105–3122. 10.1084/jem.20170335. 28847868 PMC5626399

[24] Itano AA, McSorley SJ, Reinhardt RL, Ehst BD, Ingulli E, Rudensky AY, and Jenkins MK (2003). Distinct Dendritic Cell Populations Sequentially Present Antigen to CD4 T Cells and Stimulate Different Aspects of Cell-Mediated Immunity. Immunity 19, 47–57. 10.1016/s1074-7613(03)00175-4. 12871638

[25] Kawai T, Ikegawa M, Ori D, and Akira S (2024). Decoding Toll-like receptors: Recent insights and perspectives in innate immunity. Immunity 57, 649–673. 10.1016/j.immuni.2024.03.004. 38599164

[26] Chaudhary N, Kasiewicz LN, Newby AN, Arral ML, Yerneni SS, Melamed JR, LoPresti ST, Fein KC, Petersen DMS, Kumar S, (2024). Amine headgroups in ionizable lipids drive immune responses to lipid nanoparticles by binding to the receptors TLR4 and CD1d. Nat. Biomed. Eng 8, 1483–1498. 10.1038/s41551-024-01256-w. 39363106 PMC11863198

[27] Dinarello CA (2018). Overview of the IL-1 family in innate inflammation and acquired immunity. Immunol. Rev 281, 8–27. 10.1111/imr.12621. 29247995 PMC5756628

[28] Rehwinkel J, and Gack MU (2020). RIG-I-like receptors: their regulation and roles in RNA sensing. Nat. Rev. Immunol 20, 537–551. 10.1038/s41577-020-0288-3. 32203325 PMC7094958

[29] Motwani M, Pesiridis S, and Fitzgerald KA (2019). DNA sensing by the cGAS–STING pathway in health and disease. Nat. Rev. Genet 20, 657–674. 10.1038/s41576-019-0151-1. 31358977

[30] Sauer J-D, Sotelo-Troha K, Moltke J. von, Monroe KM, Rae CS, Brubaker SW, Hyodo M, Hayakawa Y, Woodward JJ, Portnoy DA, (2010). The N-Ethyl-N-Nitrosourea-Induced Goldenticket Mouse Mutant Reveals an Essential Function of Sting in the In Vivo Interferon Response to Listeria monocytogenes and Cyclic Dinucleotides. Infect. Immun 79, 688–694. 10.1128/iai.00999-10. 21098106 PMC3028833

[31] Eisenbarth SC, Baumjohann D, Craft J, Fazilleau N, Ma CS, Tangye SG, Vinuesa CG, and Linterman MA (2021). CD4+ T cells that help B cells – a proposal for uniform nomenclature. Trends Immunol. 42, 658–669. 10.1016/j.it.2021.06.003. 34244056 PMC8324560

[32] Kuka M, Giovanni MD, and Iannacone M (2019). The role of type I interferons in CD4+ T cell differentiation. Immunol. Lett 215, 19–23. 10.1016/j.imlet.2019.01.013. 30771379 PMC7234836

[33] Pasqual G, Chudnovskiy A, Tas JMJ, Agudelo M, Schweitzer LD, Cui A, Hacohen N, and Victora GD (2018). Monitoring T cell–dendritic cell interactions in vivo by intercellular enzymatic labelling. Nature 553, 496–500. 10.1038/nature25442. 29342141 PMC5853129

[34] Chudnovskiy A, Castro TBR, Nakandakari-Higa S, Cui A, Lin C-H, Sade-Feldman M, Phillips BK, Pae J, Mesin L, Bortolatto J, (2024). Proximity-dependent labeling identifies dendritic cells that drive the tumor-specific CD4+ T cell response. Sci. Immunol 9, eadq8843. 10.1126/sciimmunol.adq8843. 39365874 PMC12419230

[35] Ridge JP, Rosa FD, and Matzinger P (1998). A conditioned dendritic cell can be a temporal bridge between a CD4+ T-helper and a T-killer cell. Nature 393, 474–478. 10.1038/30989. 9624003

[36] Schoenberger SP, Toes REM, van der Voort, E.I.H., Offringa R, and Melief CJM. (1998). T-cell help for cytotoxic T lymphocytes is mediated by CD40–CD40L interactions. Nature 393, 480–483. 10.1038/31002. 9624005

[37] Eisenbarth SC (2019). Dendritic cell subsets in T cell programming: location dictates function. Nat. Rev. Immunol 19, 89–103. 10.1038/s41577-018-0088-1. 30464294 PMC7755085

[38] Krishnaswamy JK, Gowthaman U, Zhang B, Mattsson J, Szeponik L, Liu D, Wu R, White T, Calabro S, Xu L, (2017). Migratory CD11b+ conventional dendritic cells induce T follicular helper cell–dependent antibody responses. Sci Immunol 2. 10.1126/sciimmunol.aam9169. PMC7847246 29196450

[39] Butler A, Hoffman P, Smibert P, Papalexi E, and Satija R (2018). Integrating single-cell transcriptomic data across different conditions, technologies, and species. Nature biotechnology 36, 411–420. 10.1038/nbt.4096. PMC6700744 29608179

[40] Lucas ED, Schafer JB, Matsuda J, Kraus M, Burchill MA, and Tamburini BAJ (2020). PD-L1 Reverse Signaling in Dermal Dendritic Cells Promotes Dendritic Cell Migration Required for Skin Immunity. Cell Rep. 33, 108258–108258. 10.1016/j.celrep.2020.108258. 33053342 PMC7688291

[41] Quast T, Eppler F, Semmling V, Schild C, Homsi Y, Levy S, Lang T, Kurts C, and Kolanus W (2011). CD81 is essential for the formation of membrane protrusions and regulates Rac1-activation in adhesion-dependent immune cell migration. Blood 118, 1818–1827. 10.1182/blood-2010-12-326595. 21677313

[42] León B, Ballesteros-Tato A, Browning JL, Dunn R, Randall TD, and Lund FE (2012). Regulation of TH2 development by CXCR5+ dendritic cells and lymphotoxin-expressing B cells. Nat. Immunol 13, 681–690. 10.1038/ni.2309. 22634865 PMC3548431

[43] Mostafavi S, Yoshida H, Moodley D, LeBoité H, Rothamel K, Raj T, Ye CJ, Chevrier N, Zhang S-Y, Feng T, (2016). Parsing the Interferon Transcriptional Network and Its Disease Associations. Cell 164, 564–578. 10.1016/j.cell.2015.12.032. 26824662 PMC4743492

[44] Lu E, Dang EV, McDonald JG, and Cyster JG (2017). Distinct oxysterol requirements for positioning naïve and activated dendritic cells in the spleen. Sci. Immunol 2. 10.1126/sciimmunol.aal5237. PMC5646419 28738017

[45] Baptista AP, Gola A, Huang Y, Milanez-Almeida P, Torabi-Parizi P, Urban JF, Shapiro VS, Gerner MY, and Germain RN (2019). The Chemoattractant Receptor Ebi2 Drives Intranodal Naive CD4+ T Cell Peripheralization to Promote Effective Adaptive Immunity. Immunity 50, 1188–1201.e6. 10.1016/j.immuni.2019.04.001. 31053504

[46] Yi T, Wang X, Kelly LM, An J, Xu Y, Sailer AW, Gustafsson J-A, Russell DW, and Cyster JG (2012). Oxysterol Gradient Generation by Lymphoid Stromal Cells Guides Activated B Cell Movement during Humoral Responses. Immunity 37, 535–548. 10.1016/j.immuni.2012.06.015. 22999953 PMC3465460

[47] Cyster JG, Dang EV, Reboldi A, and Yi T (2014). 25-Hydroxycholesterols in innate and adaptive immunity. Nat. Rev. Immunol 14, 731–743. 10.1038/nri3755. 25324126

[48] Ceglia S, Berthelette A, Howley K, Li Y, Mortzfeld B, Bhattarai SK, Yiew NKH, Xu Y, Brink R, Cyster JG, (2023). An epithelial cell-derived metabolite tunes immunoglobulin A secretion by gut-resident plasma cells. Nat. Immunol 24, 531–544. 10.1038/s41590-022-01413-w. 36658240 PMC10243503

[49] Hildner K, Edelson BT, Purtha WE, Diamond M, Matsushita H, Kohyama M, Calderon B, Schraml BU, Unanue ER, Diamond MS, (2008). Batf3 deficiency reveals a critical role for CD8alpha+ dendritic cells in cytotoxic T cell immunity. Sci. (N. York, NY) 322, 1097–1100. 10.1126/science.1164206. PMC2756611 19008445

[50] Liang F, Lindgren G, Lin A, Thompson EA, Ols S, Röhss J, John S, Hassett K, Yuzhakov O, Bahl K, (2017). Efficient Targeting and Activation of Antigen-Presenting Cells In Vivo after Modified mRNA Vaccine Administration in Rhesus Macaques. Mol Ther 25, 2635–2647. 10.1016/j.ymthe.2017.08.006. 28958578 PMC5768558

[51] Lindsay KE, Bhosle SM, Zurla C, Beyersdorf J, Rogers KA, Vanover D, Xiao P, Araínga M, Shirreff LM, Pitard B, (2019). Visualization of early events in mRNA vaccine delivery in non-human primates via PET–CT and near-infrared imaging. Nat. Biomed. Eng 3, 371–380. 10.1038/s41551-019-0378-3. 30936432

[52] Ols S, Yang L, Thompson EA, Pushparaj P, Tran K, Liang F, Lin A, Eriksson B, Hedestam GBK, Wyatt RT, (2020). Route of Vaccine Administration Alters Antigen Trafficking but Not Innate or Adaptive Immunity. Cell Rep. 30, 3964–3971.e7. 10.1016/j.celrep.2020.02.111. 32209459 PMC7198771

[53] Roth GA, Picece VCTM, Ou BS, Luo W, Pulendran B, and Appel EA (2022). Designing spatial and temporal control of vaccine responses. Nat. Rev. Mater 7, 174–195. 10.1038/s41578-021-00372-2. 34603749 PMC8477997

[54] Liang F, and Loré K (2016). Local innate immune responses in the vaccine adjuvant-injected muscle. Clin. Transl. Immunol 5, e74. 10.1038/cti.2016.19. PMC4855268 27195117

[55] Merad M, Sathe P, Helft J, Miller J, and Mortha A (2013). The Dendritic Cell Lineage: Ontogeny and Function of Dendritic Cells and Their Subsets in the Steady State and the Inflamed Setting. Annu. Rev. Immunol 31, 563–604. 10.1146/annurev-immunol-020711-074950. 23516985 PMC3853342

[56] Nowotschin S, and Hadjantonakis A-K (2009). Use of KikGR a photoconvertible green-to-red fluorescent protein for cell labeling and lineage analysis in ES cells and mouse embryos. BMC Dev. Biol 9, 49. 10.1186/1471-213x-9-49. 19740427 PMC2872819

[57] Bettini E, and Locci M (2021). SARS-CoV-2 mRNA Vaccines: Immunological Mechanism and Beyond. Vaccines (Basel) 9, 147. 10.3390/vaccines9020147. 33673048 PMC7918810

[58] Schlee M, and Hartmann G (2016). Discriminating self from non-self in nucleic acid sensing. Nat. Rev. Immunol 16, 566–580. 10.1038/nri.2016.78. 27455396 PMC7097691

[59] Brubaker SW, Bonham KS, Zanoni I, and Kagan JC (2014). Innate Immune Pattern Recognition: A Cell Biological Perspective. Annu. Rev. Immunol 33, 257–290. 10.1146/annurev-immunol-032414-112240. PMC5146691 25581309

[60] Karikó K, Muramatsu H, Ludwig J, and Weissman D (2011). Generating the optimal mRNA for therapy: HPLC purification eliminates immune activation and improves translation of nucleoside-modified, protein-encoding mRNA. Nucleic Acids Res. 39, e142–e142. 10.1093/nar/gkr695. 21890902 PMC3241667

[61] Pardi N, Hogan MJ, Porter FW, and Weissman D (2018). mRNA vaccines — a new era in vaccinology. Nat Rev Drug Discov 17, 261–279. 10.1038/nrd.2017.243. 29326426 PMC5906799

[62] Arunachalam PS, Scott MKD, Hagan T, Li C, Feng Y, Wimmers F, Grigoryan L, Trisal M, Edara VV, Lai L, (2021). Systems vaccinology of the BNT162b2 mRNA vaccine in humans. Nature 596, 410–416. 10.1038/s41586-021-03791-x. 34252919 PMC8761119

[63] Li C, Lee A, Grigoryan L, Arunachalam PS, Scott MKD, Trisal M, Wimmers F, Sanyal M, Weidenbacher PA, Feng Y, (2022). Mechanisms of innate and adaptive immunity to the Pfizer-BioNTech BNT162b2 vaccine. Nat Immunol, 543–555. 10.1038/s41590-022-01163-9. 35288714 PMC8989677

[64] Petersone L, and Walker LSK (2024). T-cell help in the germinal center: homing in on the role of IL-21. Int. Immunol 36, 89–98. 10.1093/intimm/dxad056. 38164992 PMC10880887

[65] Arroyo-Díaz NM, Bachus H, Papillion A, Randall TD, Akther J, Rosenberg AF, León B, and Ballesteros-Tato A (2023). Interferon-γ production by Tfh cells is required for CXCR3+ pre-memory B cell differentiation and subsequent lung-resident memory B cell responses. Immunity 56, 2358–2372.e5. 10.1016/j.immuni.2023.08.015. 37699392 PMC10592015

[66] Tahtinen S, Tong A-J, Himmels P, Oh J, Paler-Martinez A, Kim L, Wichner S, Oei Y, McCarron MJ, Freund EC, (2022). IL-1 and IL-1ra are key regulators of the inflammatory response to RNA vaccines. Nat Immunol, 23, 532–542. 10.1038/s41590-022-01160-y. 35332327

[67] Mantovani A, Dinarello CA, Molgora M, and Garlanda C (2019). Interleukin-1 and Related Cytokines in the Regulation of Inflammation and Immunity. Immunity 50, 778–795. 10.1016/j.immuni.2019.03.012. 30995499 PMC7174020

[68] Nakae S, Asano M, Horai R, Sakaguchi N, and Iwakura Y (2001). IL-1 Enhances T Cell-Dependent Antibody Production Through Induction of CD40 Ligand and OX40 on T Cells. J. Immunol 167, 90–97. 10.4049/jimmunol.167.1.90. 11418636

[69] Nakae S, Asano M, Horai R, and Iwakura Y (2001). Interleukin-1 beta, but not interleukin-1 alpha, is required for T-cell-dependent antibody production. Immunology 104, 402–409. 10.1046/j.1365-2567.2001.01337.x. 11899425 PMC1783318

[70] Ritvo P-GG, Churlaud G, Quiniou V, Florez L, Brimaud F, Fourcade G, Mariotti-Ferrandiz E, and Klatzmann D (2017). Tfr cells lack IL-2Rα but express decoy IL-1R2 and IL-1Ra and suppress the IL-1–dependent activation of Tfh cells. Sci. Immunol 2. 10.1126/sciimmunol.aan0368. 28887367

[71] Mayer-Barber KD, and Yan B (2017). Clash of the Cytokine Titans: counter-regulation of interleukin-1 and type I interferon-mediated inflammatory responses. Cell. Mol. Immunol 14, 22–35. 10.1038/cmi.2016.25. 27264686 PMC5214938

[72] Yi T, and Cyster JG (2013). EBI2-mediated bridging channel positioning supports splenic dendritic cell homeostasis and particulate antigen capture. eLife 2, e00757. 10.7554/elife.00757. 23682316 PMC3654440

[73] Qi H, Kastenmüller W, and Germain RN (2014). Spatiotemporal Basis of Innate and Adaptive Immunity in Secondary Lymphoid Tissue. Annu. Rev. Cell Dev. Biol 30, 141–167. 10.1146/annurev-cellbio-100913-013254. 25150013

[74] Gatto D, Wood K, Caminschi I, Murphy-Durland D, Schofield P, Christ D, Karupiah G, and Brink R (2013). The chemotactic receptor EBI2 regulates the homeostasis, localization and immunological function of splenic dendritic cells. Nat. Immunol 14, 446–453. 10.1038/ni.2555. 23502855

[75] Zhang L, More KR, Ojha A, Jackson CB, Quinlan BD, Li H, He W, Farzan M, Pardi N, and Choe H (2023). Effect of mRNA-LNP components of two globally-marketed COVID-19 vaccines on efficacy and stability. NPJ Vaccines 8, 156. 10.1038/s41541-023-00751-6. 37821446 PMC10567765

[76] Luksch H, Stinson WA, Platt DJ, Qian W, Kalugotla G, Miner CA, Bennion BG, Gerbaulet A, Rösen-Wolff A, and Miner JJ (2019). STING-associated lung disease in mice relies on T cells but not type I interferon. J. Allergy Clin. Immunol 144, 254–266.e8. 10.1016/j.jaci.2019.01.044. 30772497 PMC6612314

[77] Vadovics M, Zhao W, Daley EF, Lam K, Daly O, Rashid K, Lee HR, Schreiner P, Lundgreen KA, Gaudette BT, (2025). Tailoring the adjuvanticity of lipid nanoparticles by PEG lipid ratio and phospholipid modifications. Nat. Nanotechnol, 1312–1322. 10.1038/s41565-025-01958-5. 40550975 PMC13150451

[78] Vadovics M, Muramatsu H, Sárközy A, and Pardi N (2024). Production and Evaluation of Nucleoside-Modified mRNA Vaccines for Infectious Diseases. Methods Mol. Biol. (Clifton, NJ) 2786, 167–181. 10.1007/978-1-0716-3770-8_7. 38814394

[79] Margine I, Palese P, and Krammer F (2013). Expression of Functional Recombinant Hemagglutinin and Neuraminidase Proteins from the Novel H7N9 Influenza Virus Using the Baculovirus Expression System. J Vis Exp, e51112. 10.3791/51112. 24300384 PMC3970794

[80] Stevens J, Corper AL, Basler CF, Taubenberger JK, Palese P, and Wilson IA(2004). Structure of the Uncleaved Human H1 Hemagglutinin from the Extinct 1918 Influenza Virus. Science 303, 1866–1870. 10.1126/science.1093373. 14764887

[81] Schindelin J, Arganda-Carreras I, Frise E, Kaynig V, Longair M, Pietzsch T, Preibisch S, Rueden C, Saalfeld S, Schmid B, (2012). Fiji: an open-source platform for biological-image analysis. Nat. Methods 9, 676–682. 10.1038/nmeth.2019. 22743772 PMC3855844

[82] Hao Y, Hao S, Andersen-Nissen E, Mauck WM, Zheng S, Butler A, Lee MJ, Wilk AJ, Darby C, Zager M, (2021). Integrated analysis of multimodal single-cell data. Cell 184, 3573–3587.e29. 10.1016/j.cell.2021.04.048. 34062119 PMC8238499

[83] Andreatta M, and Carmona SJ (2021). UCell: Robust and scalable single-cell gene signature scoring. Comput. Struct. Biotechnol. J 19, 3796–3798. 10.1016/j.csbj.2021.06.043. 34285779 PMC8271111

[84] Folch J, Lees M, and Stanley GHS (1957). A SIMPLE METHOD FOR THE ISOLATION AND PURIFICATION OF TOTAL LIPIDES FROM ANIMAL TISSUES. J. Biol. Chem 226, 497–509. 10.1016/s0021-9258(18)64849-5. 13428781

[85] Panina Y, Germond A, Masui S, and Watanabe TM (2018). Validation of Common Housekeeping Genes as Reference for qPCR Gene Expression Analysis During iPS Reprogramming Process. Sci. Rep 8, 8716. 10.1038/s41598-018-26707-8. 29880849 PMC5992140

[86] Love MI, Huber W, and Anders S (2014). Moderated estimation of fold change and dispersion for RNA-seq data with DESeq2. Genome biology 15, 550. 10.1186/s13059-014-0550-8. 25516281 PMC4302049

