# Supplementary figures and images for: Distinct components of mRNA vaccines cooperate to instruct efficient germinal center responses
Source: Cell. Author manuscript; Available in PMC 2026 Feb 6. (PMC12878702; doi:10.1016/j.cell.2025.11.023)

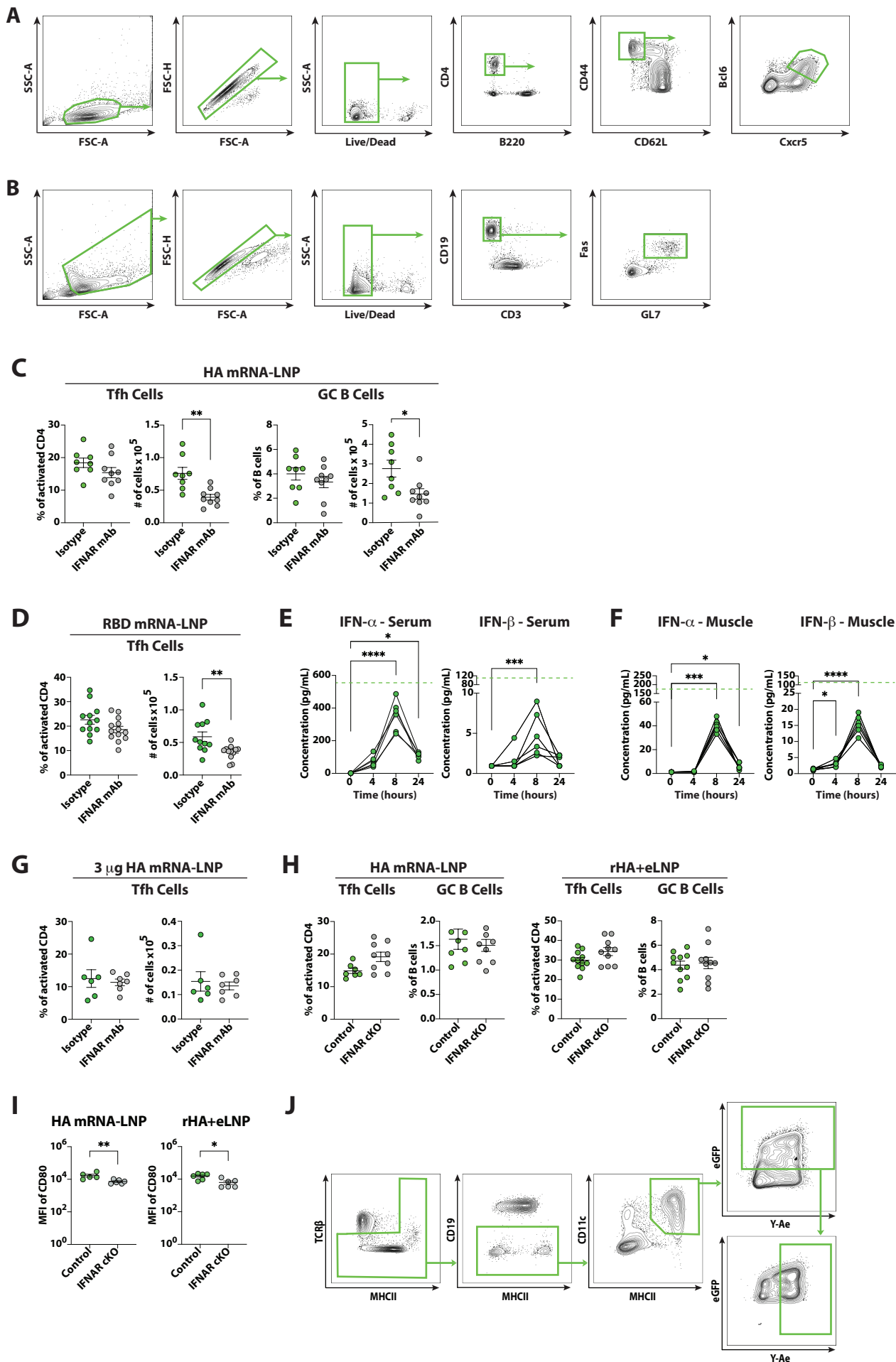

Supplement: 1 — Supplementary Figure 1. IFNAR expression on DCs regulates the magnitude of the GC responses to mRNA-LNP, related to Figure 1. (A) Tfh cell (Live, B220−CD4+CD44+CD62L−Cxcr5+Bcl6+) gating strategy. (B) GC B cell (Live, CD19+CD3−FAS+GL7+) gating strategy. (C) Tfh cell (Left) and GC B cell (Right) frequency and absolute numbers were analyzed, as detailed in A and B, 7 days post-immunization with 30 μg HA mRNA-LNP. (D) Tfh cell frequency (Left) and absolute numbers (Right) were analyzed, as detailed in A, 7 days post-immunization with 30 μg RBD mRNA-LNP. (E) Kinetics of IFN-α (Left) and IFN-β (Right) levels in serum after immunization with 5 μg Spikevax. (F) Kinetics of IFN-α (Left) and IFN-β (Right) levels in muscle (injection site) after immunization with 5 μg Spikevax. (G) Tfh cell frequency (Left) and absolute (Right) numbers were analyzed, as detailed in A, 7 days post-immunization with 3 μg HA mRNA-LNP. (H) Tfh cell and GC B cell frequency were analyzed in control (Cd11c-cre) or IFNAR cKO (Cd11c-cre Ifnarflox/flox) mice as detailed in A and B, 7 days post-immunization with HA mRNA-LNP (Left) or rHA+eLNP (Right). (I) Quantification of CD80 expression in DCs from control or IFNAR cKO mice. Left, mice immunized with HA mRNA-LNP. Right, mice immunized with rHA+eLNP. (J) Gating strategy of antigen-presenting DCs (Live, TCRβ−CD19−MHC-II+CD11c+eGFP+Y-Ae+). In (C and D), mice received a single IM immunization with 30 μg of influenza virus hemagglutinin (HA) mRNA-LNP (C), or 30 μg of SARS-CoV-2 Spike receptor binding domain (RBD) mRNA-LNP (D); n = 8-12. In (E and F), mice received one IM injections with 5 μg of Spikevax; n = 6. In (G) mice received a single IM immunization with 3 μg of HA mRNA-LNP. In (H and I) mice received a single IM immunization with 30 μg of HA mRNA-LNP (H), or 30 μg rHA plus eLNP (rHA+eLNP) (I); n = 7-9. Data is compiled from 2-3 independent experiments. Statistical analysis: an unpaired two-tailed Mann-Whitney U test was performed. Error bars represe [file NIHMS2129750-supplement-3.pdf]

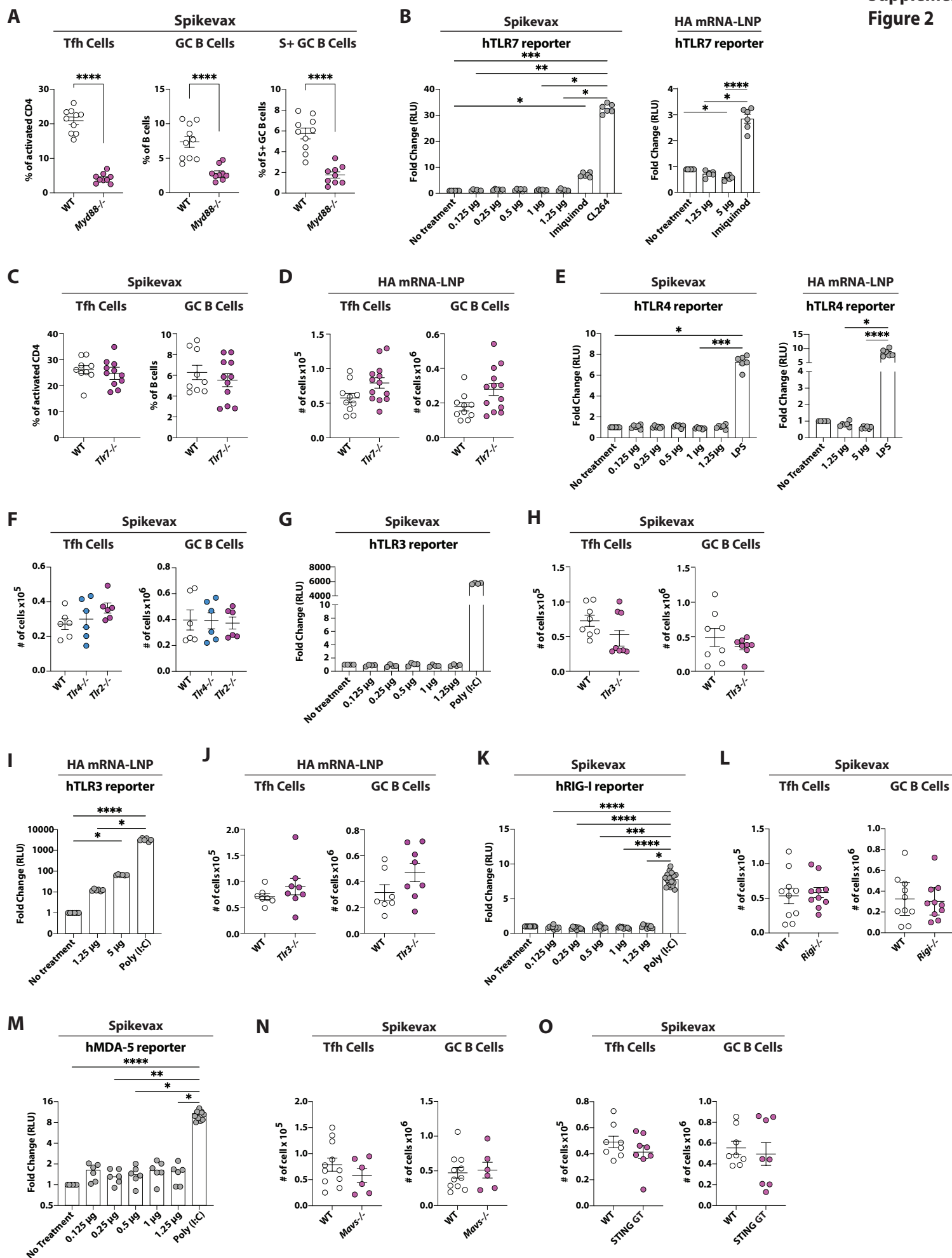

Supplement: 2 — Supplementary Figure 2. GC responses to Spikevax do not require many common pattern recognition receptors, related to Figure 2. (A) Frequencies of Tfh cells (Left), GC B cells (Middle), and antigen-specific (Spike, S+) GC B cells (Right) were analyzed in wild type (WT) and Myd88−/− mice, 7 days post-immunization with Spikevax. (B) Human TLR7 (hTLR7) reporter cell line treated with different doses of Spikevax (Left), HA mRNA-LNP (Right), and the positive controls Imiquimod or CL264. (C) Tfh cell (Left) and GC B cell (Right) frequency was analyzed in WT and Tlr7−/− mice, 7 days post-immunization with Spikevax. (D) Tfh cell (Left) and GC B cell (Right) absolute numbers were analyzed in WT and Tlr7−/− mice, 7 days post-immunization with HA mRNA-LNP. (E) Human TLR4 (hTLR4) reporter cell line treated with different doses of Spikevax (Left), HA mRNA-LNP (Right), and the positive control LPS. (F) Tfh cell (Left) and GC B cell (Right) absolute numbers were analyzed in WT, Tlr2−/− and Tlr4−/− mice, 7 days post-immunization with Spikevax. (G) Human TLR3 (hTLR3) reporter cell line treated with different doses of Spikevax and the positive control Poly (I:C). (H) Tfh cell (Left) and GC B cell (Right) absolute numbers were analyzed in WT and Tlr3−/− mice,7 days post-immunization with Spikevax. (I) Human TLR3 (hTLR3) reporter cell line treated with different doses of HA mRNA-LNP and the positive control Poly (I:C). (J) Tfh cell (Left) and GC B cell (Right) absolute numbers were analyzed in WT and Tlr3−/− mice, 7 days post-immunization with HA mRNA-LNP. (K) Human RIG-I (hRIG-I) reporter cell line treated with different doses of Spikevax and the positive control Poly (I:C). (L) Tfh cell (Left) and GC B cell (Right) absolute numbers were analyzed in WT and Rigi−/− mice, 7 days post-immunization with Spikevax. (M) Human MDA-5 (hMDA-5) reporter cell line treated with different doses of Spikevax and the positive control Poly (I:C). (N) Tfh cell (Left) and GC B cell (Right) absolute numbe [file NIHMS2129750-supplement-1.pdf]

# Supplementary Figure 3

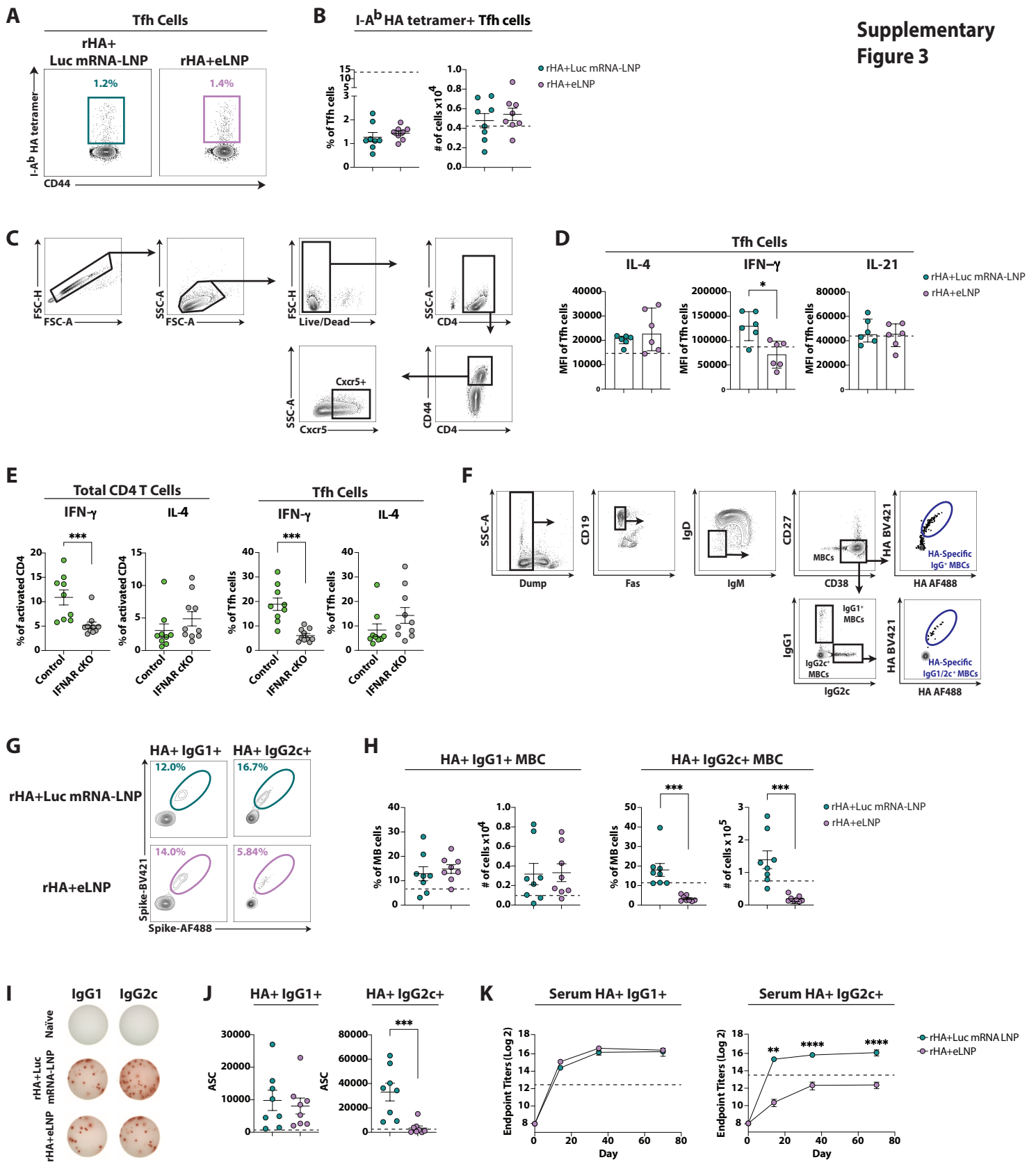

Supplement: 3 — Supplementary Figure 3. The nucleoside-modified mRNA regulates Tfh cell function and B cell responses, related to Figure 3. (A) Representative flow cytometry data of HA-specific Tfh cells (I-Ab HA tetramer+) 7 days post-immunization. (B) HA-specific Tfh cell frequency (Left) and absolute numbers (Right) from A. (C) Gating strategy for sorting Tfh cells (Live, CD4+CD44+Cxcr5+) from purified CD4 T cells. (D) Quantification of IL-4, IFN-γ, and IL-21 mean fluorescence intensity (MFI) on sorted Tfh cells. Tfh cells sorted from dLNs 7 days post-immunization were restimulated with PMA/Ionomycin. (E) Quantification of IL-4+ and IFN-γ+ total CD4 T cells (Left) and unsorted Tfh cells (Right), 7 days post-immunization with RBD mRNA-LNP of control (Cd11c-cre+) or IFNAR cKO (Cd11c-cre Ifnraflox/flox) mice. (F) Gating strategy of HA-specific memory B cell (MBC) populations. (G) Representative flow cytometry data of HA-specific (HA+) IgG1+ (Left) and IgG2c (Right) MBCs, 70-77 days post-immunization with rHA+Luc mRNA-LNP or rHA+eLNP. (H) Quantification of HA+ IgG1+ (Left) and HA+ IgG2c+ (Right) MBC frequency and absolute counts from G. (I) Representative HA-specific (HA+) IgG1+ (Left) and IgG2c+ (Right) antibody-secreting cells (ASCs) measured by ELISPOT, 70-77 days post-immunization. (J) Quantification of HA+ IgG1+ (Left) and HA+ IgG2c+ (Right) ASCs from I. (K) Antigen-specific (HA+) total IgG1+ (Left) and IgG2c+ (Right) antibody levels post-immunization, calculated as endpoint titers. In (A-D, F-K), mice received a single IM immunization with 30 μg of recombinant HA protein (rHA) mixed with 30 μg Luciferase (Luc) mRNA-LNP (rHA+Luc mRNA-LNP) or empty LNP (rHA+eLNP). n = 6-8 mice per group. In (E) mice received a single IM immunization with 30 μg of RBD mRNA-LNP. Statistical analysis: (B, D, E, H, and J), an unpaired two-tailed Mann-Whitney U test was conducted. (K) Two-way ANOVA was performed. In all cases, data were combined from 2-3 independent experiments. Error bars represent [file NIHMS2129750-supplement-2.pdf]

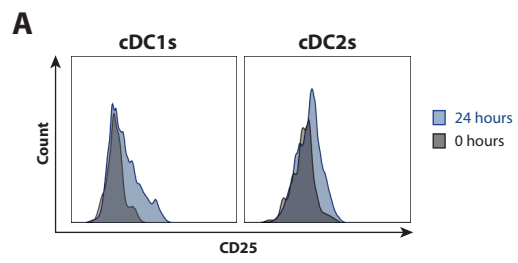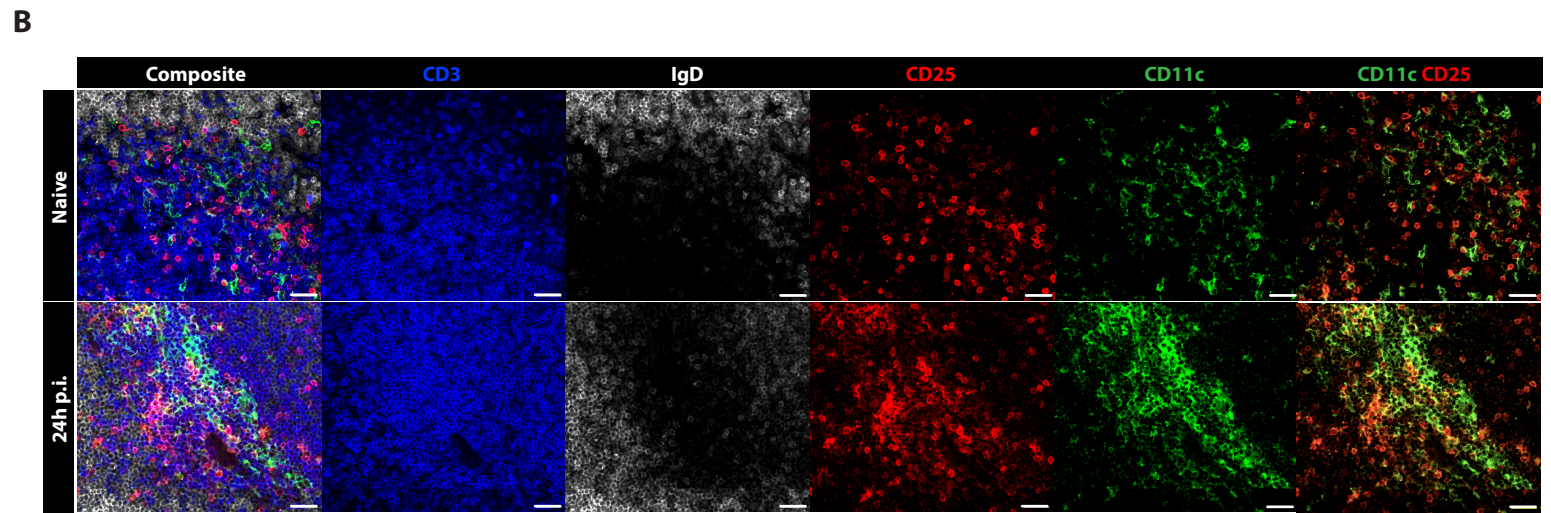

Supplement: 5 — Supplementary Figure 5. LNP drive a pro-Tfh cell signature in cDC2s, related to Figure 5. (A) Representative CD25 expression in cDC1s and cDC2s (as indicated) at baseline (0 hours, dark gray) versus 24 hours post-immunization (blue), determined by flow cytometry. Fluorescent intensity is displayed as count (normalized to mode). (B) Confocal microscopy of dLN 24 hours post-immunization. Images from a representative sample are shown. In A, mice received a single IM immunization containing 20 μg of fluorescent mRNA-LNP. In B, mice were IM immunized with 30 μg of HA mRNA-LNP. [file NIHMS2129750-supplement-7.pdf]

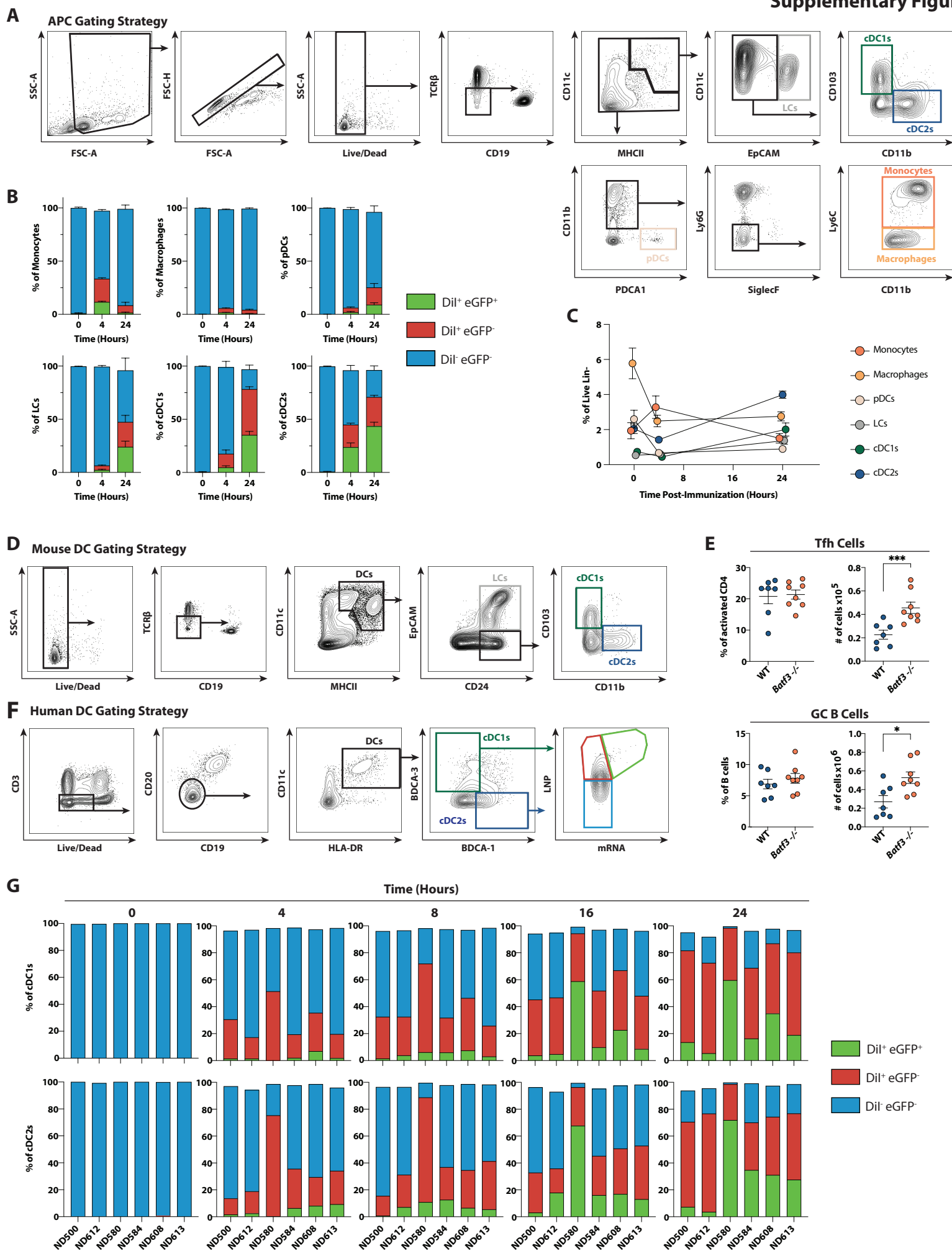

Supplement: 6 — Supplementary Figure 6. Conventional DCs are capable of mRNA-LNP uptake and expression, related to Figure 6. (A) Gating strategy for analyzing APC in mice. Live, TCRβ−CD19− were stratified into LCs (CD11c+MHCII+EpCAM+), cDC1s (CD11c+MHCII+EpCAM−CD103+CD11b−), cDC2s (CD11c+MHCII+EpCAM−CD103−CD11b+), pDCs (CD11b−PDCA1+), inflammatory monocytes (CD11b+PDCA1−Ly6G−SiglecF−Ly6C+), and macrophages (CD11b+PDCA1−Ly6G−SiglecF−Ly6C−). (B) Quantification of LNP binding/uptake (DiI+) and mRNA-encoding protein expression (eGFP+) in the indicated cell populations, as defined in A. Each vertical bar represents 100% of the indicated cell type, and the color represents the portion of cells that are DiI+ eGFP+ (green), DiI+ eGFP− (red), and DiI− eGFP− (blue). Error bars represent SEM. (C) Quantification of cell types from A, represented as a percentage of live and lineage negative (Lin−) cells at the indicated time points. (D) Complete gating strategy for mouse DCs, used to define DC populations in Figure 6B–F. (E) Tfh cell (Top) and GC B cell (Bottom) frequency and absolute numbers were analyzed in wild type (WT) and Batf3−/− mice, 7 days post-immunization with 3 μg Spikevax. (F) Gating strategy for human DCs, used to define DC populations in Figure 6G. (G) Quantification of uptake and expression of eGFP mRNA-LNP-DiI in human DCs from Figure 6G, displayed as individual donors. In (A-D), mice were injected with 20 μg of eGFP mRNA-LNP-DiI. In (E), mice were immunized with 3 μg Spikevax. For (B and C), n = 6 mice per time point. For (E), n = 6 mice per group. Statistical analysis: an unpaired two-tailed Mann-Whitney U test was conducted. Data is combined from two independent experiments. Error bars represent mean + SEM. *p ≤ 0.05, ***p ≤ 0.001. [file NIHMS2129750-supplement-4.pdf]

Supplementary Figure 7

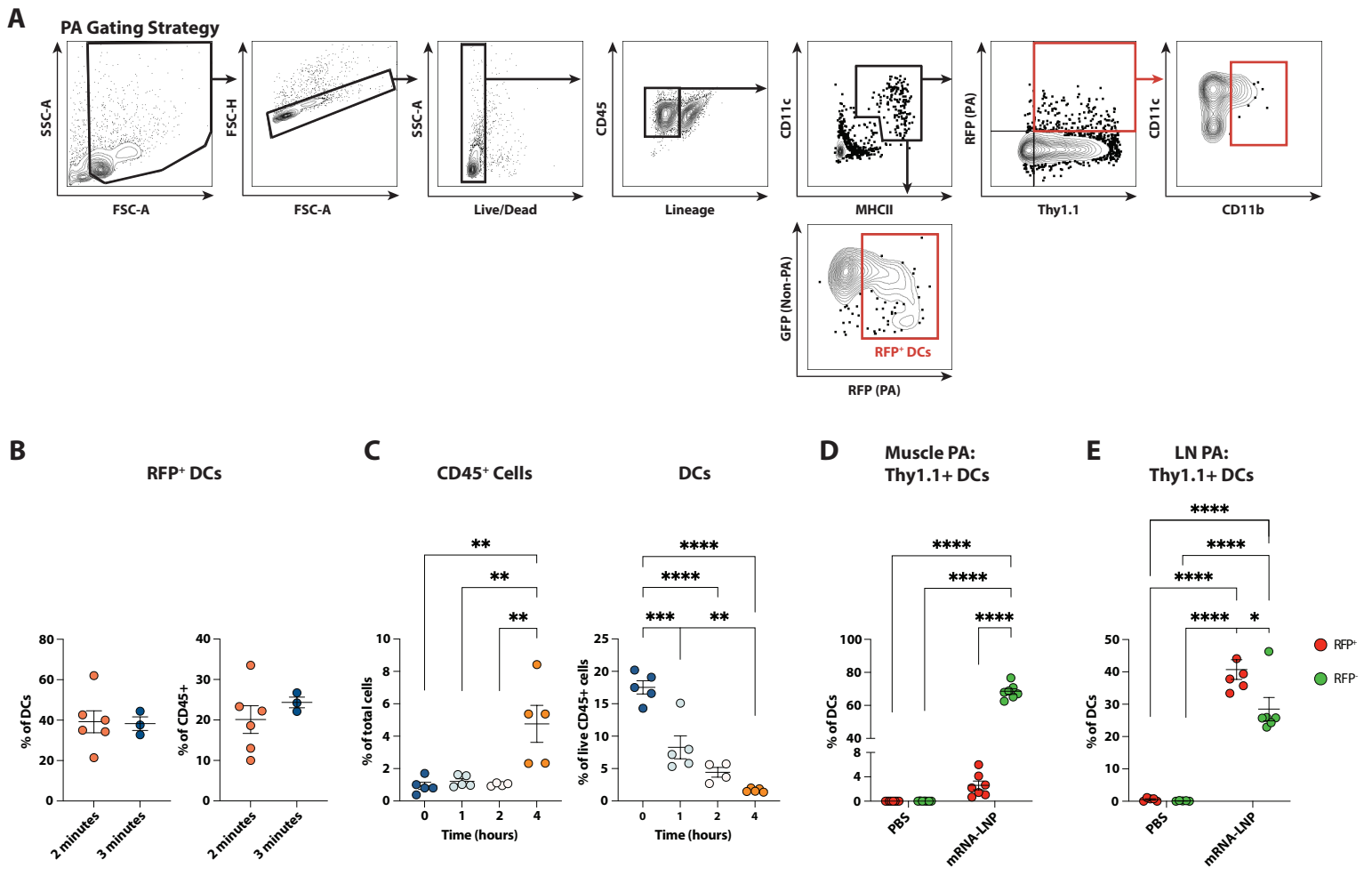

Supplement: 7 — Supplementary Figure 7. Photoactivation of muscle and lymph nodes reveals vaccine uptake within draining LNs, related to Figure 7. (A) Gating strategy for defining DCs after photoactivation (PA). (B) Efficacy of PA in the gastrocnemius muscle. Percentage of RFP+ DCs displayed as a percentage of total DCs (Left) or percentage of total CD45+ (Right) cells in the gastrocnemius immediately following PA. (C) Quantification of CD45+ cell (Left) and DC (Right) frequencies in the gastrocnemius muscle at the indicated time points post-vaccination. (D-E) Quantification of data from Figure 7D and E, displaying RFP+ and RFP− Thy1.1+ DCs as frequency of total DCs from the dLNs of PBS (control) and Thy1.1 mRNA-LNP immunized mice upon muscle (D) or LN (E) PA. In (A and D-E), mice were injected with 30 μg Thy1.1 mRNA-LNP. In (C), mice were injected with 20 μg of eGFP mRNA-LNP-DiI. In (B), n = 3-6 mice per group combined from 2 experiments. In (C-E), n = 5-6 mice per group combined from 2 experiments. Statistical analysis: In (B), an unpaired two-tailed Mann-Whitney U test was conducted. In panels (C-E), a Two-way ANOVA with Tukey’s correction for multiple comparisons was performed. Error bars represent mean + SEM. *p ≤ 0.05, **p ≤ 0.01, ***p ≤ 0.001, ****p ≤ 0.0001. [file NIHMS2129750-supplement-5.pdf]
