# Supplementary material for: Distinct components of mRNA vaccines cooperate to instruct efficient germinal center responses
Source: Cell. Author manuscript; Available in PMC 2026 Feb 6. (PMC12878702; doi:10.1016/j.cell.2025.11.023)

Supplementary Figure 4

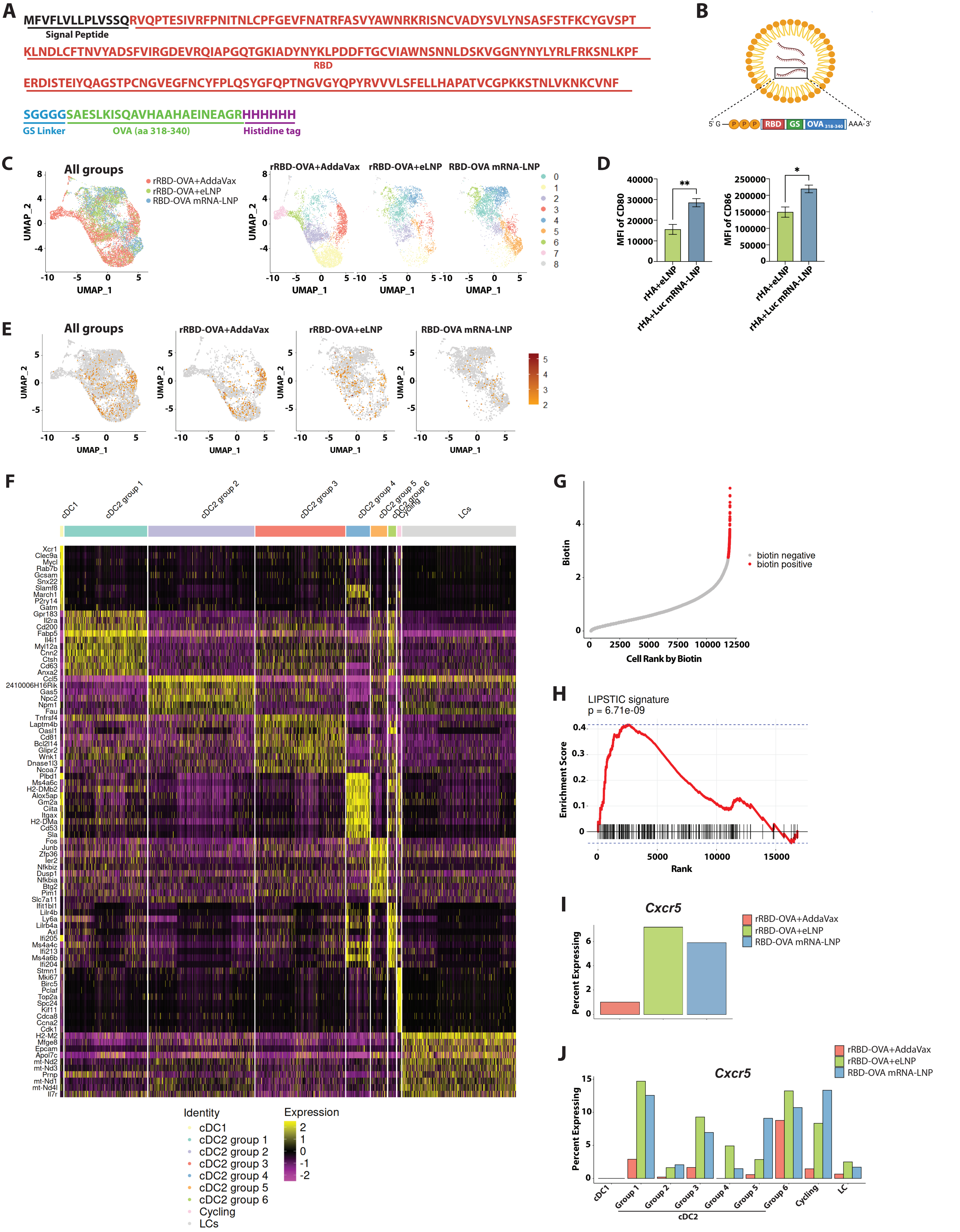

Supplement: 4 — Supplementary Figure 4. LIPSTIC gene signature is enriched in biotin+ DCs, related to Figure 4. (A) Amino acid sequence of the RBD-OVA construct, consisting of a signal peptide (black) to facilitate secretion (absent in the secreted protein), the RBD portion of SARS-CoV-2 Spike (red), a GS linker (blue), the peptide sequence of OVA recognized by OT-II (amino acids 318-340, green) and the hexahistidine-tag (purple) for affinity purification. (B) Schematic of the RBD-OVA mRNA-LNP construct. (C) UMAP clustering of sequencing data from Figure 4 before integration. (D) Quantification of CD80 (Left) and CD86 (Right) mean fluorescence intensity (MFI) in DCs from mice immunized with rHA+eLNP or rHA+Luc mRNA-LNP. (E) Feature plot of biotin expression before integration, displayed as a continuous variable. (F) Heatmap displaying the top 10 differentially regulated genes in each DC group/cluster. (G) Elbow plot of biotin-labeling, where the elbow represents the threshold (2.8) for biotin+ cells. (H) GSEA analysis showing the enrichment of LIPSTIC-signature genes in biotin+ DCs. (I and J) Proportion of Cxcr5-expressing cells in each group (I) and on the different DC clusters (J). In A-C, E-J, Mice were immunized IM with either 30 μg of RBD-OVA mRNA-LNP, 30 μg recombinant RBD-OVA combined with empty LNP (rRBD-OVA+eLNP), or 30 μg recombinant RBD-OVA combined with AddaVax (rRBD-OVA+AddaVax); n = 2 mice per group. In D, mice received a single IM immunization with 30 μg recombinant HA (rHA)+Luc-mRNA-LNP or 30 μg rHA combined with eLNP (rHA+eLNP); n = 6-11 mice per group. Statistical analysis: (D) An unpaired two-tailed Mann-Whitney U test was conducted. Error bars represent mean + SEM. (I) The percentage of positive cells was calculated within each group based on single-cell expression data. *p ≤ 0.05, **p ≤ 0.01. [file NIHMS2129750-supplement-6.pdf]
